# Hispidulin: a potential alternative to vorinostat against HDAC1 for acute myeloid leukemia

**DOI:** 10.1007/s12672-025-03182-y

**Published:** 2025-07-22

**Authors:** Sathyanarayan Balaji, Suvitha Anbarasu, Sudha Ramaiah, Anand Anbarasu

**Affiliations:** 1 Department of Biotechnology, School of Bioscience and Technology (SBST), Vellore Institute of Technology (VIT), Vellore, 632014 Tamil Nadu State India; 2 Medical and Biological Computing Laboratory, School of Bioscience and Technology (SBST), Vellore Institute of Technology (VIT), Vellore, 632014 Tamil Nadu State India; 3 Department of Biosciences, School of Bioscience and Technology (SBST), Vellore Institute of Technology (VIT), Vellore, 632014 Tamil Nadu State India; 4 Medical and Biological Computing Laboratory, Department of Biotechnology, School of Biosciences and Technology (SBST), Vellore Institute of Technology (VIT), Vellore City, 632014 Tamil Nadu State India

**Keywords:** Epigenetic regulator, Phytochemical screening, Pharmacokinetic properties, Molecular dynamics, Interaction energy, Binding free energy

## Abstract

Acute myeloid leukemia (AML) has a survival rate of only 30%, predominantly affecting the bone marrow. AML is characterized by failure of bone marrow function to produce healthy blood cells resulting in significant clinical symptoms such as anemia, shortness of breath, and paleness of skin. This study focuses on elucidating the role of Histone Deacetylase 1 (HDAC1), a critical epigenetic regulator whose dysregulation has been recognized to contribute for AML prognosis. Although vorinostat is a well-established HDAC1 inhibitor, its resistance in cancer cells has necessitated the investigation of anti-leukemic phytochemicals as potential alternative therapeutic agents. A set of 800 unique anti-leukemic phytochemical compounds from 22 Indian plants were screened for pharmacokinetic properties revealed 33 compounds to have inhibitory effects. Further, toxicity screening revealed six compounds hispidulin, kumatakenin, phenyl glycoside, kaempferol, pelargonidin and rohitukine as potential lead candidates. Molecular docking studies indicated hispidulin with a notable binding affinity of -7.8 kcal/mol whereas vorinostat had − 6.51 kcal/mol against HDAC1. Molecular dynamics simulations demonstrated a strong binding affinity of hispidulin to HDAC1, as indicated by an average root mean square deviation (RMSD) of 0.784 nm, an interaction energy (IE) of -208.42 kJ/mol, and a total binding free energy of -28.32 ± 3.23 kcal/mol. In comparison, vorinostat exhibited a higher RMSD of 0.868 nm, a slightly lower IE of -202.04 kJ/mol, and a total binding free energy of -28.39 ± 4.81 kcal/mol. These findings suggest that hispidulin exhibits superior binding stability and interaction strength with HDAC1 relative to vorinostat. Thus, hispidulin may serve as a promising lead compound for HDAC1 modulation, potentially enhancing therapeutic efficacy in the treatment of acute myeloid leukemia (AML).

**Supplementary Information:**

The online version contains supplementary material available at 10.1007/s12672-025-03182-y.

## Introduction

Acute myeloid leukemia (AML) is a hematologic malignancy arising from hematopoietic stem cell (HSC) dysfunction, resulting in uncontrolled proliferation of abnormal myeloid progenitors. This impairs the normal myeloid differentiation and reduced production of functional myeloid lineage cells. Unlike lymphoid leukemias, AML specifically originates from the myeloid lineage. AML is characterized by bone marrow failure, leading to diminished hematopoiesis and significant clinical manifestations. The resulting condition cause severe anemia, presenting as fatigue, dyspnea, and pallor. Additionally, leukemic infiltration disrupts normal blood cell production and may extend to organs such as the central nervous system, muscles, and testes [1]. AML is also referred to as acute myelogenous leukemia or acute nonlymphocytic leukemia, had an estimated 11,220 new cases and 20,800 deaths in the United States as of 2024. According to data from the Surveillance, Epidemiology, and End Results (SEER) program spanning 2014 to 2020, the observed 5-year survival rate for AML patients is 31.9% [2,3].

The blood cell formation occurs by the process of hematopoiesis that relies on the function of numerous genes. Leukemia could occur when there in an alteration in the expression of those genes [4]. AML could be characterized by a combination of genetic mutations and epigenetic changes that lead to unchecked cellular proliferation, impaired differentiation, and inhibition of apoptosis. The epigenetic modifications play a crucial role in leukemogenesis and majorly chromosomal translocations help in the prognosis of AML [5, 6]. The *HDAC1* is one of the major epigenetic regulators that work along with other genes to control the access for transcription factors to access the target gene. Hematopoiesis requires linear orchestration of gene expression at each stage in which epigenetic regulators play a pivotal role in controlling gene expression. Dysregulation of HDAC1 by abnormal expression and HDAC-containing transcriptional complex are involved in the pathogenesis of hematological malignancies [7, 8]. Strategically targeting dysfunctional HDAC1 in AML serves as a promising approach to enhance therapeutic outcomes for AML patients [9].

Histone deacetylases (HDACs) serve as downstream targets of various signaling pathways and are regulated by signaling molecules, particularly kinases, through multiple mechanisms. They play a critical role in the regulation of key cellular processes, including cell cycle progression, differentiation, and tumorigenesis. Dysregulation of HDAC function has been implicated in the pathogenesis of numerous human diseases, such as cancer, neurodegenerative disorders, cardiac hypertrophy, and pulmonary diseases [10]. HDACs comprise 18 isoforms categorized into four distinct classes. Three of these classes—Class I (HDAC1, 2, 3, and 8), Class II (HDAC4, 5, 6, 7, 9, and 10), and Class IV (HDAC11)—are zinc-dependent, whereas Class III, known as sirtuins, is dependent on nicotinamide adenine dinucleotide (NAD⁺) [10].

HDAC1, a member of Class I, consists of 483 amino acids and is primarily localized within the nucleus. It is ubiquitously expressed and functions as a component of various multimeric nuclear complexes associated with transcriptional repression, including COREST, mSin3, and NuRD [11, 12]. In our previous study, HDAC1 was identified as a potential therapeutic target in AML due to its involvement in key leukemia-associated pathways. Additionally, HDAC1 was found to be a central component of the AML gene network, which was constructed based on transcription factors of the *IKZF1* gene, highlighting its epigenetic regulatory role in AML pathogenesis [13]. The *HDAC1* is involved in the deacetylation of lysine residues on histone and non-histone proteins helping in the formation of repressive chromatin resulting in the control of the target gene expression [14]. The *HDAC1* acts on the histone proteins of the target gene to form repressive chromatin, thereby regulating target gene expression. This process results in the increase of histone 3 trimethylation at lysine 27 (H3K27me [3]) and a decrease in lysine 9 acetylation (H3K9ac) indicating the formation of repressed chromatids [15, 16]. This deacetylation of chromatids occurs along with a corepressor complex whose combined action leads to the formation highly condensed structure resulting in the repression of gene transcription [17, 18].

Vorinostat (suberoylanilide hydroximic acid, SAHA) is a FDA approved drug belongs to the class of histone deacetylase inhibitors (HDACi). Vorinostat can induce growth arrest by specific binding to and inhibition of HDAC resulting in acetylation of nucleosomal histones and an activation of gene transcription. Preclinical research on vorinostat has demonstrated that its anti-proliferative effects are linked to the activation of the G2/M cell cycle checkpoint, along with an increase in p21 expression and a decrease in cyclin D1 levels. Additionally, vorinostat induces acetylation of various transcription factors, such as p53, and other proteins, including HSP90 and tubulin [19, 20]. In general, the action of HDAC inhibitors results in the accumulation of acetylated histone and non-histone proteins, which promotes potent antineoplastic effects by altering gene expression. This leads to cell cycle arrest and the induction of apoptosis in tumor cells [21]. Resistance to anti-cancer therapy is a persistent problem in cancer management. Acquired resistance in tumors not only become resistant to initial drug but also develop cross-resistance to other therapies through various epigenetic modifications and molecular mechanisms [22]. Likewise, vorinostat has shown resistance in various cancer therapies. A recent study on cutaneous T-cell lymphoma (CTCL) elucidated that the production of reactive oxygen species (ROS) which cause DNA damage and cell cycle arrest also accelerated the production of interleukin (IL)−2Rα. This overexpression of IL-2Rα leads to over-activation of JK/signal transducer, protein kinase B and mitogen-activated protein kinase (MAPK) signaling events leading to resistance of vorinostat on CTCL cells [23]. A study revealed that vorinostat treatment has increased the expression of phospholipase D1 (*PLD1*), it hydrolyses phospholipid to phosphatidic acid. The upregulation of *PLD1* due to vorinostat could led to invasive migration and proliferation of tumor cell in gliobastoma [24, 25]. Another study on colorectal adenocarcinoma cell lines revealed that acquisition of multidrug resistance protein independent resistance due to vorinostat treatment. This resistance probably emerged due to epigenetic targeting by the compound which is acquired after exposure to vorinostat over time and irreversible. This resistance is associated with key epigenetic cellular responses by HDACi, including loss of acetylation of histones H2A, H2B, H3, and H4, impairment of G2/M checkpoint activation, and the inhibition of caspase-3 and caspase-7 mediated apoptotic pathways [26]. Vorinostat binds to the zinc ion within the catalytic pocket of the protein, thereby inhibiting HDAC1 activity. However, point mutations or structural alterations within the catalytic domain can induce conformational changes that disrupt the interaction between the hydroxamic acid moiety of vorinostat and the zinc ion. These structural modifications can reduce binding affinity and compromise the inhibitory effect of vorinostat, ultimately contributing to resistance [27].

For the treatment of AML, there are limited FDA-approved therapeutics beyond standard chemotherapy. These include Venetoclax and Glasdegib, as well as targeted agents such as Midostaurin and Gilteritinib, which inhibit Fms-like tyrosine kinase 3 (FLT3). Additionally, Enasidenib and Ivosidenib target isocitrate dehydrogenase (IDH) mutations, while Gemtuzumab Ozogamicin, a CD33-positive monoclonal antibody, has also been approved [28–30]. Several other targeted therapies have been authorized for AML treatment, reflecting advancements in precision medicine for this malignancy [31, 32].

To overcome vorinostat resistance against HDAC, this study examined phytochemicals from 22 plants with anti-leukemic properties and were subjected to ADMET screening for a potential drug candidate. Molecular docking of the drug hits against HDAC1 protein was performed to analyze the interaction profile between the protein-ligand complexes. This research also involves molecular dynamic (MD) simulation study from which the stability of the protein and the compound is confirmed using the root mean square deviation (RMSD), root mean square fluctuation (RMSF), radius of gyration (Rg), interaction energy (IE), solvent accessible surface area (SASA) and Molecular Mechanics Poisson Boltzman Surface Area (MM-PBSA) analysis. Screening a potent phytochemical compound could address the traditional limitation of resistance and thereby improve the treatment efficiency in AML.

## Materials and methods

### Data retrieval

The phytochemicals were chosen based on their anti-leukemic property [33, 34]. The data of phytochemicals present in each of the plants were retrieved from the Indian Medicinal Plants, Phytochemistry And Therapeutics (IMPPAT) database (https://cb.imsc.res.in/imppat/), LOTUS database (https://lotus.naturalproducts.net/) and literature review [35–38]. The two databases are manually curated database which comprises phytochemicals along with their standard chemical identifiers and structural information. The Simplified molecular-input line entry system (SMILES) and 3D structure of the compounds were retrieved from the PubChem database (https://pubchem.ncbi.nlm.nih.gov/) [39]. The 3D structure of the HDAC1 protein (PDB ID: 4BKX) was retrieved from the Research Collaboratory for Structural Bioinformatics Protein Data Bank (RCSB PDB) (https://www.rcsb.org/) [40].

### ADME (Absorption, Distribution, Metabolism, and Excretion) screening

The phytochemical screening of the retrieved compounds for ADME properties was performed using the SwissADME webserver (http://www.swissadme.ch/). It is a web server that allows to compute the ADME parameters, drug likeness nature and pharmacokinetic properties for drug discovery [41]. The parameters that were used to screen were high gastrointestinal (GI) absorption, zero violations in Lipinski rule and lead likeness, topological polar surface area (TPSA) between 60 and 140, high solubility, and very low synthetic accessibility (SA) in a sequential manner.

### Toxicity prediction

The toxicity of the compound that passed ADME screening was predicted using ProTox-3.0 webserver (Prediction of Toxicity of Chemicals) (https://comptox.charite.de/protox3/) designed for the prediction of toxicity of chemicals. This server incorporates molecular similarity, pharmacophores, fragment propensities, and machine learning models for the prediction of various toxicity that could be caused by the compound of interest [42].

### Molecular docking

The active site of the HDAC1 was found based on the modified peptide fragment (HK16Hx) bound to the protein structure of PDB ID: 5ICN with nearly 100% occupancy and used for further docking analysis [43]. The protein structure (PDB ID: 4BKX) retrieved was prepared for docking using the UCSF Chimera tool, an interactive visualization and molecular structure analysis tool in which the heteroatoms, water molecules and previously bound small molecules in the protein were removed [44]. The non-bonded atoms were reconstructed using Swiss PDB Viewer (SPDBV) tool, a user-friendly interface to analyze proteins [45]. The vorinostat was employed as positive control (PC) and glycerol, a low affinity compound was utilized as negative control (NC) due to its lack of specific binding affinity. It’s high polar nature and absence of functional group capable of strong interactions limit them from binding to HDAC1 or other proteins, making it as a reference compound for assessing non-specific interactions [46]. The ADMET-screened phytochemical compounds, PC, and NC were converted to PDB files using Open Babel, a chemical structure format conversion tool [47]. The AutoDock 4.2 tool was employed to dock the compounds to the active site of HDAC1. This tool is an automated docking software that helps in predicting the binding affinities, inhibition constant (Ki), and the number of hydrogen bonds formed between the small molecules and the target protein [48]. The AutoDock 4.2 is an open source tool that supports flexible docking and parallel processing enhanced by GPU acceleration, employs comprehensive scoring functions, and has less software limitations compared to other docking softwares [49]. The receptor and ligands were prepared using the AutoDock 4.2 tool. A site specific docking was performed by centering the grid box of 60 × 70 × 70 Å [3] on the active sites of the receptor with a spacing of 0.375Å. The AutoDock application was then executed with Lamarckian genetic algorithm scoring, a population size of 150 for 10 runs following the default AutoDock parameter. The Lamarckian algorithm provides crucial and effective method to search for optimal binding conformation of a ligand within the receptor protein using explicit local search operator mechanism that helps in predicting accurate protein-ligand interactions. This algorithm differs from conventional genetic algorithms by integrating both genetic evolution and local search optimization, leading to efficient and enhanced performance [50]. The grid box was strategically designed to precisely encapsulate the active site residues, minimizing interactions with non-target residues and ensuring optimal ligand binding. The lowest binding energy conformation was selected and used to make protein-ligand complex for all the phytochemicals. The 2D interaction profiles of the complexes were analyzed using LigPlot + tool [51]. The 3D interaction profiles were visualized and analyzed using UCSF chimera.

### MD simulation

The protein-ligand complexes obtained from docking analysis was subjected to MD simulation for 100 ns using GROMACS version 2024 [52]. The CHARMM-36 all-atoms force field was used to define the topology for the protein [53]. The hydrogens were added to the ligand molecule using Avogadro software, an advanced molecule editing and visualizing tool [54]. The ligand topology was generated using CHARMM General Force Field (CGenFF) webserver (https://cgenff.silcsbio.com/) which is an automated server that performs atom typing, assignment of parameters, and charges to the ligand molecule [55]. The protein-ligand complex was immersed in a 1 Å cubic box and solvated with water as in TIP3P model. The system was neutralized with Cl^−^ ions. Further, energy minimization was performed with the steepest descent minimization algorithm through 50,000 steps. The energy minimized system was then equilibrated through 100 picoseconds (ps) for temperature stabilization and 100ps for constant pressure stabilization at 300 K. Then, the protein-ligand system was put through a 100 nanosecond (ns) of MD simulation with step time of 2 femtosecond (fs) under 300 K and 1 bar pressure without periodic boundary conditions (PBC) in all directions. After the simulation, the protein-ligand trajectories were analyzed using GROMACS (gmx) tools such as rms, rmsf, gyrate, hbond, energy, sasa and xmgrace v5.1.25. The gmx_rms helps calculating the RMSD of the protein backbone-ligand complex, gmx_rmsf calculates the RMSF to measure the flexibility of the protein, gyrate was used to measure the compactness of the protein, hbond aids in calculating the number of hydrogen bonds (H-bonds) formed during the course of simulation and energy tool was used to calculate the non-bonded short-range interaction energy of the protein-ligand system utilizing two energy modules, sasa helps to compute the surface area of the protein that could form contact with the solvent molecules, Lennard-Jones energy (LJ) and Coulomb interaction energy (Coul). The interaction energy (E_int_) is the sum of LJ and Coul. The xmgrace was used to plot the MD resultant trajectories.

### Binding free energy calculation

The MM-PBSA method was used for the calculation of binding free energy for the protein-ligand complexes. An intergrated GROMACS tool called g_mmpbsa was used for the MMPBSA calculation of the trajectory obtained. The gmx_MMPBSA_ana visualization tool was used to generate graphs and obtain the energy parameter values of the complex [56, 57]. The gmx_MMPBSA is a high-throughput MD simulation binding energy calculation tool based on molecular mechanics. It helps to calculate the molecular mechanics potential energy (E_MM_) which is a sum of all bonded and non-bonded interaction energy.

$$\begin{aligned}\mathrm{E}_\mathrm{MM}=\Delta\mathrm{E}_\mathrm{bonded}+\Delta\mathrm{E}_\mathrm{non\text{-}bonded}=\Delta\mathrm{E}_\mathrm{bonded}+\left(\Delta\mathrm{E}_\mathrm{vdw}+\Delta\mathrm{E}_\mathrm{elec}\right)\end{aligned}$$

Where E_vdw_ is van der Waals interaction energy and E_elec_electrostatic energy. The binding free energy of the protein-ligand complex (G_protein-ligand_) is given by

2 $$\begin{aligned}\Delta \mathrm{G}_\mathrm{protein\text{-}ligand}=\Delta\mathrm{E}_\mathrm{MM}-\mathrm{T}\Delta\mathrm{S}+\left(\Delta\mathrm{G}_\mathrm{solvation} \right) \end{aligned}$$

Here, ΔG_solvation_ is the sum of polar (ΔE_GB_) and non-polar free energy (ΔE_surf_) surface area dependent which is calculated using implicit solvent model. The terms TS refers to the temperature and entropy which contributes to free energy [58–60].

## Results

### ADME screening

Phytochemical analysis of 22 plants yielded 800 unique compounds, for which SMILES notations and PubChem IDs were retrieved from phytochemical and PubChem databases, respectively (Table S1). Using the SwissADME server all the 800 compounds were screened based on the GI absorption, Lipinski’s non-violations, and lead-likeness, TPSA, and SA parameters. This screening process identified 33 lead candidates for toxicity prediction, as the remaining compounds were excluded due to Lipinski’s rule violations, high SA, low GI absorption, poor lead likeness properties and out of range TPSA values. The details of 33 compounds are provided in Table 1 and the ADME properties of the screened candidates are documented in Table 2.

### Toxicity screening

The toxicity prediction using the ProTox 3.0 server screened the 33 compounds from the ADME screening and passed out six compounds hispidulin, rohitukine, kaempferol, kumatakenin, pelargonidin and phenyl glucoside with inactive hepatotoxicity, carcinogenicity, mutagenicity, immunotoxicity, and cytotoxicity. The toxicity scores for the above parameters of the final hits are tabulated in Table 3.

**Table 1:** Structure and database identifier details of the lead compounds

Compound name	IMMPAT ID	Pubchem ID	Canonical SMILES	Formula
Quercetin	IMPHY004619	5280343	Oc1cc(O)c2c(c1)oc(c(c2 = O)O)c1ccc(c(c1)O)O	C15H10O7
Thiamine	IMPHY000005	1130	OCCc1sc[n+](c1C)Cc1cnc(nc1N)C	C12H17N4OS+
Melanin	IMPHY012901	6325610	Cc1c2[nH]cc3c2c(c(= O)c1 = O)c1c[nH]c2c1c3c(= O)c(= O)c2C	C18H10N2O4
Naringetol	IMPHY010550	439246	Oc1ccc(cc1)[C@@H]1CC(= O)c2c(O1)cc(cc2O)O	C15H12O5
Luteolin	IMPHY004660	5280445	Oc1cc(O)c2c(c1)oc(cc2 = O)c1ccc(c(c1)O)O	C15H10O6
Apigenin	IMPHY004661	5280443	Oc1ccc(cc1)c1cc(= O)c2c(o1)cc(cc2O)O	C15H10O5
Kaempferol	IMPHY004388	5280863	Oc1ccc(cc1)c1oc2cc(O)cc(c2c(= O)c1O)O	C15H10O6
Coniferin	Literature referenced	5280372	OCC = Cc1ccc(c(c1)OC)OC1OC(CO)C(C(C1O)O)O	C16H22O8
Vernomenin	Literature referenced	442324	C = CC12CC3C(C(C1C(= C)C(= O)OC2)O)C(= C)C(= O)O3	C15H16O5
Rohitukine	Lotus database	13422573	CN1CC[C@@H]([C@@H](C1)O)c1c(O)cc(c2c1oc(C)cc2 = O)O	C16H19NO5
Skullcapflavone I	IMPHY004375	5320399	COc1cc(O)c2c(c1OC)oc(cc2 = O)c1ccccc1O	C17H14O6
Wogonin	IMPHY005530	5281703	COc1c(O)cc(c2c1oc(cc2 = O)c1ccccc1)O	C16H12O5
Baicalein	IMPHY005607	5281605	Oc1cc2oc(cc(= O)c2c(c1O)O)c1ccccc1	C15H10O5
Hispidulin	IMPHY005442	5281628	COc1c(O)cc2c(c1O)c(= O)cc(o2)c1ccc(cc1)O	C16H12O6
Norcepharadione B	IMPHY003329	189168	COc1cc2c3c(c1OC)c1ccccc1cc3[nH]c(= O)c2 = O	C18H13NO4
Piperolactam A	IMPHY012679	3081016	COc1cc2c(= O)[nH]c3c2c(c1O)c1ccccc1c3	C16H11NO3
Aristolactam AII	IMPHY012353	148657	COc1c(O)cc2c3c1c1ccccc1cc3[nH]c2 = O	C16H11NO3
Rhamnazin	IMPHY004329	5320945	COc1cc(O)c2c(c1)oc(c(c2 = O)O)c1ccc(c(c1)OC)O	C17H14O7
Kumatakenin	IMPHY004360	5318869	COc1c(oc2c(c1 = O)c(O)cc(c2)OC)c1ccc(cc1)O	C17H14O6
Coniferin	IMPHY004603	5280372	OC/C = C/c1ccc(c(c1)OC)O[C@@H]1O[C@H](CO)[C@H]([C@@H]([C@H]1O)O)O	C16H22O8
Isorhamnetin	IMPHY008724	5281654	COc1cc(ccc1O)c1oc2cc(O)cc(c2c(= O)c1O)O	C16H12O7
Sakuranetin	IMPHY012232	73571	COc1cc2O[C@@H](CC(= O)c2c(c1)O)c1ccc(cc1)O	C16H14O5
Phenyl glucoside	IMPHY001894	11701599	OC[C@H]1OC(Oc2ccccc2)[C@@H]([C@H]([C@@H]1O)O)O	C12H16O6
Herbacetin	IMPHY004393	5280544	Oc1ccc(cc1)c1oc2c(O)c(O)cc(c2c(= O)c1O)O	C15H10O7
Okanin	Lotus database	5281294	Oc1ccc(cc1O)/C = C/C(= O)c1ccc(c(c1O)O)O	C15H12O6
Pelargonidin	IMPHY003437	440832	Oc1ccc(cc1)c1[o+]c2cc(O)cc(c2cc1O)O	C15H11O5+
Eriodictyol	IMPHY004038	440735	Oc1cc2O[C@@H](CC(= O)c2c(c1)O)c1ccc(c(c1)O)O	C15H12O6
Artecanin	IMPHY001062	442147	C = C1C(= O)O[C@H]2[C@H]1CC[C@@]([C@@]13[C@@H]2[C@@]2(C)O[C@H]2[C@H]3O1)(C)O	C15H18O5
Santin	IMPHY012746	5281695	COc1ccc(cc1)c1oc2cc(O)c(c(c2c(= O)c1OC)O)OC	C18H16O7
secotanapartholide A	IMPHY015945	10356188	CC(= O)CC[C@H]1 C(= C)C(= O)O[C@@H]1[C@@H]1 C(= O)C = C[C@@]1(C)O	C15H18O5
5,6-Dihydroxy-3,7,4’-trimethoxyflavone	IMPHY015946	10043097	COc1ccc(cc1)c1oc2cc(OC)c(c(c2c(= O)c1OC)O)O	C18H16O7
secotanapartholide B	IMPHY016033	10265551	CC(= O)CC[C@H]1 C(= C)C(= O)O[C@@H]1[C@@H]1 C(= O)C = C[C@]1(C)O	C15H18O5
Acacetin	Lotus database	5280442	COc1ccc(cc1)c1cc(= O)c2c(o1)cc(cc2O)O	C16H12O5

**Table 2:** ADME properties of phytochemicals screened

Compound name	MW	TPSA	ESOL class	GI absorption	Lipinski #violations	Leadlikeness #violations	Synthetic accessibility
Quercetin	302.24	131.36	Soluble	High	0	0	3.23
Thiamine	265.35	104.15	Soluble	High	0	0	2.99
Melanin	318.28	99.86	Very soluble	High	0	0	2
Naringetol	272.25	86.99	Soluble	High	0	0	3.01
Luteolin	286.24	111.13	Soluble	High	0	0	3.02
Apigenin	270.24	90.9	Soluble	High	0	0	2.96
Kaempferol	286.24	111.13	Soluble	High	0	0	3.14
Coniferin	342.34	128.84	Very soluble	High	0	0	4.56
Vernomenin	276.28	72.83	Soluble	High	0	0	4.27
Rohitukine	305.33	94.14	Soluble	High	0	0	3.78
Skullcapflavone I	314.29	89.13	Soluble	High	0	0	3.37
Wogonin	284.26	79.9	Moderately soluble	High	0	0	3.15
Baicalein	270.24	90.9	Moderately soluble	High	0	0	3.02
Hispidulin	300.26	100.13	Soluble	High	0	0	3.12
Norcepharadione B	307.3	68.39	Moderately soluble	High	0	0	2.35
Piperolactam A	265.26	62.32	Soluble	High	0	0	2.02
Aristolactam AII	265.26	62.32	Soluble	High	0	0	1.96
Rhamnazin	330.29	109.36	Soluble	High	0	0	3.41
Kumatakenin	314.29	89.13	Soluble	High	0	0	3.34
Coniferin	342.34	128.84	Very soluble	High	0	0	4.56
Isorhamnetin	316.26	120.36	Soluble	High	0	0	3.26
Sakuranetin	286.28	75.99	Soluble	High	0	0	3.11
Phenyl glucoside	256.25	99.38	Very soluble	High	0	0	4.13
Herbacetin	302.24	131.36	Soluble	High	0	0	3.2
Okanin	288.25	118.22	Moderately soluble	High	0	0	2.72
Pelargonidin	271.24	94.06	Soluble	High	0	0	3.04
Eriodictyol	288.25	107.22	Soluble	High	0	0	3.11
Artecanin	278.3	71.59	Very soluble	High	0	0	4.97
Santin	344.32	98.36	Moderately soluble	High	0	0	3.48
secotanapartholide A	278.3	80.67	Very soluble	High	0	0	4.39
tanetin	344.32	98.36	Moderately soluble	High	0	0	3.5
secotanapartholide B	278.3	80.67	Very soluble	High	0	0	4.39
Acacetin	284.26	79.9	Moderately soluble	High	0	0	2.98

**Table 3:** Toxicity prediction of final ADME screened compounds

Compound name	Toxicity target	Toxicity prediction	Probability
Hispidulin	Hepatotoxicity	Inactive	0.72
	Carciginocity	Inactive	0.68
	Immunotoxicity	Inactive	0.72
	Mutagenecity	Inactive	0.94
	Cytotoxicity	Inactive	0.95
Rohitukine	Hepatotoxicity	Inactive	0.9
	Carciginocity	Inactive	0.61
	Immunotoxicity	Inactive	0.75
	Mutagenecity	Inactive	0.65
	Cytotoxicity	Inactive	0.71
Kaemferol	Hepatotoxicity	Inactive	0.68
	Carciginocity	Inactive	0.72
	Immunotoxicity	Inactive	0.96
	Mutagenecity	Inactive	0.52
	Cytotoxicity	Inactive	0.98
Kumatakenin	Hepatotoxicity	Inactive	0.7
	Carciginocity	Inactive	0.62
	Immunotoxicity	Inactive	0.71
	Mutagenecity	Inactive	0.86
	Cytotoxicity	Inactive	0.84
Phenyl glucoside	Hepatotoxicity	Inactive	0.93
	Carciginocity	Inactive	0.81
	Immunotoxicity	Inactive	0.94
	Mutagenecity	Inactive	0.76
	Cytotoxicity	Inactive	0.86
Pelargonidin	Hepatotoxicity	Inactive	0.71
	Carciginocity	Inactive	0.69
	Immunotoxicity	Inactive	0.97
	Mutagenecity	Inactive	0.60
	Cytotoxicity	Inactive	0.94

### Molecular docking

**Fig. 1:**
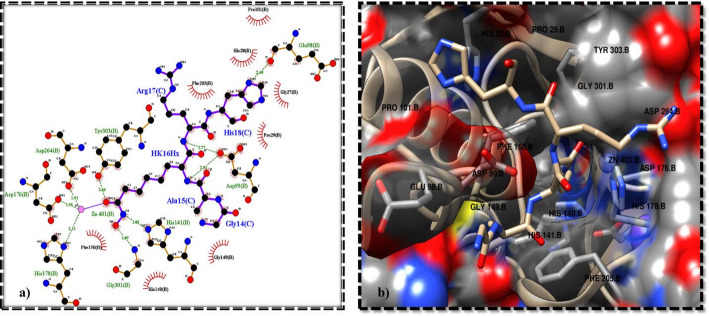
Active site interaction profile of HDAC1. (**a**) 2D interaction profile of synthetic peptide and HDAC1 at the active site. The green dotted lines indicate hydrogen bond formation and the brown lines indicate hydrophobic interactions. (**b**) 3D interaction of the synthetic peptide bound to HDAC1 at the active site

The active site residues include HIS178, ASP176, ASP264, GLY301, GLY301, TYR303, HIS141, ASP99, GLU98, PHE150, HIS140, GLY149, PRO29, HIS28, GLY27, PHE205, and PRO101 with zinc atom inside the deep pocket (Fig. 1a and b) [43]. The HDAC1 residues interacting with the zinc atom were ASP176, HIS178, and ASP264.

**Fig. 2:**
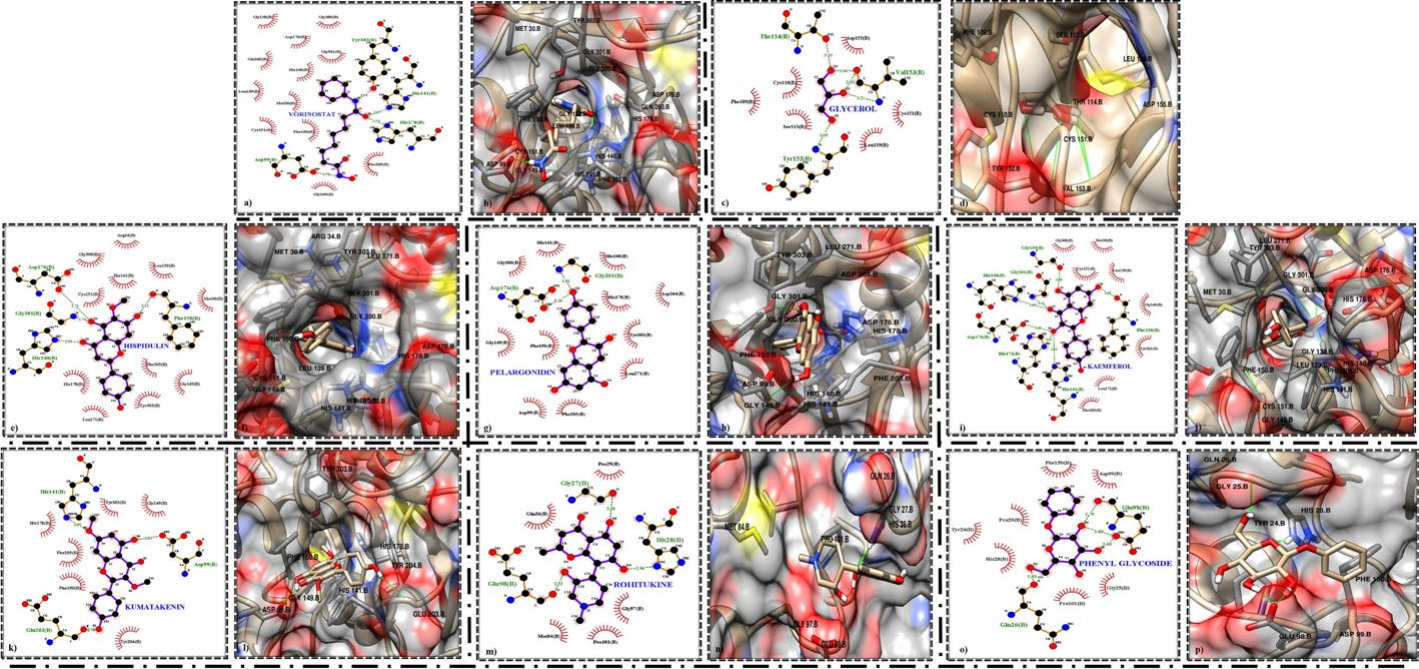
Molecular docking interaction profile. **a**, **b**– 2D and 3D interaction profile of Vorinostat (Positive Control).** c**, **d**– 2D and 3D interaction profile of Glycerol (Negative control). **e**, **f**– 2D and 3D interaction profile of Hispidulin. **g**, **h**– 2D and 3D interaction profile of Pelargonidin. i, j– 2D and 3D interaction profile of Kaempferol.** k**, **l**– 2D and 3D interaction profile of Kumatakenin. **m**, **n**– 2D and 3D interaction profile of Rohitukine. **o**, **p**– 2D and 3D interaction profile of Phenyl glycoside. The green dotted lines indicate hydrogen bond formation, the brown lines indicate hydrophobic interactions and the green line between the ligand and protein in 3D profile indicates the hydrogen bond interaction

The molecular docking analysis against HDAC1 was performed for the six lead compounds that were obtained from the ADMET screening, PC, and NC. The docking analysis was performed for the active site residues predicted using the 2D interaction profile of the crystallized protein and found that all six compounds were well bound to the catalytic tunnel except for rohitukine and phenyl glycoside which were bound adjacent to the catalytic cavity. Among the six lead candidates hispidulin had the highest binding affinity of −7.8 kcal/mol. The other compounds binding affinities are as follows pelargonidin (−6.81 kcal/mol) kaempferol (−5.92 kcal/mol), kumatakenin (−5.64 kcal/mol), phenyl glycoside (−4.32 kcal/mol), and rohitukine (−5.44 kcal/mol). The PC showed − 6.51 kcal/mol binding affinity and Ki 16.93 μm whereas the NC glycerol showed very low affinity of −2.75 kcal/mol and Ki 9.56 mM. Hispidulin is the only compound among the six with the lowest Ki of 1.91 μm (Table 4). The interaction profile analysis revealed that all compounds bound to amino acid residues within the active site, specifically interacting with residues coordinated to the zinc ion. Notably, the majority of interactions were observed with ASP99, HIS178, GLY300, GLY301, and TYR303, which are key residues within the catalytic cavity. In this docking analysis the interaction profiles of PC, hispidulin and pelargonidin shared similar interacting residues. All the other compounds were interacting with HDAC1 either by conventional hydrogen bond or by hydrophobic interactions. In particular, the residues interacting with hispidulin were ASP176 and GLY301 forming hydrogen bonds, significantly HIS178, and TYR303 were interacting hydrophobically with hispidulin. With pelargonidin, significant residues like ASP99, ASP176, HIS178, and ASP264 exhibited hydrophobic interactions. The 2D and 3D interactions of the all the compounds with HDAC1 are depicted in Fig. 2a to p.

**Table 4:** Docking interaction profile of ADMET screened phytochemical compounds

Active site	Compound name	Binding energy (Kcal/mol)	Inhibition constant (Ki)	No of hydrogen bonds target-compound	Amino acid involved in interaction
GLY27,HIS28,PRO29, GLU98,ASP99, PRO101,HIS140, HIS141,GLY149,PHE150, ASP176,HIS178, PHE205,ASP264,GLY301,GLY302,TYR303	Vorinostat	−6.51	16.93 µM	4	MET30,ASP99,GLY138,LEU139,HIS140,HIS141,GLY149,PHE150,CYS151,ASP176,HIS178,PHE205,GLN260,GLY300,GLY301,TYR303
	Hispidulin	−7.8	1.91 µM	4	MET30,ARG34,LEU139,HIS140,HIS141,GLY149,PHE150,CYS151,ASP176,HIS178,PHE205,LEU271,GLY300,GLY301,TYR303
	Pelargonidin	−6.81	10.18 µM	1	ASP99,HIS140,HIS141,GLY149,PHE150,ASP176,HIS178,PHE205,ASP264,LEU271,GLY300,GLY301,TYR303
	Kaemferol	−5.92	45.41 µM	6	MET30,GLY138,LEU139,HIS140,HIS141,GLY149,PHE150,CYS151,ASP176,HIS178,PHE205,LEU271,GLY300,GLY301,TYR303
	Kumatakenin	−5.64	73.37 µM	3	ASP99,HIS141,GLY149,PHE150,HIS178,GLN203,TYR204,PHE205,TYR303
	Rohitukine	−5.44	102.24 µM	1	GLN26,GLY27,HIS28,PRO29,MET84,GLY97,GLU98,PRO101
	Phenyl glycoside	−4.32	680.61 µM	2	TYR24,GLY25,GLN26,HIS28,PRO29,GLU98,ASP99,PHE150
	Glycerol	−2.75	9.56 Mm	3	PHE109,CYS110,SER113,THR114,LEU139,CYS151,TYR152,VAL153,ASP155

### MD simulation

**Fig. 3:**
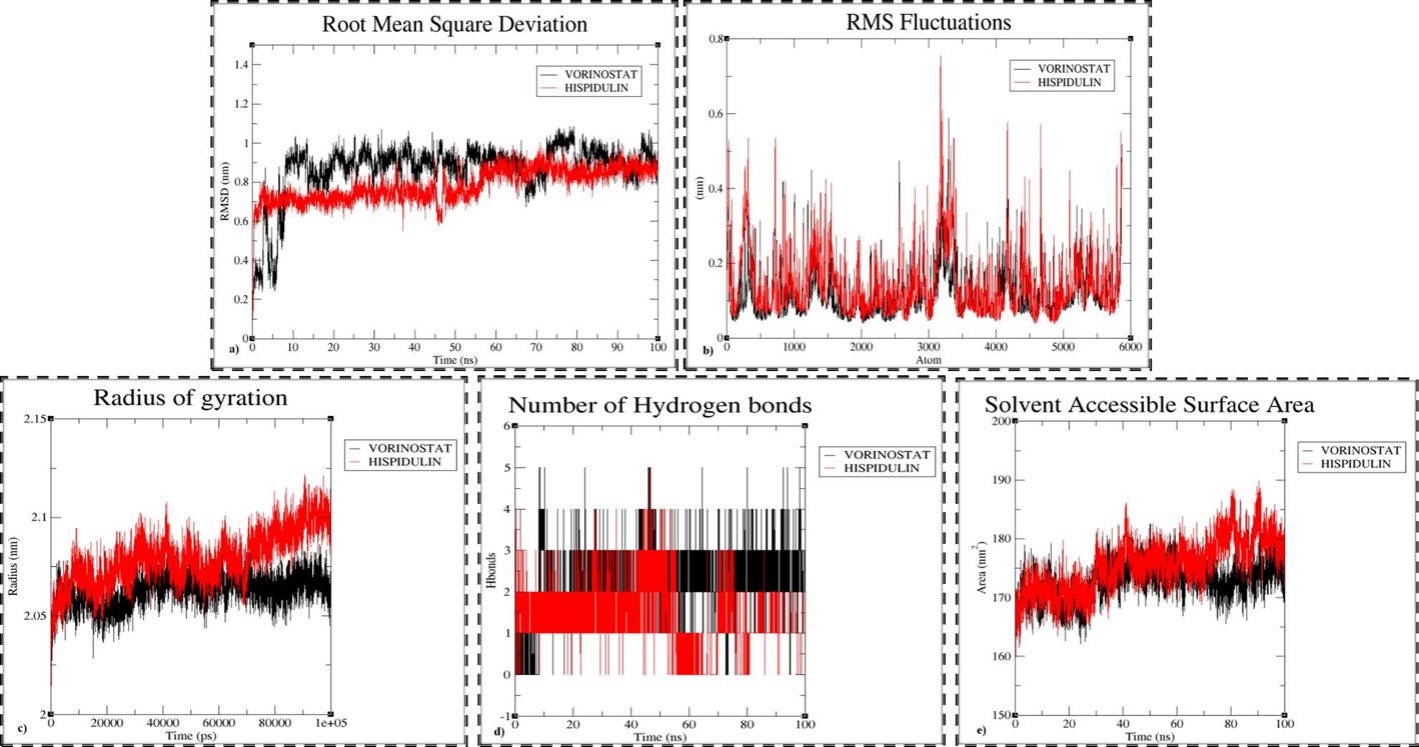
Post-MD simulation trajectory analysis of HDAC1-VOR and HDAC1-HIS complexes over the 100 ns simulation period. (**a**) RMSD plot (**b**) RMSF plot, (**c**) Rg plot of HDAC1, (**d**) H-bond plot between, (**e**) SASA plot

The MD simulation for HDAC1-vorinostat (HDAC1-VOR), HDAC1-hispidulin (HDAC1-HIS), and HDAC1-pelargonidin (HDAC1-PEL) complexes were performed for 100ns, and RMSD, RMSF, Rg, and Hbond were calculated to study the structural changes that has occurred during the simulation with respect to both protein and ligands. The binding interaction energies of the complex was also calculated. The RMSD of HDAC1-VOR complex was 0.8 ± 0.3 nm with an average RMSD of 0.868 nm and there were fluctuations observed. The RMSD obtained for the HDAC1-HIS was 0.7 ± 0.2 nm at the end of 100 ns with an average of 0.784 nm. The RMSD analysis of the HDAC1-HIS complex revealed a stable region between 10 ns and 40 ns, as well as from 50 ns to 100 ns, with minor fluctuations observed between 40 ns and 50 ns. In comparison, the HDAC1-VOR complex exhibited greater fluctuations throughout the simulation. Overall, the HDAC1-HIS complex demonstrated minimal structural deviations relative to the HDAC1-VOR complex (Fig. 3a). The RMSD of HDAC1-PEL showed maximum fluctuations during simulation with an average of 1.26 nm (Fig S1). The RMSF analysis demonstrated that the fluctuations of the complexes were stable through the simulation with least fluctuation at 0.1 ± 0.1 nm and a maximum of 0.75 ± 0.1 nm (Fig. 3b). The maximum fluctuating atom was comprised in LEU271 which is coiled region in HDAC1 structure. The Rg value of the complexes were similar over the course of 100 ns simulation time. The Rg values were ranging from 2 nm to 2.1 nm throughout the simulation (Fig. 3c). From H-bond analysis, for HDAC1-VOR the number of h-bonds were two, in HDAC1-HIS only one H-bond was formed and in HDAC1-PEL there was no H-bond formed initially at the end of simulation respectively **(**Fig. 3d).The H-bond analysis showed that the H-bond interactions were intact during the simulation course except for HDAC1-PEL complex. From the SASA analysis, HDAC1-VOR and HDAC1-PEL complexes exhibit similar solvent-accessible surface areas, with average values of 172.90 nm² and 170.03 nm², respectively. In comparison, the HDAC1-HIS complex has a slightly higher average SASA of 175.45 nm². Despite this, the SASA fluctuations for HDAC1-HIS remain stable throughout the simulation (Fig. 3e).The potential energy of the complex was calculated by considering both the Coul and LJ of the complexes. The energy tool gave a total interaction energy of HDAC1-VOR: −202.04 kJ/mol, HDAC1-HIS: −208.42 kJ/mol, and HDAC1-PEL: −92.76 kJ/mol respectively. The interaction energy of the complexes indicated the highest binding affinity with HDAC1. The post MDS analysis of HDAC1-PEL complex is depicted in Fig S1.

### Binding free energy calculation

**Fig. 4:**
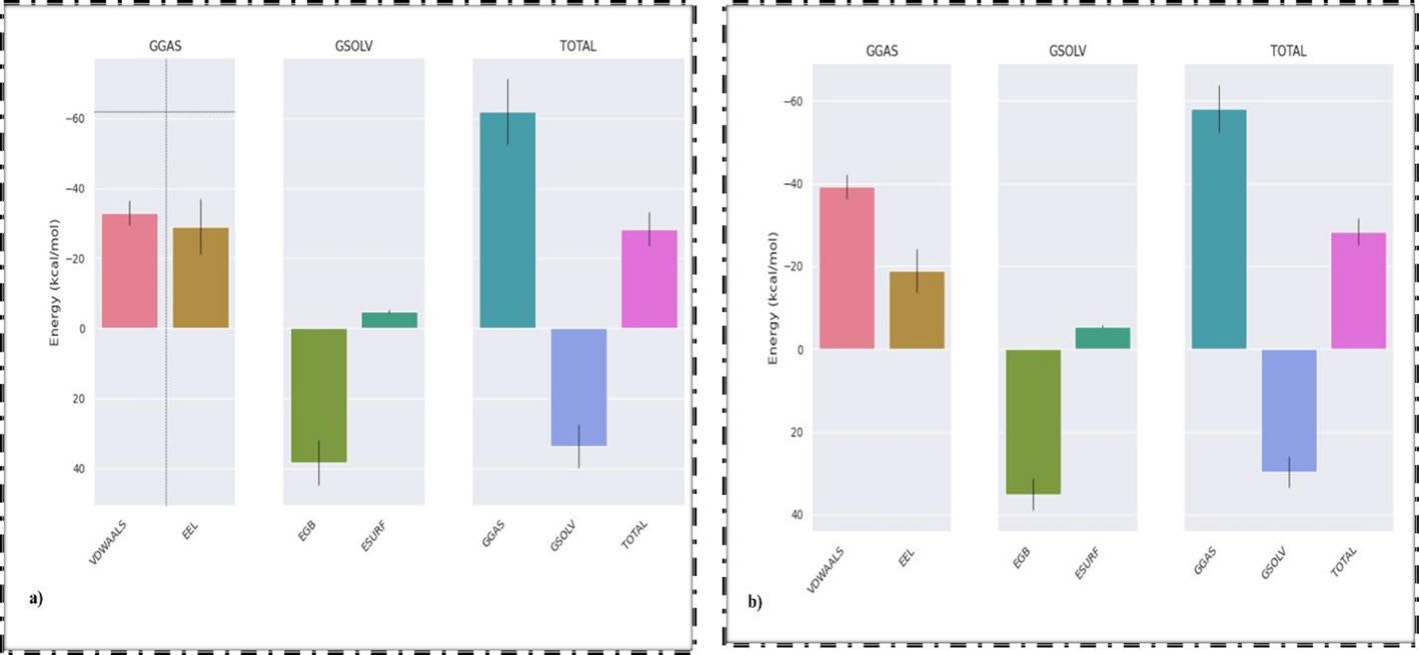
Total binding free energy interaction profile of (**a**) HDAC1-VOR and (**b**) HDAC1-HIS complexes from MM-PBSA analysis

The binding free energy for the HDAC1-VOR, HDAC1-HIS, and HDAC1-PEL complexes were calculated using the g_mmbsa tool and gmx_MMPBSA_ana visualization tool of GROMACS. The MMPBSA analysis was performed for the whole 100 ns simulation of the complex. The bar plot depicts the ΔG_gas_ (sum of ΔE_vdw_ and Δ E_elec_) and ΔG_solvation_ (ΔE_GB_– Effective Generalised Born energy) along with the ΔG_total_ (sum of ΔG_gas_ and ΔG_solvation_) (Fig. 4a and b). The complexes exhibited low total binding free energy (ΔG_total_), for HDAC-VOR: −28.39 ± 4.81 kcal/mol, HDAC1-HIS: −28.32 ± 3.23 kcal/mol, and HDAC1-PEL: −9.41 ± 4.52 kcal/mol. The MMPSA analysis gave the following energy values ΔE_vdw_, ΔE_elec_, ΔG_solvation_, Gas-phase Gibbs free energy ΔG_gas_, and ΔE_surf_ for all the three complexes that are tabulated in Table 5.

**Table 5:** MM-PBSA analysis of protein-ligand complexes

Compounds	Energy (kcal/mol)						
	ΔE_vdw_	ΔE_elec_	ΔE_GB_	ΔE_surf_	ΔG_gas_	ΔG_solvation_	ΔG_total_
**Vorinostat**	−32.94 ± 3.44	−29.12 ± 7.93	38.42 ± 6.47	−4.75 ± 0.47	−62.06 ± 9.4	33.67 ± 6.22	−28.39 ± 4.81
**Hispidulin**	−39.16 ± 2.93	−18.92 ± 5.22	35.14 ± 3.87	−5.39 ± 0.38	−58.07 ± 5.75	29.75 ± 3.75	−28.32 ± 3.23
**Pelargonidin**	−16.25 ± 5.99	−10.07 ± 7.82	19.25 ± 7.72	−2.34 ± 0.86	−26.32 ± 10.42	16.9 ± 7.2	−9.41 ± 4.52

## Discussion

The epigenetic importance of HDAC1 in hematopoiesis has made it a prominent target in controlling AML activity. The HDAC1 controls the activity of transcription factors by monitoring the accessibility to chromatin of the target gene and epigenetic modifiers involved in cellular proliferation and carcinogenesis [61, 62]. In this study, anti-leukemic phytochemicals retrieval and screening was performed to find a potential lead candidate against HDAC1 protein. The currently available drug Panobinostat was proven to be resistant in AML cells when there is low expression of *GF1* gene. The aim is to find out a potential drug candidate as an alternative for Panobinostat against HDAC1 which could be achieved by phytochemicals. Phytocompounds can reverse drug resistance by affecting the expression of ATP-Binding Cassette (ABC) and also by acting synergistically together with other anti-cancer drugs against cancer cells increasing the efficiency of the treatment [63].

For a potential drug candidate to be considered for administration, it must have optimal phramcokinetic properties and be non-toxic to the body. Hence, ADME screening was performed for filtering phytochemicals based on their effect on the body [64]. The GI absorption of the drug for oral administration must be high to facilitate transport to parts of the body [65,66]. Lipinski’s rule of five helps to define the drugability of a molecule based on the molecular mass, partition co-efficient, hydrogen bond donors, and acceptors [67]. Lead-likeness property of a compound is a significant guide for selecting out a potential drug-like candidate for drug discovery. There must be zero violations for molecules to be screened as a potential drug candidate [68]. The TPSA is a molecular descriptor used to analyze molecular transport based on the surface area of polar atoms and their attached hydrogen atoms. A TPSA value between 60 and 140 was taken as a threshold value in this study which indicates the optimum surface area for better molecular transport [69]. The SA score is based on the fragment contribution and complexity penalty in account to the presence of non-stranded structures in the molecule. The SA score is a scale from 1 to 10, one being very easy to 10 being very difficult [70]. Based on the aforementioned parameters, 24 lead compounds with active pharmacokinetic properties were screened [71].

The toxicity prediction of molecules could be assessed by measuring the toxicity endpoints such as cytotoxicity, carcinogenicity, immunotoxicity, mutagenicity and organ toxicity parameters such as hepatotoxicity for evaluating a non-toxic drug candidate [72]. A study investigating the hepatotoxicity, carcinogenicity, mutagenicity, cytotoxicity, and immunotoxicity was conducted using the ProTox web server alongside in vitro assays and different benchmark *insilico* studies. The findings demonstrated an accuracy of 82% in hepatotoxicity, 81.24% in carcinogenecity, 84% in mutagenicity, 85% in cytotoxicity, and 74% in immunotoxicity respectively, indicating that the predictions generated by the ProTox web server are reliable for toxicity assessment [73–78]. The five compounds that were filtered out which were inactive in the toxicity parameters mentioned above filtered: hispidulin, pelargonidin, kumatakenin, kaempferol, rohitukine and phenyl glycoside. This toxicity prediction is based on the probability prediction score. For all the compounds it was found that the inactive probability is more than 0.50 for all the toxicity parameters considered indicating that the screened compounds are promising lead candidates [79].

The molecular docking analysis was performed for the screened ADME and non-toxic compounds along with PC and NC to study the protein-ligand interactions. The binding affinity and Ki derived from docking analysis were considered as a function of efficient docking interaction between protein and ligand [80]. The docking analysis focused on targeting the residues that interact with the zinc atom, rather than the zinc atom itself, as these residues may play a critical role in influencing the HDAC1 function [81]. Targeting these key residues could significantly impact the protein’s activity. Overall, four compounds were showing interactions with the active site residues. The docking analysis was well validated using PC vorinostat and NC, a low affinity or a ligand that should not ideally bind to the protein such as glycerol was used [46]. The results indicated efficient docking of the ligand to the protein as the NC showed very low binding affinity and the PC exhibited high binding affinity with HDAC1. Every compound except the NC showed relatively good binding affinity among which hispidulin showed better binding affinity and low Ki than PC. When compared with PC, hispidulin had the closest binding affinity making it significant followed by pelargonidin. The HDAC1 has a deep catalytic cavity above the zinc atom at the tail of the cavity. The residues ASP176, HIS178, ASP264, and ASP99 play pivotal role in ligand binding because the first three residues directly interacting with the zinc atom which is significant for substrate binding as HDAC1 is a zinc dependent histone deacetylase [17, 82]. The later residue ASP99 plays a vital role in anchoring the substrate. This ASP99 is conserved in all HDAC proteins whose side chain rearrangement is significant for peptide backbone binding of the substrate. Hence, ASP99 which is at the start of the catalytic cavity is important for optimum positioning of the ligand [83]. All the compounds were majorly interacting with the active site residues ASP99, HIS178, and ASP176. Notably, hispidulin, pelargonidin and PC are interacting with HIS178 and ASP176 residues which were bound to zinc atom with better binding affinity making the ligands significant impact on HDAC1 interactions with other proteins [43]. This indicates efficient molecular docking of the compounds and the ligand interactions with zinc bound residues could have significant impact on the activity of the protein. Hispidulin and pelargonidin showed better binding affinity than all other compounds with the low Ki. Its binding site is comprised of all the pivotal residues in its interaction profile supporting docking analysis significantly. Due to hispidulin’s relatively smaller molecular size compared to vorinostat, hispidulin does not interact with the surface residue. In contrast, vorinostat, with its larger molecular size, engages with residues at the protein surface. This structural difference enables hispidulin to achieve deeper binding within the catalytic pocket, facilitating more effective interactions. The low Ki value of hispidulin (1.91 µM) suggests its superior binding and enhanced inhibition function against the protein which is reflected by its higher binding affinity than vorinostat. This Ki value is considered reliable, as AutoDock’s Ki predictions demonstrate strong agreement with experimental results, indicating high accuracy [10].

The structural stability of the HDAC1-VOR, HDAC1-HIS, and HDAC1-PEL complexes were evaluated by MD simulation. Based on RMSD analysis, the HDAC1-HIS complex has produced a lower RMSD value (0.7 ± 0.2 nm) than the PC complex. This shows that the HDAC1-HIS complex was stable throughout the 100 ns simulation depicting the binding strength of hispidulin is efficient than vorinostat and pelargonidin. The RMSF analysis depicted very less fluctuations of atoms ranging from 0.1 nm to 0.75 ± 0.1 nm with a maximum fluctuation of 0.7 ± 0.1 nm at LEU271 which is a coiled residue. The presence of multiple coiled coils in a protein structure increases conformational flexibility, which may account for the observed fluctuation at LEU271 [84]. Overall, the RMSF analysis showed low fluctuations indicating the atom stability of both the HDAC1 and ligands. On the other hand, the complexes displayed similar Rg range (2 nm to 2.1 nm) with a deviation of 2 ± 0.1 nm. This lower deviation in the Rg value suggests that the protein structure is more compact indicating the ligands bound to the protein has not affected the compactness of HDAC1 [85,86]. The MD simulation recorded the formation of a H-bond in HDAC1-HIS and two H-bonds in HDAC1-VOR but no H-Bond is formed in HDAC1-PEL complex at the end of 100 ns between HDAC1-HIS. Hydrogen bonding plays a critical role in stabilizing protein-ligand complexes. Both HDAC1-VOR and HDAC1-HIS complexes exhibited stable interactions throughout the simulation, primarily due to the presence of these bonds [87].The sasa analysis aided in concluding the protein stability with respect to ligand interaction. The analysis depicted that HDAC1-HIS has a higher sasa compared to the other complexes indicating that it has slightly different binding characteristics whereas HDAC1-VOR and HDAC1-PEL has similar binding profiles as their averages are equal. Anyways, the stable fluctuations over time suggests that protein structure remains consistent without significant structural changes [88]. In addition, the E_int_ of −208.42 kJ/mol in HDAC1-HIS and − 202.04 kJ/mol in HDAC1-VOR has suggested that non-covalent interactions like van der Waals interactions and electrostatic interactions have also played a significant role in ligand binding because of lower interaction energy [89]. The E_int_ of HDAC1-HIS is comparatively lower than the other complexes evidently proving that hispidulin has stronger non-bonded interactions with HDAC1 than the PC vorinostat. The interaction of vorinostat with surface residues during docking analysis results in its exposure to solvent molecules during molecular dynamics simulations (MDS), potentially affecting its stability that is reflected in RMSD graph. In contrast, hispidulin binds deeply within the active site cavity of the HDAC1 protein, leading to enhanced stability. This deeper binding minimizes solvent exposure, potentially improving ligand retention and reducing conformational fluctuations. Consequently, hispidulin may exhibit a more favorable binding profile, contributing to its increased inhibitory potential against HDAC1.

The total binding free energy of the complex supported the optimum binding of hispidulin to HDAC1 with ΔG_total_ = −28.32 ± 3.23 kcal/mol which is similar to PC vorinostat to HDAC1 with ΔG_total_ = −28.39 ± 4.81 kcal/mol. Here, in both the cases electrostatic interactions and van de Waals interactions played a major role in ligand binding with ΔG_gas_ of −58.07 ± 5.75 kcal/mol for HDAC1-HIS and − 62.06 ± 9.4 kcal/mol for HDAC1-VOR. This analysis showed that the interaction energy between HDAC1-PEL was insignificant and the ΔG_total_ was comparatively lower than hispidulin. From the energy and MMPBSA analysis it is evident that the non-bonded interactions have well supported for the ligand interaction with HDAC1. The low total binding free energy obtained here strongly supports the efficient binding of hispidulin to HDAC1 [90]. The analysis indicates that hispidulin has exhibited an optimum ΔG value and has formed a stable complex with the protein with respect to vorinostat [91].

*Tithonia diversifolia* also known as Mexican sunflower is a shrub-like perennial plant that was used for the treatment of diseases like malaria, diabetes, gastro-intestinal diseases, hepatitis, and various other conditions by many ethnic groups [92]. The extracts of the plant have shown potential pharmacological activity against AML cell lines by promoting apoptosis and arresting the cell cycle at the G_0_/G_1_ phase [93]. Hispidulin is one of the compounds present in *Tithonia diversifolia* which was retrieved from IMPPAT database. It exhibits antifungal, antioxidant, and anti-inflammatory properties, and also has potential anti-cancer effects affecting the cell proliferation. Moreover, hispidulin also exerts a synergistic action with other common anti-cancer drugs which is said to reduce chemo-sensitivity and reverse drug resistance [94]. A hispidulin-rich plant (sesewanua) was investigated for its potential antithrombotic activity, with its efficacy evaluated through the development of microneedle-based formulations. The study demonstrated the feasibility of utilizing microneedles for thrombosis treatment, highlighting their potential for controlled and localized drug delivery [95]. Hispidulin has also been reported to exhibit potent cytotoxic effects against hepatocellular carcinoma (HCC) by inhibiting the expression of matrix metalloproteinases. Additionally, it has been shown to suppress cell growth, proliferation, and metastasis of HCC cells through the activation of peroxisome proliferator-activated receptor gamma (PPARγ) in both in vitro and in vivo studies [96, 97]. Hispidulin demonstrates potential clinical applicability, as previous studies have shown that microneedle formulations can enhance its efficacy, thereby improving clinical outcomes. An in-vitro study evaluating the effects of hispidulin on a human leukemia cell line demonstrated that hispidulin suppresses AML cell proliferation in a dose- and time-dependent manner and induces apoptosis through an intrinsic mitochondrial pathway. Additionally, hispidulin significantly downregulates extracellular matrix metalloproteinase inducer (EMMPRIN) expression in AML cell lines, thereby inhibiting the AKT and STAT3 signaling pathways [98]. However, challenges may arise in optimizing the formulation to align with the physiological characteristics of acute myeloid leukemia (AML) cells. Nevertheless, based on our results in the present study and prior findings from experimental observations, hispidulin holds promise for therapeutic intervention in AML, warranting further investigation. Hispidulin has the potential to serve as an anti-leukemic agent targeting HDAC1, offering a novel approach to overcoming resistance associated with vorinostat. Based on previous studies, hispidulin may modulate distinct molecular pathways compared to vorinostat, thereby providing an alternative therapeutic strategy for AML treatment.

From the above in-silico analysis of the phytochemical compound hispidulin was identified as a potential lead candidate against HDAC1 for epigenetically reducing the AML prognosis. Hispidulin showed better characteristics in terms of ADME properties, toxicity, molecular docking profile, and stability in MD simulation with comparison with vorinostat. Thus, from this study, hispidulin turned out to be a prominent anti-leukemic lead candidate among other phytochemicals analyzed against HDAC1 and might have the ability to overcome vorinostat resistance against HDAC.

## Conclusion

Hispidulin a natural compound was found to have excellent binding with HDAC1, an epigenetic regulator whose role is significant in AML. The currently available drug against HDAC1, vorinostat showed significant resistance thus phytochemicals are suggested as potential alternatives. Based on the phytochemical ADMET screening hispidulin, rohitukine, kumatakenin, phenyl glucoside, and kaempferol were identified to be potential lead compounds. Among them, hispidulin showed promising molecular docking results with highest binding affinity with HDAC1. The molecular simulation analysis comprising RMSD, RMSF, Rg, H-bond analysis, IE, and MM-PBSA calculation supported the stability of the HDAC1-HIS complex. The binding of hispidulin to HDAC1 may play a crucial role in overcoming drug resistance and modulating the prognosis of AML. This study establishes a robust foundation for further experimental validation through AML cell line inhibition assays and in vivo animal model testing, facilitating the potential development of hispidulin as a therapeutic agent for AML.

## Electronic supplementary material

Supplementary Material 1

## Data Availability

All data generated during the study are included in this article.
