# Supplementary material for: Hispidulin: a potential alternative to vorinostat against HDAC1 for acute myeloid leukemia
Source: Discov Oncol. 2025 Jul 22;16:1389. doi: 10.1007/s12672-025-03182-y (PMC12283524; doi:10.1007/s12672-025-03182-y)
Supplement: Supplementary file 1 — Supplementary Material 1 [file 12672_2025_3182_MOESM1_ESM.docx]

**Supplementary (Table S1):** List of ant-leukemic phytochemical compounds retrieved.

| **S.no** | **Compound name** | **SMILES** | **Pubchem ID** |
| --- | --- | --- | --- |
| 1 | [3-(4-Hydroxyphenyl)propionic acid](https://cb.imsc.res.in/imppat/phytochemical-detailedpage/IMPHY001087) | OC(=O)CCc1ccc(cc1)O | [10394](https://pubchem.ncbi.nlm.nih.gov/compound/10394) |
| 2 | [Coumarin](https://cb.imsc.res.in/imppat/phytochemical-detailedpage/IMPHY003490) | O=c1ccc2c(o1)cccc2 | [323](https://pubchem.ncbi.nlm.nih.gov/compound/323) |
| 3 | [Betacyanin](https://cb.imsc.res.in/imppat/phytochemical-detailedpage/IMPHY004274) | OC[C@H]1O[C@@H](Oc2cc3C[C@H](/[N+](=CC=C/4C[C@H](NC(=C4)C(=O)O)C(=O)O)/c3cc2O)C(=O)[O-])[C@@H]([C@H]([C@@H]1O)O)O | [6324775](https://pubchem.ncbi.nlm.nih.gov/compound/6324775) |
| 4 | [Quercetin](https://cb.imsc.res.in/imppat/phytochemical-detailedpage/IMPHY004619) | Oc1cc(O)c2c(c1)oc(c(c2=O)O)c1ccc(c(c1)O)O | [5280343](https://pubchem.ncbi.nlm.nih.gov/compound/5280343) |
| 5 | [3-(2-Hydroxyphenyl)propanoic acid](https://cb.imsc.res.in/imppat/phytochemical-detailedpage/IMPHY006935) | OC(=O)CCc1ccccc1O | [873](https://pubchem.ncbi.nlm.nih.gov/compound/873) |
| 6 | [Vanillic acid](https://cb.imsc.res.in/imppat/phytochemical-detailedpage/IMPHY007084) | COc1cc(ccc1O)C(=O)O | [8468](https://pubchem.ncbi.nlm.nih.gov/compound/8468) |
| 7 | [Salicylic acid](https://cb.imsc.res.in/imppat/phytochemical-detailedpage/IMPHY011523) | OC(=O)c1ccccc1O | [338](https://pubchem.ncbi.nlm.nih.gov/compound/338) |
| 8 | [Scopoletin](https://cb.imsc.res.in/imppat/phytochemical-detailedpage/IMPHY011541) | COc1cc2ccc(=O)oc2cc1O | [5280460](https://pubchem.ncbi.nlm.nih.gov/compound/5280460) |
| 9 | [Chlorogenic acid](https://cb.imsc.res.in/imppat/phytochemical-detailedpage/IMPHY011844) | O=C(O[C@@H]1C[C@@](O)(C[C@H]([C@H]1O)O)C(=O)O)/C=C/c1ccc(c(c1)O)O | [1794427](https://pubchem.ncbi.nlm.nih.gov/compound/1794427) |
| 10 | [Caffeic acid](https://cb.imsc.res.in/imppat/phytochemical-detailedpage/IMPHY011933) | OC(=O)/C=C/c1ccc(c(c1)O)O | [689043](https://pubchem.ncbi.nlm.nih.gov/compound/689043) |
| 11 | [Cinnamic acid](https://cb.imsc.res.in/imppat/phytochemical-detailedpage/IMPHY011960) | OC(=O)/C=C/c1ccccc1 | [444539](https://pubchem.ncbi.nlm.nih.gov/compound/444539) |
| 12 | [cis-p-Coumaric acid](https://cb.imsc.res.in/imppat/phytochemical-detailedpage/IMPHY011972) | OC(=O)/C=Cc1ccc(cc1)O | [1549106](https://pubchem.ncbi.nlm.nih.gov/compound/1549106) |
| 13 | [4-Hydroxycinnamic acid](https://cb.imsc.res.in/imppat/phytochemical-detailedpage/IMPHY011974) | OC(=O)/C=C/c1ccc(cc1)O | [637542](https://pubchem.ncbi.nlm.nih.gov/compound/637542) |
| 14 | [Rutin](https://cb.imsc.res.in/imppat/phytochemical-detailedpage/IMPHY015047) | Oc1cc(O)c2c(c1)oc(c(c2=O)O[C@@H]1O[C@H](CO[C@@H]2O[C@@H](C)[C@@H]([C@H]([C@H]2O)O)O)[C@H]([C@@H]([C@H]1O)O)O)c1ccc(c(c1)O)O | [5280805](https://pubchem.ncbi.nlm.nih.gov/compound/5280805) |
| 15 | [Isogomphrenin II](https://cb.imsc.res.in/imppat/phytochemical-detailedpage/IMPHY000412) | O=C(/C=C/c1ccc(cc1)O)OC[C@H]1O[C@@H](Oc2cc3c(cc2O)C[C@@H](N3/C=C/C2=CC(=N[C@@H](C2)C(=O)O)C(=O)O)C(=O)O)[C@@H]([C@H]([C@@H]1O)O)O | [101105497](https://pubchem.ncbi.nlm.nih.gov/compound/101105497) |
| 16 | [Retinol](https://cb.imsc.res.in/imppat/phytochemical-detailedpage/IMPHY001308) | OC/C=C(/C=C/C=C(/C=C/C1=C(C)CCCC1(C)C)C)C | [445354](https://pubchem.ncbi.nlm.nih.gov/compound/445354) |
| 17 | [1'-OH-gamma-carotene glucoside/(Carotenoids B-G)](https://cb.imsc.res.in/imppat/phytochemical-detailedpage/IMPHY002286) | OC[C@H]1O[C@@H](OC(CCC/C(=C/C=C/C(=C/C=C/C(=C/C=C/C=C(/C=C/C=C(/C=C/C2=C(C)CCCC2(C)C)C)C)/C)/C)/C)(C)C)C(C([C@@H]1O)O)O | [16061280](https://pubchem.ncbi.nlm.nih.gov/compound/16061280) |
| 18 | [Lutein](https://cb.imsc.res.in/imppat/phytochemical-detailedpage/IMPHY011620) | C/C(=CC=CC=C(C=CC=C(C=CC1=C(C)C[C@H](CC1(C)C)O)/C)/C)/C=C/C=C(/C=C/[C@H]1C(=C[C@@H](CC1(C)C)O)C)C | [5281243](https://pubchem.ncbi.nlm.nih.gov/compound/5281243) |
| 19 | [Zeaxanthin](https://cb.imsc.res.in/imppat/phytochemical-detailedpage/IMPHY011785) | O[C@@H]1CC(=C(C(C1)(C)C)/C=C/C(=C/C=C/C(=C/C=C/C=C(/C=C/C=C(/C=C/C1=C(C)C[C@H](CC1(C)C)O)C)C)/C)/C)C | [5280899](https://pubchem.ncbi.nlm.nih.gov/compound/5280899) |
| 20 | [Glycoprotein](https://cb.imsc.res.in/imppat/phytochemical-detailedpage/IMPHY012513) | OC[C@H]1OC(NC(=O)C[C@@H](C(=O)N)NC(=O)C)[C@@H]([C@H]([C@@H]1O[C@@H]1O[C@H](CO)[C@H]([C@@H]([C@H]1NC(=O)C)O)O[C@@H]1O[C@H](CO)[C@H]([C@@H]([C@@H]1O)O)O)O)NC(=O)C | [439212](https://pubchem.ncbi.nlm.nih.gov/compound/439212) |
| 21 | [Thiamine](https://cb.imsc.res.in/imppat/phytochemical-detailedpage/IMPHY000005) | OCCc1sc[n+](c1C)Cc1cnc(nc1N)C | [1130](https://pubchem.ncbi.nlm.nih.gov/compound/1130) |
| 22 | [Riboflavin](https://cb.imsc.res.in/imppat/phytochemical-detailedpage/IMPHY000846) | OC[C@H]([C@H]([C@H](Cn1c2-c(nc3c1cc(C)c(c3)C)c(=O)[nH]c(=O)n2)O)O)O | [493570](https://pubchem.ncbi.nlm.nih.gov/compound/493570) |
| 23 | [Arachidonic acid](https://cb.imsc.res.in/imppat/phytochemical-detailedpage/IMPHY004302) | CCCCC/C=CC/C=CC/C=CC/C=CCCCC(=O)O | [444899](https://pubchem.ncbi.nlm.nih.gov/compound/444899) |
| 24 | [Palmitic acid](https://cb.imsc.res.in/imppat/phytochemical-detailedpage/IMPHY007327) | CCCCCCCCCCCCCCCC(=O)O | [985](https://pubchem.ncbi.nlm.nih.gov/compound/985) |
| 25 | [Nicotinic acid](https://cb.imsc.res.in/imppat/phytochemical-detailedpage/IMPHY007357) | OC(=O)c1cccnc1 | [938](cid:938) |
| 26 | [alpha-Carotene](https://cb.imsc.res.in/imppat/phytochemical-detailedpage/IMPHY011609) | C/C(=CC=CC=C(C=CC=C(C=CC1=C(C)CCCC1(C)C)/C)/C)/C=C/C=C(/C=C/[C@H]1C(=CCCC1(C)C)C)C | [6419725](https://pubchem.ncbi.nlm.nih.gov/compound/6419725) |
| 27 | [Oleic acid](https://cb.imsc.res.in/imppat/phytochemical-detailedpage/IMPHY011797) | CCCCCCCC/C=CCCCCCCCC(=O)O | [445639](https://pubchem.ncbi.nlm.nih.gov/compound/445639) |
| 28 | [Linolenic acid](https://cb.imsc.res.in/imppat/phytochemical-detailedpage/IMPHY012723) | CC/C=CC/C=CC/C=CCCCCCCCC(=O)O | [5280934](cid:5280934) |
| 29 | [Linoleic acid](https://cb.imsc.res.in/imppat/phytochemical-detailedpage/IMPHY014990) | CCCCC/C=CC/C=CCCCCCCCC(=O)O | [5280450](https://pubchem.ncbi.nlm.nih.gov/compound/5280450) |
| 30 | [Gomphrenin III](https://cb.imsc.res.in/imppat/phytochemical-detailedpage/IMPHY000397) | COc1cc(/C=C/C(=O)OC[C@H]2O[C@@H](Oc3cc4c(cc3O)C[C@H](N4/C=C/C3=CC(=N[C@@H](C3)C(=O)O)C(=O)O)C(=O)O)[C@@H]([C@H]([C@@H]2O)O)O)ccc1O | [101105498](https://pubchem.ncbi.nlm.nih.gov/compound/101105498) |
| 31 | [Ascorbic acid](https://cb.imsc.res.in/imppat/phytochemical-detailedpage/IMPHY006362) | OC[C@@H]([C@H]1OC(=O)C(=C1O)O)O | [54670067](https://pubchem.ncbi.nlm.nih.gov/compound/54670067) |
| 32 | [Gomphrenin II](https://cb.imsc.res.in/imppat/phytochemical-detailedpage/IMPHY008844) | C1C(NC(=CC1=CC=[N+]2C(CC3=CC(=C(C=C32)OC4C(C(C(C(O4)COC(=O)C=CC5=CC=C(C=C5)O)O)O)O)O)C(=O)[O-])C(=O)O)C(=O)O | 131752748 |
| 33 | [beta-Carotene](https://cb.imsc.res.in/imppat/phytochemical-detailedpage/IMPHY011707) | C/C(=CC=CC=C(C=CC=C(C=CC1=C(C)CCCC1(C)C)/C)/C)/C=C/C=C(/C=C/C1=C(C)CCCC1(C)C)C | [5280489](https://pubchem.ncbi.nlm.nih.gov/compound/5280489) |
| 34 | [Ferulic acid](https://cb.imsc.res.in/imppat/phytochemical-detailedpage/IMPHY011802) | COc1cc(/C=C/C(=O)O)ccc1O | [445858](https://pubchem.ncbi.nlm.nih.gov/compound/445858) |
| 35 | [Gomphrenin I](https://cb.imsc.res.in/imppat/phytochemical-detailedpage/IMPHY014928) | OC[C@H]1O[C@@H](Oc2cc3c(cc2O)C[C@H](N3/C=C/C2=CC(=N[C@@H](C2)C(=O)O)C(=O)O)C(=O)O)[C@@H]([C@H]([C@@H]1O)O)O | [90658633](https://pubchem.ncbi.nlm.nih.gov/compound/90658633) |
| 36 | [arbortristoside C](https://cb.imsc.res.in/imppat/phytochemical-detailedpage/IMPHY002936) | OC[C@H]1O[C@@H](O[C@@H]2OC=C([C@@H]3[C@H]2[C@@H](C)[C@H]([C@H]3O)OC(=O)/C=C/c2ccc(cc2)O)C(=O)OC)[C@@H]([C@H]([C@@H]1O)O)O | [23955893](https://pubchem.ncbi.nlm.nih.gov/compound/23955893) |
| 37 | [Methyl salicylate](https://cb.imsc.res.in/imppat/phytochemical-detailedpage/IMPHY003050) | COC(=O)c1ccccc1O | [4133](https://pubchem.ncbi.nlm.nih.gov/compound/4133) |
| 38 | [4-Methoxybenzaldehyde](https://cb.imsc.res.in/imppat/phytochemical-detailedpage/IMPHY003482) | COc1ccc(cc1)C=O | [31244](https://pubchem.ncbi.nlm.nih.gov/compound/31244) |
| 39 | [beta-D-gentiobiosyl crocetin](https://cb.imsc.res.in/imppat/phytochemical-detailedpage/IMPHY003940) | OC[C@H]1O[C@@H](OC[C@H]2O[C@@H](OC(=O)/C(=C/C=C/C(=C/C=C/C=C(/C=C/C=C(/C(=O)O)C)C)/C)/C)[C@@H]([C@H]([C@@H]2O)O)O)[C@@H]([C@H]([C@@H]1O)O)O | [10461942](https://pubchem.ncbi.nlm.nih.gov/compound/10461942) |
| 40 | [Crocetin gentiobiosylglucosyl ester](https://cb.imsc.res.in/imppat/phytochemical-detailedpage/IMPHY004098) | OC[C@H]1O[C@@H](OC[C@H]2O[C@@H](OC(=O)/C(=C/C=C/C(=C/C=C/C=C(/C=C/C=C(/C(=O)O[C@@H]3O[C@H](CO)[C@H]([C@@H]([C@H]3O)O)O)C)C)/C)/C)[C@@H]([C@H]([C@@H]2O)O)O)[C@@H]([C@H]([C@@H]1O)O)O | [9940690](https://pubchem.ncbi.nlm.nih.gov/compound/9940690) |
| 41 | [p-Cymene](https://cb.imsc.res.in/imppat/phytochemical-detailedpage/IMPHY006145) | Cc1ccc(cc1)C(C)C | [7463](https://pubchem.ncbi.nlm.nih.gov/compound/7463) |
| 42 | [Methyl palmitate](https://cb.imsc.res.in/imppat/phytochemical-detailedpage/IMPHY006971) | CCCCCCCCCCCCCCCC(=O)OC | [8181](https://pubchem.ncbi.nlm.nih.gov/compound/8181) |
| 43 | [1-Hexanol](https://cb.imsc.res.in/imppat/phytochemical-detailedpage/IMPHY007171) | CCCCCCO | [8103](https://pubchem.ncbi.nlm.nih.gov/compound/8103) |
| 44 | [6-Methyl-5-hepten-2-one](https://cb.imsc.res.in/imppat/phytochemical-detailedpage/IMPHY007331) | CC(=O)CCC=C(C)C | [9862](https://pubchem.ncbi.nlm.nih.gov/compound/9862) |
| 45 | [Phenylacetaldehyde](https://cb.imsc.res.in/imppat/phytochemical-detailedpage/IMPHY007539) | O=CCc1ccccc1 | [998](https://pubchem.ncbi.nlm.nih.gov/compound/998) |
| 46 | [Nyctanthoside](https://cb.imsc.res.in/imppat/phytochemical-detailedpage/IMPHY007589) | OC[C@H]1[C@@H](O)[C@H]([C@H]2[C@@H]1[C@@H](OC=C2C(=O)OC)O[C@@H]1O[C@H](CO)[C@H]([C@@H]([C@H]1O)O)O)O | [95224501](https://pubchem.ncbi.nlm.nih.gov/compound/95224501) |
| 47 | [Hentriacontane](https://cb.imsc.res.in/imppat/phytochemical-detailedpage/IMPHY008910) | CCCCCCCCCCCCCCCCCCCCCCCCCCCCCCC | [12410](https://pubchem.ncbi.nlm.nih.gov/compound/12410) |
| 48 | [Crocetin](https://cb.imsc.res.in/imppat/phytochemical-detailedpage/IMPHY010351) | C/C(=CC=CC=C(C=CC=C(C(=O)O)/C)/C)/C=C/C=C(/C(=O)O)C | [5281232](https://pubchem.ncbi.nlm.nih.gov/compound/5281232) |
| 49 | [Mannitol](https://cb.imsc.res.in/imppat/phytochemical-detailedpage/IMPHY011729) | OC[C@H]([C@H]([C@@H]([C@@H](CO)O)O)O)O | [6251](https://pubchem.ncbi.nlm.nih.gov/compound/6251) |
| 50 | [Tannic acid](https://cb.imsc.res.in/imppat/phytochemical-detailedpage/IMPHY011741) | O=C(c1cc(O)c(c(c1)OC(=O)c1cc(O)c(c(c1)O)O)O)O[C@@H]1[C@@H](COC(=O)c2cc(O)c(c(c2)OC(=O)c2cc(O)c(c(c2)O)O)O)O[C@H]([C@@H]([C@H]1OC(=O)c1cc(O)c(c(c1)OC(=O)c1cc(O)c(c(c1)O)O)O)OC(=O)c1cc(O)c(c(c1)OC(=O)c1cc(O)c(c(c1)O)O)O)OC(=O)c1cc(O)c(c(c1)OC(=O)c1cc(O)c(c(c1)O)O)O | [16129778](https://pubchem.ncbi.nlm.nih.gov/compound/16129778) |
| 51 | [Crocin](https://cb.imsc.res.in/imppat/phytochemical-detailedpage/IMPHY011824) | OC[C@H]1O[C@@H](OC[C@H]2O[C@@H](OC(=O)/C(=C/C=C/C(=C/C=C/C=C(/C=C/C=C(/C(=O)O[C@@H]3O[C@H](CO[C@@H]4O[C@H](CO)[C@H]([C@@H]([C@H]4O)O)O)[C@H]([C@@H]([C@H]3O)O)O)C)C)/C)/C)[C@@H]([C@H]([C@@H]2O)O)O)[C@@H]([C@H]([C@@H]1O)O)O | [5281233](https://pubchem.ncbi.nlm.nih.gov/compound/5281233) |
| 52 | [Nicotiflorin](https://cb.imsc.res.in/imppat/phytochemical-detailedpage/IMPHY011985) | Oc1ccc(cc1)c1oc2cc(O)cc(c2c(=O)c1O[C@@H]1O[C@H](CO[C@@H]2O[C@@H](C)[C@@H]([C@H]([C@H]2O)O)O)[C@H]([C@@H]([C@H]1O)O)O)O | [5318767](https://pubchem.ncbi.nlm.nih.gov/compound/5318767) |
| 53 | [alpha-Pinene](https://cb.imsc.res.in/imppat/phytochemical-detailedpage/IMPHY012061) | CC1=CCC2CC1C2(C)C | [6654](https://pubchem.ncbi.nlm.nih.gov/compound/6654) |
| 54 | [beta-Amyrin](https://cb.imsc.res.in/imppat/phytochemical-detailedpage/IMPHY012223) | O[C@H]1CC[C@]2([C@H](C1(C)C)CC[C@@]1([C@@H]2CC=C2[C@@]1(C)CC[C@@]1([C@H]2CC(C)(C)CC1)C)C)C | [73145](https://pubchem.ncbi.nlm.nih.gov/compound/73145) |
| 55 | [Lupeol](https://cb.imsc.res.in/imppat/phytochemical-detailedpage/IMPHY012473) | CC(=C)[C@@H]1CC[C@]2([C@H]1[C@H]1CC[C@H]3[C@@]([C@]1(C)CC2)(C)CC[C@@H]1[C@]3(C)CC[C@@H](C1(C)C)O)C | [259846](https://pubchem.ncbi.nlm.nih.gov/compound/259846) |
| 56 | [Arbortristoside B](https://cb.imsc.res.in/imppat/phytochemical-detailedpage/IMPHY012931) | OCC1C(O)C([C@H]2C1[C@@H](OC=C2C(=O)OC)O[C@@H]1O[C@H](CO)[C@H]([C@@H]([C@H]1O)O)O)OC(=O)/C=C/c1ccc(c(c1)O)O | [6442163](https://pubchem.ncbi.nlm.nih.gov/compound/6442163) |
| 57 | [Astragalin](https://cb.imsc.res.in/imppat/phytochemical-detailedpage/IMPHY014824) | OC[C@H]1O[C@@H](Oc2c(oc3c(c2=O)c(O)cc(c3)O)c2ccc(cc2)O)[C@@H]([C@H]([C@@H]1O)O)O | [5282102](https://pubchem.ncbi.nlm.nih.gov/compound/5282102) |
| 58 | [2,6,10-Trimethylpentadecane](https://cb.imsc.res.in/imppat/phytochemical-detailedpage/IMPHY015240) | CCCCCC(CCCC(CCCC(C)C)C)C | [19775](https://pubchem.ncbi.nlm.nih.gov/compound/19775) |
| 59 | [2-Methyldocosane](https://cb.imsc.res.in/imppat/phytochemical-detailedpage/IMPHY015279) | CCCCCCCCCCCCCCCCCCCCC(C)C | [519145](https://pubchem.ncbi.nlm.nih.gov/compound/519145) |
| 60 | [beta-Methylionone](https://cb.imsc.res.in/imppat/phytochemical-detailedpage/IMPHY017437) | CCC(=O)/C=C/C1=C(C)CCCC1(C)C | [5375218](https://pubchem.ncbi.nlm.nih.gov/compound/5375218) |
| 61 | [Curlone](https://cb.imsc.res.in/imppat/phytochemical-detailedpage/IMPHY005798) | CC(C1CCC(=C)C=C1)CC(=O)C=C(C)C | [196216](https://pubchem.ncbi.nlm.nih.gov/compound/196216) |
| 62 | [Dibutyl phthalate](https://cb.imsc.res.in/imppat/phytochemical-detailedpage/IMPHY007903) | CCCCOC(=O)c1ccccc1C(=O)OCCCC | [3026](https://pubchem.ncbi.nlm.nih.gov/compound/3026) |
| 63 | [ar-Turmerone](https://cb.imsc.res.in/imppat/phytochemical-detailedpage/IMPHY012381) | CC(=CC(=O)C[C@@H](c1ccc(cc1)C)C)C | [160512](https://pubchem.ncbi.nlm.nih.gov/compound/160512) |
| 64 | [Melanin](https://cb.imsc.res.in/imppat/phytochemical-detailedpage/IMPHY012901) | Cc1c2[nH]cc3c2c(c(=O)c1=O)c1c[nH]c2c1c3c(=O)c(=O)c2C | [6325610](https://pubchem.ncbi.nlm.nih.gov/compound/6325610) |
| 65 | [Syringic acid](https://cb.imsc.res.in/imppat/phytochemical-detailedpage/IMPHY000687) | COc1cc(cc(c1O)OC)C(=O)O | [10742](https://pubchem.ncbi.nlm.nih.gov/compound/10742) |
| 66 | [Tetratriacontane](https://cb.imsc.res.in/imppat/phytochemical-detailedpage/IMPHY002954) | CCCCCCCCCCCCCCCCCCCCCCCCCCCCCCCCCC | [26519](https://pubchem.ncbi.nlm.nih.gov/compound/26519) |
| 67 | [Benzoic acid](https://cb.imsc.res.in/imppat/phytochemical-detailedpage/IMPHY002962) | OC(=O)c1ccccc1 | [243](https://pubchem.ncbi.nlm.nih.gov/compound/243) |
| 68 | [Calceolarioside A](https://cb.imsc.res.in/imppat/phytochemical-detailedpage/IMPHY004561) | OC[C@H]1O[C@@H](OCCc2ccc(c(c2)O)O)[C@@H]([C@H]([C@@H]1OC(=O)/C=C/c1ccc(c(c1)O)O)O)O | [5273566](https://pubchem.ncbi.nlm.nih.gov/compound/5273566) |
| 69 | [methyl (1S,4aS,5S,6R,7R,7aR)-6-benzoyloxy-5-hydroxy-7-methyl-1-[(2S,3R,4S,5S,6R)-3,4,5-trihydroxy-6-(hydroxymethyl)oxan-2-yl]oxy-1,4a,5,6,7,7a-hexahydrocyclopenta[c]pyran-4-carboxylate](https://cb.imsc.res.in/imppat/phytochemical-detailedpage/IMPHY007638) | OC[C@H]1O[C@@H](O[C@@H]2OC=C([C@@H]3[C@H]2[C@@H](C)[C@H]([C@H]3O)OC(=O)c2ccccc2)C(=O)OC)[C@@H]([C@H]([C@@H]1O)O)O | [15747848](https://pubchem.ncbi.nlm.nih.gov/compound/15747848) |
| 70 | [Arbortristoside D](https://cb.imsc.res.in/imppat/phytochemical-detailedpage/IMPHY007639) | OC[C@H]1O[C@@H](O[C@@H]2OC=C([C@@H]3[C@H]2[C@@H](COC(=O)/C=C/c2ccc(c(c2)O)O)[C@H]([C@H]3O)O)C(=O)OC)[C@@H]([C@H]([C@@H]1O)O)O | [14632886](https://pubchem.ncbi.nlm.nih.gov/compound/14632886) |
| 71 | [Arbortristoside E](https://cb.imsc.res.in/imppat/phytochemical-detailedpage/IMPHY007640) | COc1ccc(cc1)/C=C/C(=O)OC[C@H]1O[C@@H](O[C@@H]2OC=C([C@@H]3[C@H]2[C@@H](C)[C@H]([C@H]3O)O)C(=O)OC)[C@@H]([C@H]([C@@H]1O)O)O | [14632884](https://pubchem.ncbi.nlm.nih.gov/compound/14632884) |
| 72 | [Arborside A](https://cb.imsc.res.in/imppat/phytochemical-detailedpage/IMPHY008916) | OC[C@H]1O[C@@H](O[C@@H]2OC=C([C@@H]3[C@H]2[C@@H](C)[C@H]([C@H]3OC(=O)c2ccccc2)OC(=O)c2ccccc2)C(=O)OC)[C@@H]([C@H]([C@@H]1O)O)O | [182902](https://pubchem.ncbi.nlm.nih.gov/compound/182902) |
| 73 | [Arborside B](https://cb.imsc.res.in/imppat/phytochemical-detailedpage/IMPHY008917) | OC[C@H]1O[C@@H](O[C@@H]2OC=C([C@@H]3[C@H]2[C@@H](C)[C@H](C3)OC(=O)c2ccccc2)C(=O)OC)[C@@H]([C@H]([C@@H]1O)O)O | [182903](https://pubchem.ncbi.nlm.nih.gov/compound/182903) |
| 74 | [Nyctanthic acid](https://cb.imsc.res.in/imppat/phytochemical-detailedpage/IMPHY009338) | OC(=O)CC[C@@]1(C)[C@@H](CC[C@@]2([C@@H]1CC=C1[C@@]2(C)CC[C@@]2([C@H]1CC(C)(C)CC2)C)C)C(=C)C | [12313631](https://pubchem.ncbi.nlm.nih.gov/compound/12313631) |
| 75 | [Nonacosane](https://cb.imsc.res.in/imppat/phytochemical-detailedpage/IMPHY009482) | CCCCCCCCCCCCCCCCCCCCCCCCCCCCC | [12409](https://pubchem.ncbi.nlm.nih.gov/compound/12409) |
| 76 | [Tritriacontane](https://cb.imsc.res.in/imppat/phytochemical-detailedpage/IMPHY009483) | CCCCCCCCCCCCCCCCCCCCCCCCCCCCCCCCC | [12411](https://pubchem.ncbi.nlm.nih.gov/compound/12411) |
| 77 | [6beta-Hydroxyloganin](https://cb.imsc.res.in/imppat/phytochemical-detailedpage/IMPHY009922) | OC[C@H]1O[C@@H](O[C@@H]2OC=C([C@@H]3[C@H]2[C@@H](C)[C@H]([C@H]3O)O)C(=O)OC)[C@@H]([C@H]([C@@H]1O)O)O | [158641](https://pubchem.ncbi.nlm.nih.gov/compound/158641) |
| 78 | [Butyric acid](https://cb.imsc.res.in/imppat/phytochemical-detailedpage/IMPHY010081) | CCCC(=O)O | [264](https://pubchem.ncbi.nlm.nih.gov/compound/264) |
| 79 | [Arbortristoside A](https://cb.imsc.res.in/imppat/phytochemical-detailedpage/IMPHY010708) | OC[C@H]1O[C@@H](O[C@@H]2OC=C([C@@H]3[C@H]2[C@@H](C)[C@H]([C@H]3O)OC(=O)/C=C/c2ccc(cc2)OC)C(=O)OC)[C@@H]([C@H]([C@@H]1O)O)O | no id |
| 80 | [Friedelin](https://cb.imsc.res.in/imppat/phytochemical-detailedpage/IMPHY011688) | O=C1CC[C@@H]2[C@]([C@H]1C)(C)CC[C@H]1[C@@]2(C)CC[C@@]2([C@]1(C)CC[C@@]1([C@H]2CC(C)(C)CC1)C)C | [91472](https://pubchem.ncbi.nlm.nih.gov/compound/91472) |
| 81 | [Oleanolic acid](https://cb.imsc.res.in/imppat/phytochemical-detailedpage/IMPHY011826) | O[C@H]1CC[C@]2([C@H](C1(C)C)CC[C@@]1([C@@H]2CC=C2[C@@]1(C)CC[C@@]1([C@H]2CC(C)(C)CC1)C(=O)O)C)C | [10494](https://pubchem.ncbi.nlm.nih.gov/compound/10494) |
| 82 | [Ursolic acid](https://cb.imsc.res.in/imppat/phytochemical-detailedpage/IMPHY011880) | C[C@@H]1CC[C@]2([C@@H]([C@H]1C)C1=CC[C@H]3[C@@]([C@@]1(CC2)C)(C)CC[C@@H]1[C@]3(C)CC[C@@H](C1(C)C)O)C(=O)O | [64945](https://pubchem.ncbi.nlm.nih.gov/compound/64945) |
| 83 | [3,4-Dihydroxybenzoic acid](https://cb.imsc.res.in/imppat/phytochemical-detailedpage/IMPHY011883) | OC(=O)c1ccc(c(c1)O)O | [72](https://pubchem.ncbi.nlm.nih.gov/compound/72) |
| 84 | [Gallic acid](https://cb.imsc.res.in/imppat/phytochemical-detailedpage/IMPHY012021) | OC(=O)c1cc(O)c(c(c1)O)O | [370](https://pubchem.ncbi.nlm.nih.gov/compound/370) |
| 85 | [Loganin pentaacetate](https://cb.imsc.res.in/imppat/phytochemical-detailedpage/IMPHY012564) | COC(=O)C1=CO[C@H]([C@H]2[C@@H]1C[C@@H]([C@@H]2C)OC(=O)C)O[C@@H]1O[C@H](COC(=O)C)[C@H]([C@@H]([C@H]1OC(=O)C)OC(=O)C)OC(=O)C | [443344](https://pubchem.ncbi.nlm.nih.gov/compound/443344) |
| 86 | [Arborside D](https://cb.imsc.res.in/imppat/phytochemical-detailedpage/IMPHY014596) | OC[C@H]1O[C@@H](O[C@@H]2OC=C([C@@H]3[C@H]2[C@@H](COC(=O)c2ccccc2)[C@H]([C@H]3O)O)C(=O)OC)[C@@H]([C@H]([C@@H]1O)O)O | [101685135](https://pubchem.ncbi.nlm.nih.gov/compound/101685135) |
| 87 | [beta-Sitosterol](https://cb.imsc.res.in/imppat/phytochemical-detailedpage/IMPHY014836) | CC[C@@H](C(C)C)CC[C@H]([C@H]1CC[C@@H]2[C@]1(C)CC[C@H]1[C@H]2CC=C2[C@]1(C)CC[C@@H](C2)O)C | [222284](https://pubchem.ncbi.nlm.nih.gov/compound/222284) |
| 88 | [Daucosterol](https://cb.imsc.res.in/imppat/phytochemical-detailedpage/IMPHY014838) | CC[C@@H](C(C)C)CC[C@H]([C@H]1CC[C@@H]2[C@]1(C)CC[C@H]1[C@H]2CC=C2[C@]1(C)CC[C@@H](C2)O[C@@H]1O[C@H](CO)[C@H]([C@@H]([C@H]1O)O)O)C | [5742590](https://pubchem.ncbi.nlm.nih.gov/compound/5742590) |
| 89 | [Glyceryl 1,3-distearate](https://cb.imsc.res.in/imppat/phytochemical-detailedpage/IMPHY000442) | CCCCCCCCCCCCCCCCCC(=O)OCC(COC(=O)CCCCCCCCCCCCCCCCC)O | [101269](https://pubchem.ncbi.nlm.nih.gov/compound/101269) |
| 90 | [Stearic acid](https://cb.imsc.res.in/imppat/phytochemical-detailedpage/IMPHY004631) | CCCCCCCCCCCCCCCCCC(=O)O | [5281](https://pubchem.ncbi.nlm.nih.gov/compound/5281) |
| 91 | [Docosanoic acid](https://cb.imsc.res.in/imppat/phytochemical-detailedpage/IMPHY007212) | CCCCCCCCCCCCCCCCCCCCCC(=O)O | [8215](https://pubchem.ncbi.nlm.nih.gov/compound/8215) |
| 92 | [Arachidic acid](https://cb.imsc.res.in/imppat/phytochemical-detailedpage/IMPHY011394) | CCCCCCCCCCCCCCCCCCCC(=O)O | [10467](https://pubchem.ncbi.nlm.nih.gov/compound/10467) |
| 93 | [4-O-beta-D-Mannopyranosyl-D-mannopyranose](https://cb.imsc.res.in/imppat/phytochemical-detailedpage/IMPHY011419) | OC[C@H]1OC(O)[C@H]([C@H]([C@@H]1O[C@@H]1O[C@H](CO)[C@H]([C@@H]([C@@H]1O)O)O)O)O | [152109](https://pubchem.ncbi.nlm.nih.gov/compound/152109) |
| 94 | [2,3,6-Tri-o-methyl-d-glucose](https://cb.imsc.res.in/imppat/phytochemical-detailedpage/IMPHY013328) | COC[C@H]([C@H]([C@@H]([C@H](C=O)OC)OC)O)O | [22212995](https://pubchem.ncbi.nlm.nih.gov/compound/22212995) |
| 95 | [2,3-di-O-methyl-d-mannose](https://cb.imsc.res.in/imppat/phytochemical-detailedpage/IMPHY014641) | [2H]CO[C@@H]([C@@H]([C@@H](CO)O)O)[C@H](OC[2H])C=O | [129698394](https://pubchem.ncbi.nlm.nih.gov/compound/129698394) |
| 96 | [2,3,6-Tri-O-methyl-D-mannose](https://cb.imsc.res.in/imppat/phytochemical-detailedpage/IMPHY014747) | COC[C@H]([C@H]([C@@H]([C@@H](C=O)OC)OC)O)O | [54211230](https://pubchem.ncbi.nlm.nih.gov/compound/54211230) |
| 97 | [cis-11-Eicosenoic acid](https://cb.imsc.res.in/imppat/phytochemical-detailedpage/IMPHY014861) | CCCCCCCC/C=CCCCCCCCCCC(=O)O | [5282768](https://pubchem.ncbi.nlm.nih.gov/compound/5282768) |
| 98 | [D-Glucose](https://cb.imsc.res.in/imppat/phytochemical-detailedpage/IMPHY014893) | OC[C@H]1OC(O)[C@@H]([C@H]([C@@H]1O)O)O | [5793](https://pubchem.ncbi.nlm.nih.gov/compound/5793) |
| 99 | [beta-D-Glucose](https://cb.imsc.res.in/imppat/phytochemical-detailedpage/IMPHY014925) | OC[C@H]1O[C@@H](O)[C@@H]([C@H]([C@@H]1O)O)O | [64689](https://pubchem.ncbi.nlm.nih.gov/compound/64689) |
| 100 | [D-Mannose](https://cb.imsc.res.in/imppat/phytochemical-detailedpage/IMPHY015000) | OC[C@H]1OC(O)[C@H]([C@H]([C@@H]1O)O)O | [18950](https://pubchem.ncbi.nlm.nih.gov/compound/18950) |
| 101 | [Naringetol](https://cb.imsc.res.in/imppat/phytochemical-detailedpage/IMPHY010550) | Oc1ccc(cc1)[C@@H]1CC(=O)c2c(O1)cc(cc2O)O | [439246](https://pubchem.ncbi.nlm.nih.gov/compound/439246) |
| 102 | [Myristic acid](https://cb.imsc.res.in/imppat/phytochemical-detailedpage/IMPHY000060) | CCCCCCCCCCCCCC(=O)O | [11005](https://pubchem.ncbi.nlm.nih.gov/compound/11005) |
| 103 | [Tetracosanoic acid](https://cb.imsc.res.in/imppat/phytochemical-detailedpage/IMPHY000165) | CCCCCCCCCCCCCCCCCCCCCCCC(=O)O | [11197](https://pubchem.ncbi.nlm.nih.gov/compound/11197) |
| 104 | [Octacosane](https://cb.imsc.res.in/imppat/phytochemical-detailedpage/IMPHY009481) | CCCCCCCCCCCCCCCCCCCCCCCCCCCC | [12408](https://pubchem.ncbi.nlm.nih.gov/compound/12408) |
| 105 | [2-Methyloctadecane](https://cb.imsc.res.in/imppat/phytochemical-detailedpage/IMPHY012150) | CCCCCCCCCCCCCCCCC(C)C | [15264](https://pubchem.ncbi.nlm.nih.gov/compound/15264) |
| 106 | [Phytol](https://cb.imsc.res.in/imppat/phytochemical-detailedpage/IMPHY012712) | OC/C=C(/CCC[C@@H](CCC[C@@H](CCCC(C)C)C)C)C | [5280435](https://pubchem.ncbi.nlm.nih.gov/compound/5280435) |
| 107 | [D-Fructose](https://cb.imsc.res.in/imppat/phytochemical-detailedpage/IMPHY014916) | OCC1(O)OC[C@H]([C@H]([C@@H]1O)O)O | [2723872](https://pubchem.ncbi.nlm.nih.gov/compound/2723872) |
| 108 | [2,6-Bis(tert-butyl)-4-(1-methyl-1-phenylethyl)phenol](https://cb.imsc.res.in/imppat/phytochemical-detailedpage/IMPHY017146) | CC(c1ccccc1)(c1cc(c(c(c1)C(C)(C)C)O)C(C)(C)C)C | [161829](https://pubchem.ncbi.nlm.nih.gov/compound/161829) |
| 109 | [1,6-Diphenyl-1,5-hexadiene](https://cb.imsc.res.in/imppat/phytochemical-detailedpage/IMPHY017427) | C(/C=C/c1ccccc1)C/C=C/c1ccccc1 | [5370615](https://pubchem.ncbi.nlm.nih.gov/compound/5370615) |
| 110 | [3-(Hydroxy-phenyl-methyl)-2,3-dimethyl-octan-4-one](https://cb.imsc.res.in/imppat/phytochemical-detailedpage/IMPHY017483) | CCCCC(=O)C(C(c1ccccc1)O)(C(C)C)C | [559104](https://pubchem.ncbi.nlm.nih.gov/compound/559104) |
| 111 | [Diethylene glycol dibenzoate](https://cb.imsc.res.in/imppat/phytochemical-detailedpage/IMPHY017776) | O=C(c1ccccc1)OCCOCCOC(=O)c1ccccc1 | [8437](https://pubchem.ncbi.nlm.nih.gov/compound/8437) |
| 112 | [Liriodenine](https://cb.imsc.res.in/imppat/phytochemical-detailedpage/IMPHY002753) | O=C1c2ccccc2-c2c3c1nccc3cc1c2OCO1 | [10144](https://pubchem.ncbi.nlm.nih.gov/compound/10144) |
| 113 | [Kaurenoic acid](https://cb.imsc.res.in/imppat/phytochemical-detailedpage/IMPHY011780) | C=C1C[C@@]23C[C@H]1CC[C@H]3[C@]1([C@H](CC2)[C@@](C)(CCC1)C(=O)O)C | [73062](https://pubchem.ncbi.nlm.nih.gov/compound/73062) |
| 114 | [Perillyl alcohol](https://cb.imsc.res.in/imppat/phytochemical-detailedpage/IMPHY000619) | OCC1=CCC(CC1)C(=C)C | [10819](https://pubchem.ncbi.nlm.nih.gov/compound/10819) |
| 115 | [Isovaleric acid](https://cb.imsc.res.in/imppat/phytochemical-detailedpage/IMPHY001109) | CC(CC(=O)O)C | [10430](https://pubchem.ncbi.nlm.nih.gov/compound/10430) |
| 116 | [Carvacrol](https://cb.imsc.res.in/imppat/phytochemical-detailedpage/IMPHY001246) | CC(c1ccc(c(c1)O)C)C | [10364](https://pubchem.ncbi.nlm.nih.gov/compound/10364) |
| 117 | [Elemicin](https://cb.imsc.res.in/imppat/phytochemical-detailedpage/IMPHY001351) | C=CCc1cc(OC)c(c(c1)OC)OC | [10248](https://pubchem.ncbi.nlm.nih.gov/compound/10248) |
| 118 | [Pentadecanoic acid](https://cb.imsc.res.in/imppat/phytochemical-detailedpage/IMPHY002667) | CCCCCCCCCCCCCCC(=O)O | [13849](https://pubchem.ncbi.nlm.nih.gov/compound/13849) |
| 119 | [Lauric acid](https://cb.imsc.res.in/imppat/phytochemical-detailedpage/IMPHY003016) | CCCCCCCCCCCC(=O)O | [3893](https://pubchem.ncbi.nlm.nih.gov/compound/3893) |
| 120 | [Myrcene](https://cb.imsc.res.in/imppat/phytochemical-detailedpage/IMPHY003485) | C=CC(=C)CCC=C(C)C | [31253](https://pubchem.ncbi.nlm.nih.gov/compound/31253) |
| 121 | [Ethyl hexanoate](https://cb.imsc.res.in/imppat/phytochemical-detailedpage/IMPHY003512) | CCCCCC(=O)OCC | [31265](https://pubchem.ncbi.nlm.nih.gov/compound/31265) |
| 122 | [Bicyclogermacrene](https://cb.imsc.res.in/imppat/phytochemical-detailedpage/IMPHY003616) | C/C/1=CCC/C(=C/[C@H]2[C@@H](CC1)C2(C)C)/C | [13894537](https://pubchem.ncbi.nlm.nih.gov/compound/13894537) |
| 123 | [Edulan I](https://cb.imsc.res.in/imppat/phytochemical-detailedpage/IMPHY003765) | C[C@H]1CC=C2[C@@](O1)(C)C=CCC2(C)C | no id |
| 124 | [Labd-14-ene, 8,13-epoxy-, (13S)-](https://cb.imsc.res.in/imppat/phytochemical-detailedpage/IMPHY003883) | C=C[C@@]1(C)CC[C@H]2[C@@](O1)(C)CC[C@@H]1[C@]2(C)CCCC1(C)C | [6432025](https://pubchem.ncbi.nlm.nih.gov/compound/6432025) |
| 125 | [gamma-Terpinene](https://cb.imsc.res.in/imppat/phytochemical-detailedpage/IMPHY003982) | CC1=CCC(=CC1)C(C)C | [7461](https://pubchem.ncbi.nlm.nih.gov/compound/7461) |
| 126 | [Verbenone](https://cb.imsc.res.in/imppat/phytochemical-detailedpage/IMPHY004077) | CC1=CC(=O)C2CC1C2(C)C | [29025](https://pubchem.ncbi.nlm.nih.gov/compound/29025) |
| 127 | [2-Heptanone](https://cb.imsc.res.in/imppat/phytochemical-detailedpage/IMPHY004121) | CCCCCC(=O)C | [8051](https://pubchem.ncbi.nlm.nih.gov/compound/8051) |
| 128 | [Ethyl butyrate](https://cb.imsc.res.in/imppat/phytochemical-detailedpage/IMPHY006033) | CCCC(=O)OCC | [7762](https://pubchem.ncbi.nlm.nih.gov/compound/7762) |
| 129 | [Ethyl 3-hydroxyhexanoate](https://cb.imsc.res.in/imppat/phytochemical-detailedpage/IMPHY006314) | CCCC(CC(=O)OCC)O | [61293](https://pubchem.ncbi.nlm.nih.gov/compound/61293) |
| 130 | [Methyleugenol](https://cb.imsc.res.in/imppat/phytochemical-detailedpage/IMPHY006696) | C=CCc1ccc(c(c1)OC)OC | [7127](https://pubchem.ncbi.nlm.nih.gov/compound/7127) |
| 131 | [2-Pentanone](https://cb.imsc.res.in/imppat/phytochemical-detailedpage/IMPHY006989) | CCCC(=O)C | [7895](https://pubchem.ncbi.nlm.nih.gov/compound/7895) |
| 132 | [Furfural](https://cb.imsc.res.in/imppat/phytochemical-detailedpage/IMPHY007041) | O=Cc1ccco1 | [7362](https://pubchem.ncbi.nlm.nih.gov/compound/7362) |
| 133 | [Methyl acetate](https://cb.imsc.res.in/imppat/phytochemical-detailedpage/IMPHY007146) | COC(=O)C | [6584](https://pubchem.ncbi.nlm.nih.gov/compound/6584) |
| 134 | [Hexanoic acid](https://cb.imsc.res.in/imppat/phytochemical-detailedpage/IMPHY007354) | CCCCCC(=O)O | [8892](https://pubchem.ncbi.nlm.nih.gov/compound/8892) |
| 135 | [beta-Cubebene](https://cb.imsc.res.in/imppat/phytochemical-detailedpage/IMPHY007376) | CC([C@@H]1CC[C@H]([C@]23[C@H]1[C@H]2C(=C)CC3)C)C | [93081](https://pubchem.ncbi.nlm.nih.gov/compound/93081) |
| 136 | [Ethyl acetate](https://cb.imsc.res.in/imppat/phytochemical-detailedpage/IMPHY007417) | CCOC(=O)C | [8857](https://pubchem.ncbi.nlm.nih.gov/compound/8857) |
| 137 | [Spathulenol](https://cb.imsc.res.in/imppat/phytochemical-detailedpage/IMPHY007840) | C=C1CC[C@@H]2[C@H]([C@H]3[C@H]1CC[C@]3(C)O)C2(C)C | [92231](https://pubchem.ncbi.nlm.nih.gov/compound/92231) |
| 138 | [Ethyl octanoate](https://cb.imsc.res.in/imppat/phytochemical-detailedpage/IMPHY008066) | CCCCCCCC(=O)OCC | [7799](https://pubchem.ncbi.nlm.nih.gov/compound/7799) |
| 139 | [delta-Guaiene](https://cb.imsc.res.in/imppat/phytochemical-detailedpage/IMPHY008946) | CC(=C)[C@@H]1CCC(=C2[C@@H](C1)[C@@H](C)CC2)C | [94275](https://pubchem.ncbi.nlm.nih.gov/compound/94275) |
| 140 | [2-Nonanol](https://cb.imsc.res.in/imppat/phytochemical-detailedpage/IMPHY009626) | CCCCCCCC(O)C | [12367](https://pubchem.ncbi.nlm.nih.gov/compound/12367) |
| 141 | [2-Nonanone](https://cb.imsc.res.in/imppat/phytochemical-detailedpage/IMPHY009642) | CCCCCCCC(=O)C | [13187](https://pubchem.ncbi.nlm.nih.gov/compound/13187) |
| 142 | [Ethyl isovalerate](https://cb.imsc.res.in/imppat/phytochemical-detailedpage/IMPHY009961) | CC/C=CC[C@H]1C(=O)CC[C@@H]1C(=O)OC | [6430765](https://pubchem.ncbi.nlm.nih.gov/compound/6430765) |
| 143 | [Isoelemicin](https://cb.imsc.res.in/imppat/phytochemical-detailedpage/IMPHY010002) | C/C=C/c1cc(OC)c(c(c1)OC)OC | [5318557](https://pubchem.ncbi.nlm.nih.gov/compound/5318557) |
| 144 | [beta-Elemene](https://cb.imsc.res.in/imppat/phytochemical-detailedpage/IMPHY010080) | C=C[C@]1(C)CC[C@H](C[C@H]1C(=C)C)C(=C)C | [6918391](https://pubchem.ncbi.nlm.nih.gov/compound/6918391) |
| 145 | [alpha-Bergamotenol](https://cb.imsc.res.in/imppat/phytochemical-detailedpage/IMPHY011356) | OC/C(=CCCC1(C)C2CC=C(C1C2)C)/C | [5368743](https://pubchem.ncbi.nlm.nih.gov/compound/5368743) |
| 146 | [4-Carvomenthenol](https://cb.imsc.res.in/imppat/phytochemical-detailedpage/IMPHY011396) | CC1=CCC(CC1)(O)C(C)C | [11230](https://pubchem.ncbi.nlm.nih.gov/compound/11230) |
| 147 | [(S,1Z,6Z)-8-Isopropyl-1-methyl-5-methylenecyclodeca-1,6-diene](https://cb.imsc.res.in/imppat/phytochemical-detailedpage/IMPHY011586) | C/C/1=C/CCC(=C)/C=C[C@@H](CC1)C(C)C | [91723653](https://pubchem.ncbi.nlm.nih.gov/compound/91723653) |
| 148 | [d-Borneol](https://cb.imsc.res.in/imppat/phytochemical-detailedpage/IMPHY011590) | O[C@@H]1C[C@H]2C([C@@]1(C)CC2)(C)C | [61060](https://pubchem.ncbi.nlm.nih.gov/compound/61060) |
| 149 | [Terpinolene](https://cb.imsc.res.in/imppat/phytochemical-detailedpage/IMPHY011599) | CC1=CCC(=C(C)C)CC1 | [11463](https://pubchem.ncbi.nlm.nih.gov/compound/11463) |
| 150 | [Farnesol](https://cb.imsc.res.in/imppat/phytochemical-detailedpage/IMPHY011632) | OC/C=C(/CC/C=C(/CCC=C(C)C)C)C | [445070](https://pubchem.ncbi.nlm.nih.gov/compound/445070) |
| 151 | [(2Z,6E)-Farnesol](https://cb.imsc.res.in/imppat/phytochemical-detailedpage/IMPHY011633) | OC/C=C(CC/C=C(/CCC=C(C)C)C)/C | [1549108](https://pubchem.ncbi.nlm.nih.gov/compound/1549108) |
| 152 | [alpha-Terpinene](https://cb.imsc.res.in/imppat/phytochemical-detailedpage/IMPHY011643) | CC1=CC=C(CC1)C(C)C | [7462](https://pubchem.ncbi.nlm.nih.gov/compound/7462) |
| 153 | [alpha-Eudesmol](https://cb.imsc.res.in/imppat/phytochemical-detailedpage/IMPHY011709) | CC1=CCC[C@]2([C@H]1C[C@@H](CC2)C(O)(C)C)C | [92762](https://pubchem.ncbi.nlm.nih.gov/compound/92762) |
| 154 | [Humulene](https://cb.imsc.res.in/imppat/phytochemical-detailedpage/IMPHY011761) | C/C/1=CCC(C)(C)/C=C/C/C(=C/CC1)/C | [5281520](https://pubchem.ncbi.nlm.nih.gov/compound/5281520) |
| 155 | [alpha-Farnesene](https://cb.imsc.res.in/imppat/phytochemical-detailedpage/IMPHY011817) | C=C/C(=C/C/C=C(/CCC=C(C)C)C)/C | [5281516](https://pubchem.ncbi.nlm.nih.gov/compound/5281516) |
| 156 | [(+)-delta-Cadinene](https://cb.imsc.res.in/imppat/phytochemical-detailedpage/IMPHY011957) | CC1=C[C@@H]2C(=C(C)CC[C@H]2C(C)C)CC1 | [441005](https://pubchem.ncbi.nlm.nih.gov/compound/441005) |
| 157 | [(+)-beta-Phellandrene](https://cb.imsc.res.in/imppat/phytochemical-detailedpage/IMPHY011965) | CC([C@@H]1CCC(=C)C=C1)C | [442484](https://pubchem.ncbi.nlm.nih.gov/compound/442484) |
| 158 | [(-)-trans-Carveol](https://cb.imsc.res.in/imppat/phytochemical-detailedpage/IMPHY011988) | CC(=C)[C@@H]1CC=C([C@H](C1)O)C | [94221](https://pubchem.ncbi.nlm.nih.gov/compound/94221) |
| 159 | [Linalool](https://cb.imsc.res.in/imppat/phytochemical-detailedpage/IMPHY012058) | C=CC(CCC=C(C)C)(O)C | [6549](https://pubchem.ncbi.nlm.nih.gov/compound/6549) |
| 160 | [Carvone](https://cb.imsc.res.in/imppat/phytochemical-detailedpage/IMPHY012075) | CC(=C)C1CC=C(C(=O)C1)C | [7439](https://pubchem.ncbi.nlm.nih.gov/compound/7439) |
| 161 | [Methyl octanoate](https://cb.imsc.res.in/imppat/phytochemical-detailedpage/IMPHY012097) | CCCCCCCC(=O)OC | [8091](https://pubchem.ncbi.nlm.nih.gov/compound/8091) |
| 162 | [Nonanoic acid](https://cb.imsc.res.in/imppat/phytochemical-detailedpage/IMPHY012100) | CCCCCCCCC(=O)O | [8158](https://pubchem.ncbi.nlm.nih.gov/compound/8158) |
| 163 | [beta-Pinene](https://cb.imsc.res.in/imppat/phytochemical-detailedpage/IMPHY012147) | C=C1CCC2CC1C2(C)C | [14896](https://pubchem.ncbi.nlm.nih.gov/compound/14896) |
| 164 | [alpha-Terpineol](https://cb.imsc.res.in/imppat/phytochemical-detailedpage/IMPHY012160) | CC1=CCC(CC1)C(O)(C)C | [17100](https://pubchem.ncbi.nlm.nih.gov/compound/17100) |
| 165 | [Sabinene](https://cb.imsc.res.in/imppat/phytochemical-detailedpage/IMPHY012165) | C=C1CCC2(C1C2)C(C)C | [18818](https://pubchem.ncbi.nlm.nih.gov/compound/18818) |
| 166 | [(+)-trans-Piperitenol](https://cb.imsc.res.in/imppat/phytochemical-detailedpage/IMPHY012255) | CC1=C[C@@H]([C@H](CC1)C(C)C)O | [85568](https://pubchem.ncbi.nlm.nih.gov/compound/85568) |
| 167 | [(-)-alpha-Cadinol](https://cb.imsc.res.in/imppat/phytochemical-detailedpage/IMPHY012586) | CC1=CC2C(CC1)[C@@](C)(O)CC[C@@H]2C(C)C | [6431302](https://pubchem.ncbi.nlm.nih.gov/compound/6431302) |
| 168 | [Selin-11-en-4alpha-ol](https://cb.imsc.res.in/imppat/phytochemical-detailedpage/IMPHY012596) | CC(=C)[C@@H]1CC[C@@]2([C@@H](C1)[C@](C)(O)CCC2)C | [15560330](https://pubchem.ncbi.nlm.nih.gov/compound/15560330) |
| 169 | [Levomenol](https://cb.imsc.res.in/imppat/phytochemical-detailedpage/IMPHY012665) | CC(=CCC[C@@]([C@H]1CCC(=CC1)C)(O)C)C | [442343](https://pubchem.ncbi.nlm.nih.gov/compound/442343) |
| 170 | [Caryophyllene oxide](https://cb.imsc.res.in/imppat/phytochemical-detailedpage/IMPHY012667) | C=C1CC[C@H]2O[C@@]2(CC[C@@H]2[C@@H]1CC2(C)C)C | [1742210](https://pubchem.ncbi.nlm.nih.gov/compound/1742210) |
| 171 | [(Z)-beta-Ocimene](https://cb.imsc.res.in/imppat/phytochemical-detailedpage/IMPHY012739) | C=C/C(=CCC=C(C)C)/C | [5320250](https://pubchem.ncbi.nlm.nih.gov/compound/5320250) |
| 172 | [Methyl (E)-2-octenoate](https://cb.imsc.res.in/imppat/phytochemical-detailedpage/IMPHY013035) | CCCCC/C=CC(=O)OC | [11040937](https://pubchem.ncbi.nlm.nih.gov/compound/11040937) |
| 173 | [Caswell No. 264AB](https://cb.imsc.res.in/imppat/phytochemical-detailedpage/IMPHY014806) | CC([C@@H]1CC[C@H]([C@]23[C@H]1[C@H]2C(=CC3)C)C)C | [442359](https://pubchem.ncbi.nlm.nih.gov/compound/442359) |
| 174 | [alpha-Phellandrene](https://cb.imsc.res.in/imppat/phytochemical-detailedpage/IMPHY014811) | CC1=CCC(C=C1)C(C)C | [7460](https://pubchem.ncbi.nlm.nih.gov/compound/7460) |
| 175 | [Aromadendrene](https://cb.imsc.res.in/imppat/phytochemical-detailedpage/IMPHY014817) | CC1CCC2C1C1C(C1(C)C)CCC2=C | [91354](https://pubchem.ncbi.nlm.nih.gov/compound/91354) |
| 176 | [beta-Caryophyllene](https://cb.imsc.res.in/imppat/phytochemical-detailedpage/IMPHY014831) | C/C/1=CCCC(=C)[C@@H]2[C@@H](CC1)C(C2)(C)C | [5281515](https://pubchem.ncbi.nlm.nih.gov/compound/5281515) |
| 177 | [(E)-beta-ocimene](https://cb.imsc.res.in/imppat/phytochemical-detailedpage/IMPHY014835) | C=C/C(=C/CC=C(C)C)/C | [5281553](https://pubchem.ncbi.nlm.nih.gov/compound/5281553) |
| 178 | [Bornyl acetate](https://cb.imsc.res.in/imppat/phytochemical-detailedpage/IMPHY014847) | CC(=O)OC1CC2C(C1(C)CC2)(C)C | [6448](https://pubchem.ncbi.nlm.nih.gov/compound/6448) |
| 179 | [Camphene](https://cb.imsc.res.in/imppat/phytochemical-detailedpage/IMPHY014852) | C=C1C2CCC(C1(C)C)C2 | [6616](https://pubchem.ncbi.nlm.nih.gov/compound/6616) |
| 180 | [2-Cyclohexen-1-ol, 3-methyl-6-(1-methylethyl)-, (1R,6S)-rel-](https://cb.imsc.res.in/imppat/phytochemical-detailedpage/IMPHY014873) | CC1=C[C@@H]([C@@H](CC1)C(C)C)O | [85567](https://pubchem.ncbi.nlm.nih.gov/compound/85567) |
| 181 | [cis-Sabinene hydrate](https://cb.imsc.res.in/imppat/phytochemical-detailedpage/IMPHY014874) | C[C@@H]1CC[C@@]2(C1C2)C(C)C | [101629835](https://pubchem.ncbi.nlm.nih.gov/compound/101629835) |
| 182 | [Geraniol](https://cb.imsc.res.in/imppat/phytochemical-detailedpage/IMPHY014923) | OC/C=C(/CCC=C(C)C)C | [637566](https://pubchem.ncbi.nlm.nih.gov/compound/637566) |
| 183 | [Ledol](https://cb.imsc.res.in/imppat/phytochemical-detailedpage/IMPHY014986) | C[C@@H]1CC[C@H]2[C@@H]1[C@H]1[C@H](C1(C)C)CC[C@@]2(C)O | [92812](https://pubchem.ncbi.nlm.nih.gov/compound/92812) |
| 184 | [Limonene](https://cb.imsc.res.in/imppat/phytochemical-detailedpage/IMPHY014988) | CC1=CCC(CC1)C(=C)C | [22311](https://pubchem.ncbi.nlm.nih.gov/compound/22311) |
| 185 | [Nerolidol](https://cb.imsc.res.in/imppat/phytochemical-detailedpage/IMPHY015022) | C=CC(CC/C=C(/CCC=C(C)C)C)(O)C | [5284507](https://pubchem.ncbi.nlm.nih.gov/compound/5284507) |
| 186 | [Piperitone](https://cb.imsc.res.in/imppat/phytochemical-detailedpage/IMPHY015042) | CC1=CC(=O)C(CC1)C(C)C | [6987](https://pubchem.ncbi.nlm.nih.gov/compound/6987) |
| 187 | [2-Cyclohexen-1-ol, 1-methyl-4-(1-methylethyl)-, trans-](https://cb.imsc.res.in/imppat/phytochemical-detailedpage/IMPHY015095) | CC([C@@H]1CC[C@@](C=C1)(C)O)C | [122484](https://pubchem.ncbi.nlm.nih.gov/compound/122484) |
| 188 | [alpha-Copaene](https://cb.imsc.res.in/imppat/phytochemical-detailedpage/IMPHY015123) | CC([C@@H]1CC[C@]2([C@@H]3[C@H]1C2C(=CC3)C)C)C | [70678558](https://pubchem.ncbi.nlm.nih.gov/compound/70678558) |
| 189 | [T-Muurolol](https://cb.imsc.res.in/imppat/phytochemical-detailedpage/IMPHY015128) | CC1=C[C@@H]2[C@H](CC1)[C@@](C)(O)CC[C@H]2C(C)C | [3084331](https://pubchem.ncbi.nlm.nih.gov/compound/3084331) |
| 190 | [3-Methyl-2-butanol](https://cb.imsc.res.in/imppat/phytochemical-detailedpage/IMPHY015348) | CC(C(C)C)O | [11732](https://pubchem.ncbi.nlm.nih.gov/compound/11732) |
| 191 | [Ethyl decanoate](https://cb.imsc.res.in/imppat/phytochemical-detailedpage/IMPHY015660) | CCCCCCCCCC(=O)OCC | [8048](https://pubchem.ncbi.nlm.nih.gov/compound/8048) |
| 192 | [Heptyl hexanoate](https://cb.imsc.res.in/imppat/phytochemical-detailedpage/IMPHY015720) | CCCCCCCOC(=O)CCCCC | [81464](https://pubchem.ncbi.nlm.nih.gov/compound/81464) |
| 193 | [Methyl hexanoate](https://cb.imsc.res.in/imppat/phytochemical-detailedpage/IMPHY015835) | CCCCCC(=O)OC | [7824](https://pubchem.ncbi.nlm.nih.gov/compound/7824) |
| 194 | [Allo-Aromadendrene](https://cb.imsc.res.in/imppat/phytochemical-detailedpage/IMPHY016012) | C[C@@H]1CC[C@H]2[C@@H]1C1C(C1(C)C)CCC2=C | [42608158](https://pubchem.ncbi.nlm.nih.gov/compound/42608158) |
| 195 | [trans-alpha-Bergamotene](https://cb.imsc.res.in/imppat/phytochemical-detailedpage/IMPHY016054) | CC(=CCC[C@]1(C)[C@H]2CC=C([C@@H]1C2)C)C | [6429302](https://pubchem.ncbi.nlm.nih.gov/compound/6429302) |
| 196 | [Abietal](https://cb.imsc.res.in/imppat/phytochemical-detailedpage/IMPHY016498) | O=C[C@]1(C)CCC[C@]2([C@H]1CC=C1[C@@H]2CCC(=C1)C(C)C)C | [443479](https://pubchem.ncbi.nlm.nih.gov/compound/443479) |
| 197 | [3-Butyl-5-methyldihydro-2(3H)-furanone](https://cb.imsc.res.in/imppat/phytochemical-detailedpage/IMPHY016617) | CCCCC1CC(OC1=O)C | [29718](https://pubchem.ncbi.nlm.nih.gov/compound/29718) |
| 198 | [Methyl 3-hydroxydecanoate](https://cb.imsc.res.in/imppat/phytochemical-detailedpage/IMPHY016739) | CCCCCCCCCCCCCC(CC(=O)OC)O | [103553](https://pubchem.ncbi.nlm.nih.gov/compound/103553) |
| 199 | [Methyl 3-hydroxytetradecanoate](https://cb.imsc.res.in/imppat/phytochemical-detailedpage/IMPHY016741) | CCCCCCCCCCCC(CC(=O)OC)O | [180523](https://pubchem.ncbi.nlm.nih.gov/compound/180523) |
| 200 | [3,5-Dimethylcyclohexanol](https://cb.imsc.res.in/imppat/phytochemical-detailedpage/IMPHY017208) | CC1CC(C)CC(C1)O | [21584](https://pubchem.ncbi.nlm.nih.gov/compound/21584) |
| 201 | [Butyl 3-methylhexanoate](https://cb.imsc.res.in/imppat/phytochemical-detailedpage/IMPHY017615) | CCCCOC(=O)CC(CCC)C | [6429068](https://pubchem.ncbi.nlm.nih.gov/compound/6429068) |
| 202 | [Ginkgetin](https://cb.imsc.res.in/imppat/phytochemical-detailedpage/IMPHY004546) | COc1ccc(cc1c1c(O)cc(c2c1oc(cc2=O)c1ccc(cc1)O)O)c1cc(=O)c2c(o1)cc(cc2O)OC | [5271805](https://pubchem.ncbi.nlm.nih.gov/compound/5271805) |
| 203 | [Sciadopitysin](https://cb.imsc.res.in/imppat/phytochemical-detailedpage/IMPHY005475) | COc1ccc(cc1)c1cc(=O)c2c(o1)c(c(cc2O)O)c1cc(ccc1OC)c1cc(=O)c2c(o1)cc(cc2O)OC | [5281696](https://pubchem.ncbi.nlm.nih.gov/compound/5281696) |
| 204 | [Amentoflavone](https://cb.imsc.res.in/imppat/phytochemical-detailedpage/IMPHY005599) | Oc1ccc(cc1)c1cc(=O)c2c(o1)c(c(cc2O)O)c1cc(ccc1O)c1cc(=O)c2c(o1)cc(cc2O)O | [5281600](https://pubchem.ncbi.nlm.nih.gov/compound/5281600) |
| 205 | [Sequoiaflavone](https://cb.imsc.res.in/imppat/phytochemical-detailedpage/IMPHY005862) | COc1cc(O)c2c(c1)oc(cc2=O)c1ccc(c(c1)c1c(O)cc(c2c1oc(cc2=O)c1ccc(cc1)O)O)O | [5484010](https://pubchem.ncbi.nlm.nih.gov/compound/5484010) |
| 206 | [beta-Eudesmol](https://cb.imsc.res.in/imppat/phytochemical-detailedpage/IMPHY011542) | C=C1CCC[C@]2([C@H]1C[C@@H](CC2)C(O)(C)C)C | [91457](https://pubchem.ncbi.nlm.nih.gov/compound/91457) |
| 207 | [alpha-Muurolene](https://cb.imsc.res.in/imppat/phytochemical-detailedpage/IMPHY011659) | CC1=C[C@@H]2[C@H](CC1)C(=CC[C@H]2C(C)C)C | [12306047](https://pubchem.ncbi.nlm.nih.gov/compound/12306047) |
| 208 | [gamma-Muurolene](https://cb.imsc.res.in/imppat/phytochemical-detailedpage/IMPHY011792) | CC1=C[C@@H]2[C@H](CC1)C(=C)CC[C@H]2C(C)C | [12313020](https://pubchem.ncbi.nlm.nih.gov/compound/12313020) |
| 209 | [(+)-gamma-Cadinene](https://cb.imsc.res.in/imppat/phytochemical-detailedpage/IMPHY011793) | CC1=C[C@@H]2[C@@H](CC1)C(=C)CC[C@H]2C(C)C | [6432404](https://pubchem.ncbi.nlm.nih.gov/compound/6432404) |
| 210 | [(-)-Globulol](https://cb.imsc.res.in/imppat/phytochemical-detailedpage/IMPHY014690) | C[C@@H]1CC[C@@H]2[C@@H]1[C@H]1[C@H](C1(C)C)CC[C@@]2(C)O | [12304985](https://pubchem.ncbi.nlm.nih.gov/compound/12304985) |
| 211 | [Cedrelanol](https://cb.imsc.res.in/imppat/phytochemical-detailedpage/IMPHY014906) | CC1=C[C@@H]2[C@@H](CC1)[C@@](C)(O)CC[C@H]2C(C)C | [160799](https://pubchem.ncbi.nlm.nih.gov/compound/160799) |
| 212 | [Ligstroside](https://cb.imsc.res.in/imppat/phytochemical-detailedpage/IMPHY001691) | OC[C@H]1O[C@@H](O[C@@H]2OC=C([C@H](/C/2=CC)CC(=O)OCCc2ccc(cc2)O)C(=O)OC)[C@@H]([C@H]([C@@H]1O)O)O | [14136859](https://pubchem.ncbi.nlm.nih.gov/compound/14136859) |
| 213 | [Dehydropodophyllotoxin](https://cb.imsc.res.in/imppat/phytochemical-detailedpage/IMPHY004993) | COc1cc(cc(c1OC)OC)c1c2C(=O)OCc2c(c2c1cc1OCOc1c2)O | [5316463](https://pubchem.ncbi.nlm.nih.gov/compound/5316463) |
| 214 | [methyl (4S,5Z,6S)-4-[2-[2-(3,4-dihydroxyphenyl)ethoxy]-2-oxoethyl]-5-ethylidene-6-[(2S,3R,4S,5S,6R)-3,4,5-trihydroxy-6-(hydroxymethyl)oxan-2-yl]oxy-4H-pyran-3-carboxylate](https://cb.imsc.res.in/imppat/phytochemical-detailedpage/IMPHY006819) | OC[C@H]1O[C@@H](OC2OC=C(C(/C/2=C/C)CC(=O)OCCc2ccc(c(c2)O)O)C(=O)OC)[C@@H]([C@H]([C@@H]1O)O)O | [24721401](https://pubchem.ncbi.nlm.nih.gov/compound/24721401) |
| 215 | [4-hydroxy-4-[2-[(2S,3S,4R,5R,6S)-3,4,5-trihydroxy-6-(hydroxymethyl)oxan-2-yl]oxyethyl]cyclohexa-2,5-dien-1-one](https://cb.imsc.res.in/imppat/phytochemical-detailedpage/IMPHY003089) | OC[C@@H]1O[C@H](OCCC2(O)C=CC(=O)C=C2)[C@H]([C@@H]([C@H]1O)O)O | [3084796](https://pubchem.ncbi.nlm.nih.gov/compound/3084796) |
| 216 | [Acteoside](https://cb.imsc.res.in/imppat/phytochemical-detailedpage/IMPHY003780) | OC[C@H]1O[C@@H](OCCc2ccc(c(c2)O)O)[C@@H]([C@H]([C@@H]1OC(=O)/C=C/c1ccc(c(c1)O)O)O[C@@H]1O[C@@H](C)[C@@H]([C@H]([C@H]1O)O)O)O | [5281800](https://pubchem.ncbi.nlm.nih.gov/compound/5281800) |
| 217 | [Maslinic acid](https://cb.imsc.res.in/imppat/phytochemical-detailedpage/IMPHY011970) | O[C@@H]1C[C@@]2(C)[C@H](C([C@H]1O)(C)C)CC[C@@]1([C@@H]2CC=C2[C@@]1(C)CC[C@@]1([C@H]2CC(C)(C)CC1)C(=O)O)C | [73659](https://pubchem.ncbi.nlm.nih.gov/compound/73659) |
| 218 | [Erythrodiol](https://cb.imsc.res.in/imppat/phytochemical-detailedpage/IMPHY002190) | OC[C@]12CC[C@@]3(C(=CC[C@H]4[C@@]3(C)CC[C@@H]3[C@]4(C)CC[C@@H](C3(C)C)O)[C@@H]2CC(CC1)(C)C)C | [101761](https://pubchem.ncbi.nlm.nih.gov/compound/101761) |
| 219 | [Cinchonidine](https://cb.imsc.res.in/imppat/phytochemical-detailedpage/IMPHY004204) | C=C[C@H]1CN2CC[C@H]1C[C@H]2[C@@H](c1ccnc2c1cccc2)O | [101744](https://pubchem.ncbi.nlm.nih.gov/compound/101744) |
| 220 | [Luteolin](https://cb.imsc.res.in/imppat/phytochemical-detailedpage/IMPHY004660) | Oc1cc(O)c2c(c1)oc(cc2=O)c1ccc(c(c1)O)O | [5280445](https://pubchem.ncbi.nlm.nih.gov/compound/5280445) |
| 221 | [Apigenin](https://cb.imsc.res.in/imppat/phytochemical-detailedpage/IMPHY004661) | Oc1ccc(cc1)c1cc(=O)c2c(o1)cc(cc2O)O | [5280443](https://pubchem.ncbi.nlm.nih.gov/compound/5280443) |
| 222 | [methyl (2S,4S)-4-[2-[2-(3,4-dihydroxyphenyl)ethoxy]-2-oxoethyl]-3-ethenyl-2-[(2S,3R,4S,5S,6R)-3,4,5-trihydroxy-6-(hydroxymethyl)oxan-2-yl]oxy-3,4-dihydro-2H-pyran-5-carboxylate](https://cb.imsc.res.in/imppat/phytochemical-detailedpage/IMPHY006833) | C=CC1[C@@H](OC=C([C@H]1CC(=O)OCCc1ccc(c(c1)O)O)C(=O)OC)O[C@@H]1O[C@H](CO)[C@H]([C@@H]([C@H]1O)O)O | [102016333](https://pubchem.ncbi.nlm.nih.gov/compound/102016333) |
| 223 | [Uvaol](https://cb.imsc.res.in/imppat/phytochemical-detailedpage/IMPHY007428) | OC[C@@]12CC[C@H]([C@@H]([C@H]2C2=CC[C@H]3[C@@]([C@@]2(CC1)C)(C)CC[C@@H]1[C@]3(C)CC[C@@H](C1(C)C)O)C)C | [92802](https://pubchem.ncbi.nlm.nih.gov/compound/92802) |
| 224 | [Oleuropeic acid](https://cb.imsc.res.in/imppat/phytochemical-detailedpage/IMPHY009047) | OC(=O)C1=CC[C@H](CC1)C(O)(C)C | [188320](https://pubchem.ncbi.nlm.nih.gov/compound/188320) |
| 225 | [Cosmosiin](https://cb.imsc.res.in/imppat/phytochemical-detailedpage/IMPHY012719) | OC[C@H]1O[C@@H](Oc2cc(O)c3c(c2)oc(cc3=O)c2ccc(cc2)O)[C@@H]([C@H]([C@@H]1O)O)O | [5280704](https://pubchem.ncbi.nlm.nih.gov/compound/5280704) |
| 226 | [alpha-Amyrin](https://cb.imsc.res.in/imppat/phytochemical-detailedpage/IMPHY011619) | C[C@@H]1CC[C@]2([C@@H]([C@H]1C)C1=CC[C@H]3[C@@]([C@@]1(CC2)C)(C)CC[C@@H]1[C@]3(C)CC[C@@H](C1(C)C)O)C | [73170](https://pubchem.ncbi.nlm.nih.gov/compound/73170) |
| 227 | [Betulinic acid](https://cb.imsc.res.in/imppat/phytochemical-detailedpage/IMPHY012003) | CC(=C)[C@@H]1CC[C@]2([C@H]1[C@H]1CC[C@H]3[C@@]([C@]1(C)CC2)(C)CC[C@@H]1[C@]3(C)CC[C@@H](C1(C)C)O)C(=O)O | [64971](https://pubchem.ncbi.nlm.nih.gov/compound/64971) |
| 228 | [p-Cresol](https://cb.imsc.res.in/imppat/phytochemical-detailedpage/IMPHY003113) | Cc1ccc(cc1)O | [2879](https://pubchem.ncbi.nlm.nih.gov/compound/2879) |
| 229 | [Citronellyl acetate](https://cb.imsc.res.in/imppat/phytochemical-detailedpage/IMPHY007421) | CC(CCC=C(C)C)CCOC(=O)C | [9017](https://pubchem.ncbi.nlm.nih.gov/compound/9017) |
| 230 | [Acetone](https://cb.imsc.res.in/imppat/phytochemical-detailedpage/IMPHY012019) | CC(=O)C | [180](https://pubchem.ncbi.nlm.nih.gov/compound/180) |
| 231 | [(E)-Hexadec-11-enal](https://cb.imsc.res.in/imppat/phytochemical-detailedpage/IMPHY012765) | CCCC/C=C/CCCCCCCCCC=O | [5283376](https://pubchem.ncbi.nlm.nih.gov/compound/5283376) |
| 232 | [Z-(13,14-Epoxy)tetradec-11-en-1-ol acetate](https://cb.imsc.res.in/imppat/phytochemical-detailedpage/IMPHY012818) | CC(=O)OCCCCCCCCCC/C=CC1OC1 | [5363633](https://pubchem.ncbi.nlm.nih.gov/compound/5363633) |
| 233 | [Hydantoin](https://cb.imsc.res.in/imppat/phytochemical-detailedpage/IMPHY013762) | O=C1NCC(=O)N1 | [10006](https://pubchem.ncbi.nlm.nih.gov/compound/10006) |
| 234 | [Kaempferol](https://cb.imsc.res.in/imppat/phytochemical-detailedpage/IMPHY004388) | Oc1ccc(cc1)c1oc2cc(O)cc(c2c(=O)c1O)O | [5280863](https://pubchem.ncbi.nlm.nih.gov/compound/5280863) |
| 235 | [Esculin](https://cb.imsc.res.in/imppat/phytochemical-detailedpage/IMPHY005620) | OC[C@H]1O[C@@H](Oc2cc3ccc(=O)oc3cc2O)[C@@H]([C@H]([C@@H]1O)O)O | [5281417](https://pubchem.ncbi.nlm.nih.gov/compound/5281417) |
| 236 | [Esculetin](https://cb.imsc.res.in/imppat/phytochemical-detailedpage/IMPHY011518) | O=c1ccc2c(o1)cc(c(c2)O)O | [5281416](https://pubchem.ncbi.nlm.nih.gov/compound/5281416) |
| 237 | [Oleoside](https://cb.imsc.res.in/imppat/phytochemical-detailedpage/IMPHY000389) | C/C=C1/[C@@H](OC=C([C@H]1CC(=O)O)C(=O)O)O[C@@H]1O[C@H](CO)[C@H]([C@@H]([C@H]1O)O)O | [101042548](https://pubchem.ncbi.nlm.nih.gov/compound/101042548) |
| 238 | [(4S,5E,6S)-4-[2-[2-(3,4-dihydroxyphenyl)ethoxy]-2-oxoethyl]-5-ethylidene-6-[(2S,3R,4S,5S,6R)-3,4,5-trihydroxy-6-(hydroxymethyl)oxan-2-yl]oxy-4H-pyran-3-carboxylic acid](https://cb.imsc.res.in/imppat/phytochemical-detailedpage/IMPHY007905) | OC[C@H]1O[C@@H](O[C@@H]2OC=C([C@H](/C/2=CC)CC(=O)OCCc2ccc(c(c2)O)O)C(=O)O)[C@@H]([C@H]([C@@H]1O)O)O | [6450302](https://pubchem.ncbi.nlm.nih.gov/compound/6450302) |
| 239 | [Alpha-d-xylopyranose](https://cb.imsc.res.in/imppat/phytochemical-detailedpage/IMPHY014807) | O[C@@H]1CO[C@@H]([C@@H]([C@H]1O)O)O | [6027](https://pubchem.ncbi.nlm.nih.gov/compound/6027) |
| 240 | isovitexin | C1=CC(=CC=C1C2=CC(=O)C3=C(O2)C=C(C(=C3O)C4C(C(C(C(O4)CO)O)O)O)O)O | 162350 |
| 241 | apigenin C-hexoside-C-pentoside |  |  |
| 242 | vitexin | C1=CC(=CC=C1C2=CC(=O)C3=C(O2)C(=C(C=C3O)O)C4C(C(C(C(O4)CO)O)O)O)O | 5280441 |
| 243 | kaempferol 3-O-rutinoside | CC1C(C(C(C(O1)OCC2C(C(C(C(O2)OC3=C(OC4=CC(=CC(=C4C3=O)O)O)C5=CC=C(C=C5)O)O)O)O)O)O)O | 5318767 |
| 244 | kaempferol | C1=CC(=CC=C1C2=C(C(=O)C3=C(C=C(C=C3O2)O)O)O)O | 5280863 |
| 245 | kaempferol 3-O-(6”-acetyl-galactoside)-7-O-rhamnoside | CC1C(C(C(C(O1)OC2=CC(=C3C(=C2)OC(=C(C3=O)OC4C(C(C(C(O4)COC(=O)C)O)O)O)C5=CC=C(C=C5)O)O)O)O)O | 102157736 |
| 246 | 4-hydroxybenzaldehyde | C1=CC(=CC=C1C=O)O | 126 |
| 247 | p-anisaldehyde | COC1=CC=C(C=C1)C=O | 31244 |
| 248 | p-coumaric acid | C1=CC(=CC=C1/C=C/C(=O)O)O | 637542 |
| 249 | Quinic acid | C1C(C(C(CC1(C(=O)O)O)O)O)O | 6508 |
| 250 | Cinnamic acid | C1=CC=C(C=C1)C=CC(=O)O | 444539 |
| 251 | Ferulic acid | COC1=C(C=CC(=C1)C=CC(=O)O)O | 445858 |
| 252 | Caffeic acid | C1=CC(=C(C=C1C=CC(=O)O)O)O | 689043 |
| 253 | Caffeic O-glucoside acid | C1=CC(=C(C=C1C=CC(=O)O)OC2C(C(C(C(O2)CO)O)O)O)O | 5281759 |
| 254 | Vanillin | COC1=C(C=CC(=C1)C=O)O | 1183 |
| 255 | Coniferin | COC1=C(C=CC(=C1)C=CCO)OC2C(C(C(C(O2)CO)O)O)O | 5280372 |
| 256 | Pheophorbide-a | CCC1=C(C2=NC1=CC3=C(C4=C(C(C(=C5C(C(C(=CC6=NC(=C2)C(=C6C)C=C)N5)C)CCC(=O)O)C4=N3)C(=O)OC)O)C)C | 167186 |
| 257 | pyropheophorbide-a | CCC1=C(C2=NC1=CC3=C(C4=C(CC(=C5C(C(C(=CC6=NC(=C2)C(=C6C)C=C)N5)C)CCC(=O)O)C4=N3)O)C)C | 161456 |
| 258 | methyl pyropheophorbide-a | CCC1=C(C2=NC1=CC3=C(C4=C(CC(=C5C(C(C(=CC6=NC(=C2)C(=C6C)C=C)N5)C)CCC(=O)OC)C4=N3)O)C)C | 135406598 |
| 259 | Oleic acid | CCCCCCCCC=CCCCCCCCC(=O)O | 445639 |
| 260 | Linoleic acid | CCCCCC=CCC=CCCCCCCCC(=O)O | 5280450 |
| 261 | Linolenic acid | CCC=CCC=CCC=CCCCCCCCC(=O)O | 5280934 |
| 262 | Campesterol | CC(C)C(C)CCC(C)C1CCC2C1(CCC3C2CC=C4C3(CCC(C4)O)C)C | 173183 |
| 263 | Stigmasterol | CCC(C=CC(C)C1CCC2C1(CCC3C2CC=C4C3(CCC(C4)O)C)C)C(C)C | 5280794 |
| 264 | Glucuronolactone | C(=O)C(C1C(C(C(=O)O1)O)O)O | 92283 |
| 265 | Apigenin | C1=CC(=CC=C1C2=CC(=O)C3=C(C=C(C=C3O2)O)O)O | 5280443 |
| 266 | luteolin | C1=CC(=C(C=C1C2=CC(=O)C3=C(C=C(C=C3O2)O)O)O)O | 5280445 |
| 267 | Vernolide | CC(=C)C(=O)OC1CC23C(O2)CCC(=CC4C1C(=C)C(=O)O4)COC3O | 5281508 |
| 268 | Hydroxyvernolide | C=C1C2C(CC34C(O3)CCC(=CC2OC1=O)COC4O)OC(=O)C(=C)CO | 5281472 |
| 269 | Vernodalol | COC(=O)C(=C)C1C(CC2(COC(=O)C(=C)C2C1O)C=C)OC(=O)C(=C)CO | 442318 |
| 270 | Vernomygdin | CC(C)C(=O)OC1CC23C(O2)CCC(=CC4C1C(=C)C(=O)O4)COC3O | 5281509 |
| 271 | 4-methylumbelliferone | CC1=CC(=O)OC2=C1C=CC(=C2)O | 5280567 |
| 272 | Cryptolepine | CN1C2=CC=CC=C2C=C3C1=C4C=CC=CC4=N3 | 82143 |
| 273 | Neocryptolepine | CN1C=C2C3=CC=CC=C3N=C2C4=CC=CC=C41 | 380925 |
| 274 | Vernomenin | C=CC12CC3C(C(C1C(=C)C(=O)OC2)O)C(=C)C(=O)O3 | 442324 |
| 275 | Maytansine | COc1cc2cc(c1Cl)N(C)C(=O)C[C@H](OC(=O)[C@H](C)N(C)C(C)=O)[C@]1(C)O[C@H]1[C@H](C)[C@@H]1C[C@@](O)(N=C(O)O1)[C@H](OC)/C=C/C=C(\C)C2 | 5281828 |
| 276 | [(1S,2S,4S,5R,6R,7S,9R,12R)-4,5,12-triacetyloxy-6-(acetyloxymethyl)-2-hydroxy-2,10,10-trimethyl-11-oxatricyclo[7.2.1.01,6]dodecan-7-yl] pyridine-3-carboxylate | CC(=O)OC[C@]12[C@@H](OC(=O)c3cccnc3)C[C@@H]3[C@@H](OC(C)=O)[C@]1(OC3(C)C)[C@@](C)(O)C[C@H](OC(C)=O)[C@@H]2OC(C)=O | 101324728 |
| 277 | [(1S,2S,4S,5R,6R,7S,9R,12R)-4,5,12-triacetyloxy-6-(acetyloxymethyl)-2-hydroxy-2,10,10-trimethyl-11-oxatricyclo[7.2.1.01,6]dodecan-7-yl] pyridine-3-carboxylate | CC(=O)OCC12C(OC(=O)c3cccnc3)CC3C(OC(C)=O)C1(OC3(C)C)C(C)(O)C(O)C(OC(C)=O)C2OC(C)=O | 15560167 |
| 278 | [(1S,2S,4S,5R,6R,7S,9R,12R)-4,5,12-triacetyloxy-6-(acetyloxymethyl)-2-hydroxy-2,10,10-trimethyl-11-oxatricyclo[7.2.1.01,6]dodecan-7-yl] pyridine-3-carboxylate | CC(=O)OC[C@]12[C@@H](OC(=O)c3cccnc3)C[C@@H]3[C@@H](OC(=O)c4ccccc4)[C@]1(OC3(C)C)[C@@](C)(O)[C@@H](OC(C)=O)[C@H](OC(C)=O)[C@@H]2OC(C)=O | 163070124 |
| 279 | [(1S,2S,4S,5R,6R,7S,9R,12R)-4,5,12-triacetyloxy-6-(acetyloxymethyl)-2-hydroxy-2,10,10-trimethyl-11-oxatricyclo[7.2.1.01,6]dodecan-7-yl] pyridine-3-carboxylate | CC(=O)OC[C@@]12[C@@H](OC(=O)c3cccnc3)C[C@H]3[C@@H](OC(C)=O)[C@@]1(OC3(C)C)[C@](C)(O)[C@@H](O)[C@H](OC(C)=O)[C@H]2OC(C)=O | 162973827 |
| 280 | [4,5,12-Triacetyloxy-6-(acetyloxymethyl)-2-hydroxy-2,10,10-trimethyl-11-oxatricyclo[7.2.1.01,6]dodecan-7-yl] pyridine-3-carboxylate | CC(=O)OCC12C(CC3C(C1(C(CC(C2OC(=O)C)OC(=O)C)(C)O)OC3(C)C)OC(=O)C)OC(=O)C4=CN=CC=C4 | 15560165 |
| 281 | [(1R,2S,3R,4S,5S,6R,7S,9S,12R)-3,4,5-triacetyloxy-6-(acetyloxymethyl)-12-benzoyloxy-2-hydroxy-2,10,10-trimethyl-11-oxatricyclo[7.2.1.01,6]dodecan-7-yl] pyridine-3-carboxylate | CC(=O)OC[C@]12[C@@H](OC(=O)c3cccnc3)C[C@H]3[C@@H](OC(=O)c4ccccc4)[C@@]1(OC3(C)C)[C@@](C)(O)[C@H](OC(C)=O)[C@@H](OC(C)=O)[C@H]2OC(C)=O | 163070123 |
| 282 | [(1R,2S,3R,4S,5S,6R,7S,9S,12R)-3,4,5-triacetyloxy-6-(acetyloxymethyl)-12-benzoyloxy-2-hydroxy-2,10,10-trimethyl-11-oxatricyclo[7.2.1.01,6]dodecan-7-yl] pyridine-3-carboxylate | CC(=O)OC[C@]12[C@@H](OC(=O)c3cccnc3)C[C@@H]3[C@@H](OC(C)=O)[C@]1(OC3(C)C)[C@@](C)(O)[C@@H](O)[C@H](OC(C)=O)[C@@H]2OC(C)=O | 101324727 |
| 283 | Rohitukine | Cc1cc(=O)c2c(O)cc(O)c([C@H]3CCN(C)C[C@H]3O)c2o1 | 13422573 |
| 284 | [(5R,6S,7S,8R,9R,10R,13R,17R)-7-acetyloxy-17-(furan-3-yl)-4,4,8,10,13-pentamethyl-3-oxo-5,6,7,9,11,12,16,17-octahydrocyclopenta[a]phenanthren-6-yl] acetate | CC(=O)OC1C2C(C(=O)C=CC2(C3CCC4(C(CC=C4C3(C1OC(=O)C)C)C5=COC=C5)C)C)(C)C | 162964716 |
| 285 | [3-Octanol](https://cb.imsc.res.in/imppat/phytochemical-detailedpage/IMPHY001828) | CCCCCC(CC)O | [11527](https://pubchem.ncbi.nlm.nih.gov/compound/11527) |
| 286 | [Dimethyl disulfide](https://cb.imsc.res.in/imppat/phytochemical-detailedpage/IMPHY001846) | CSSC | [12232](https://pubchem.ncbi.nlm.nih.gov/compound/12232) |
| 287 | [1-Penten-3-OL](https://cb.imsc.res.in/imppat/phytochemical-detailedpage/IMPHY001971) | CCC(C=C)O | [12020](https://pubchem.ncbi.nlm.nih.gov/compound/12020) |
| 288 | [2-Methylbutyl acetate](https://cb.imsc.res.in/imppat/phytochemical-detailedpage/IMPHY002069) | CCC(COC(=O)C)C | [12209](https://pubchem.ncbi.nlm.nih.gov/compound/12209) |
| 289 | [3-Octanone](https://cb.imsc.res.in/imppat/phytochemical-detailedpage/IMPHY003179) | CCCCCC(=O)CC | [246728](https://pubchem.ncbi.nlm.nih.gov/compound/246728) |
| 290 | [Acetoin](https://cb.imsc.res.in/imppat/phytochemical-detailedpage/IMPHY003340) | CC(=O)C(O)C | [179](https://pubchem.ncbi.nlm.nih.gov/compound/179) |
| 291 | [(1S,6R,7R)-1-methyl-3-methylidene-8-propan-2-yltricyclo[4.4.0.02,7]decane](https://cb.imsc.res.in/imppat/phytochemical-detailedpage/IMPHY003720) | CC(C1CC[C@]2([C@H]3[C@@H]1C2C(=C)CC3)C)C | [25244198](https://pubchem.ncbi.nlm.nih.gov/compound/25244198) |
| 292 | [(-)-beta-Bourbonene](https://cb.imsc.res.in/imppat/phytochemical-detailedpage/IMPHY003977) | CC([C@@H]1CC[C@@]2([C@H]1[C@H]1C(=C)CC[C@@H]21)C)C | [62566](https://pubchem.ncbi.nlm.nih.gov/compound/62566) |
| 293 | [Tiglic aldehyde](https://cb.imsc.res.in/imppat/phytochemical-detailedpage/IMPHY004934) | C/C=C(/C=O)C | [5321950](https://pubchem.ncbi.nlm.nih.gov/compound/5321950) |
| 294 | [1-Octen-3-yl acetate](https://cb.imsc.res.in/imppat/phytochemical-detailedpage/IMPHY005390) | CCCCCC(OC(=O)C)C=C | [17121](https://pubchem.ncbi.nlm.nih.gov/compound/17121) |
| 295 | [(E)-4,8-Dimethyl-1,3,7-nonatriene](https://cb.imsc.res.in/imppat/phytochemical-detailedpage/IMPHY006269) | C=C/C=C(/CCC=C(C)C)C | [6427110](https://pubchem.ncbi.nlm.nih.gov/compound/6427110) |
| 296 | [Ethyl isobutyrate](https://cb.imsc.res.in/imppat/phytochemical-detailedpage/IMPHY007007) | CCOC(=O)C(C)C | [7342](https://pubchem.ncbi.nlm.nih.gov/compound/7342) |
| 297 | [Isobutyl acetate](https://cb.imsc.res.in/imppat/phytochemical-detailedpage/IMPHY007219) | CC(COC(=O)C)C | [8038](https://pubchem.ncbi.nlm.nih.gov/compound/8038) |
| 298 | [2-Butanol](https://cb.imsc.res.in/imppat/phytochemical-detailedpage/IMPHY007710) | CCC(O)C | [6568](https://pubchem.ncbi.nlm.nih.gov/compound/6568) |
| 299 | [Eucalyptol](https://cb.imsc.res.in/imppat/phytochemical-detailedpage/IMPHY010072) | CC12CCC(CC1)C(O2)(C)C | [2758](https://pubchem.ncbi.nlm.nih.gov/compound/2758) |
| 300 | [3-Carene](https://cb.imsc.res.in/imppat/phytochemical-detailedpage/IMPHY011392) | CC1=CCC2C(C1)C2(C)C | [26049](https://pubchem.ncbi.nlm.nih.gov/compound/26049) |
| 301 | [Cadina-1,4-diene](https://cb.imsc.res.in/imppat/phytochemical-detailedpage/IMPHY011455) | CC1=CC2C(=CC1)[C@@H](C)CCC2C(C)C | [6427091](https://pubchem.ncbi.nlm.nih.gov/compound/6427091) |
| 302 | [(1R)-2-methyl-5-propan-2-ylbicyclo[3.1.0]hex-2-ene](https://cb.imsc.res.in/imppat/phytochemical-detailedpage/IMPHY011552) | CC1=CCC2([C@@H]1C2)C(C)C | [6451618](https://pubchem.ncbi.nlm.nih.gov/compound/6451618) |
| 303 | [(+)-alpha-Cadinene](https://cb.imsc.res.in/imppat/phytochemical-detailedpage/IMPHY011660) | CC1=C[C@@H]2[C@@H](CC1)C(=CC[C@H]2C(C)C)C | [12306048](https://pubchem.ncbi.nlm.nih.gov/compound/12306048) |
| 304 | [Citral](https://cb.imsc.res.in/imppat/phytochemical-detailedpage/IMPHY011789) | O=C/C=C(/CCC=C(C)C)C | [638011](https://pubchem.ncbi.nlm.nih.gov/compound/638011) |
| 305 | [cis-3-Hexenyl acetate](https://cb.imsc.res.in/imppat/phytochemical-detailedpage/IMPHY011804) | CC/C=CCCOC(=O)C | [5363388](https://pubchem.ncbi.nlm.nih.gov/compound/5363388) |
| 306 | [Sec-butyl acetate](https://cb.imsc.res.in/imppat/phytochemical-detailedpage/IMPHY012084) | CCC(OC(=O)C)C | [7758](https://pubchem.ncbi.nlm.nih.gov/compound/7758) |
| 307 | [(1S,2S,6S,7R,8R)-1,3-dimethyl-8-propan-2-yltricyclo[4.4.0.02,7]dec-3-ene](https://cb.imsc.res.in/imppat/phytochemical-detailedpage/IMPHY012168) | CC([C@H]1CC[C@]2([C@@H]3[C@@H]1[C@H]2C(=CC3)C)C)C | [101607926](https://pubchem.ncbi.nlm.nih.gov/compound/101607926) |
| 308 | [Calamenene](https://cb.imsc.res.in/imppat/phytochemical-detailedpage/IMPHY014865) | CC([C@@H]1CC[C@@H](c2c1cc(C)cc2)C)C | [6429077](https://pubchem.ncbi.nlm.nih.gov/compound/6429077) |
| 309 | [3-Hexanol](https://cb.imsc.res.in/imppat/phytochemical-detailedpage/IMPHY015730) | CCCC(CC)O | [12178](https://pubchem.ncbi.nlm.nih.gov/compound/12178) |
| 310 | [2-(Methylamino)benzaldehyde](https://cb.imsc.res.in/imppat/phytochemical-detailedpage/IMPHY016217) | O=Cc1ccccc1NC | [267569](https://pubchem.ncbi.nlm.nih.gov/compound/267569) |
| 311 | [Methyl 2-methoxybenzoate](https://cb.imsc.res.in/imppat/phytochemical-detailedpage/IMPHY016737) | COC(=O)c1ccccc1OC | [61151](https://pubchem.ncbi.nlm.nih.gov/compound/61151) |
| 312 | [Baicalin](https://cb.imsc.res.in/imppat/phytochemical-detailedpage/IMPHY004115) | OC(=O)[C@H]1O[C@@H](Oc2cc3oc(cc(=O)c3c(c2O)O)c2ccccc2)[C@@H]([C@H]([C@@H]1O)O)O | [64982](https://pubchem.ncbi.nlm.nih.gov/compound/64982) |
| 313 | [Skullcapflavone I](https://cb.imsc.res.in/imppat/phytochemical-detailedpage/IMPHY004375) | COc1cc(O)c2c(c1OC)oc(cc2=O)c1ccccc1O | [5320399](https://pubchem.ncbi.nlm.nih.gov/compound/5320399) |
| 314 | [Wogonin](https://cb.imsc.res.in/imppat/phytochemical-detailedpage/IMPHY005530) | COc1c(O)cc(c2c1oc(cc2=O)c1ccccc1)O | [5281703](https://pubchem.ncbi.nlm.nih.gov/compound/5281703) |
| 315 | [Baicalein](https://cb.imsc.res.in/imppat/phytochemical-detailedpage/IMPHY005607) | Oc1cc2oc(cc(=O)c2c(c1O)O)c1ccccc1 | [5281605](https://pubchem.ncbi.nlm.nih.gov/compound/5281605) |
| 316 | [Skullcapflavone II](https://cb.imsc.res.in/imppat/phytochemical-detailedpage/IMPHY009523) | COc1cccc(c1c1cc(=O)c2c(o1)c(OC)c(c(c2O)OC)OC)O | [124211](https://pubchem.ncbi.nlm.nih.gov/compound/124211) |
| 317 | [alpha-Amyrenyl acetate](https://cb.imsc.res.in/imppat/phytochemical-detailedpage/IMPHY004141) | CC(=O)O[C@H]1CC[C@]2([C@H](C1(C)C)CC[C@@]1([C@@H]2CC=C2[C@@]1(C)CC[C@@]1([C@H]2[C@@H](C)[C@H](C)CC1)C)C)C | [92842](https://pubchem.ncbi.nlm.nih.gov/compound/92842) |
| 318 | [2,5-Dimethoxy-p-cymene](https://cb.imsc.res.in/imppat/phytochemical-detailedpage/IMPHY006243) | COc1cc(C(C)C)c(cc1C)OC | [6427071](https://pubchem.ncbi.nlm.nih.gov/compound/6427071) |
| 319 | [Thymol](https://cb.imsc.res.in/imppat/phytochemical-detailedpage/IMPHY006550) | Cc1ccc(c(c1)O)C(C)C | [6989](https://pubchem.ncbi.nlm.nih.gov/compound/6989) |
| 320 | [1-Hentriacontanol](https://cb.imsc.res.in/imppat/phytochemical-detailedpage/IMPHY007132) | CCCCCCCCCCCCCCCCCCCCCCCCCCCCCCCO | [68345](https://pubchem.ncbi.nlm.nih.gov/compound/68345) |
| 321 | [alpha-Terpinyl acetate](https://cb.imsc.res.in/imppat/phytochemical-detailedpage/IMPHY011519) | CC(=O)OC(C1CCC(=CC1)C)(C)C | [111037](https://pubchem.ncbi.nlm.nih.gov/compound/111037) |
| 322 | [(Z)-gamma-bisabolene](https://cb.imsc.res.in/imppat/phytochemical-detailedpage/IMPHY011839) | CC(=CCC/C(=C1/CCC(=CC1)C)/C)C | [3033866](https://pubchem.ncbi.nlm.nih.gov/compound/3033866) |
| 323 | [beta-Asarone](https://cb.imsc.res.in/imppat/phytochemical-detailedpage/IMPHY011991) | C/C=Cc1cc(OC)c(cc1OC)OC | [5281758](https://pubchem.ncbi.nlm.nih.gov/compound/5281758) |
| 324 | [Dihydrocarveol](https://cb.imsc.res.in/imppat/phytochemical-detailedpage/IMPHY012130) | CC(=C)C1CCC(C(C1)O)C | [12072](https://pubchem.ncbi.nlm.nih.gov/compound/12072) |
| 325 | [Campesterol](https://cb.imsc.res.in/imppat/phytochemical-detailedpage/IMPHY012402) | O[C@H]1CC[C@]2(C(=CC[C@@H]3[C@@H]2CC[C@]2([C@H]3CC[C@@H]2[C@@H](CC[C@H](C(C)C)C)C)C)C1)C | [173183](https://pubchem.ncbi.nlm.nih.gov/compound/173183) |
| 326 | [3-(1,5-Dimethyl-4-hexenyl)-6-methylene-1-cyclohexene](https://cb.imsc.res.in/imppat/phytochemical-detailedpage/IMPHY012589) | CC(C1CCC(=C)C=C1)CCC=C(C)C | [519764](https://pubchem.ncbi.nlm.nih.gov/compound/519764) |
| 327 | [Lupeol acetate](https://cb.imsc.res.in/imppat/phytochemical-detailedpage/IMPHY014991) | CC(=O)O[C@H]1CC[C@]2([C@H](C1(C)C)CC[C@@]1([C@@H]2CC[C@H]2[C@@]1(C)CC[C@@]1([C@@H]2[C@@H](CC1)C(=C)C)C)C)C | [92157](https://pubchem.ncbi.nlm.nih.gov/compound/92157) |
| 328 | [(-)-Zingiberene](https://cb.imsc.res.in/imppat/phytochemical-detailedpage/IMPHY015121) | CC(=CCCC(C1CC=C(C=C1)C)C)C | [521253](https://pubchem.ncbi.nlm.nih.gov/compound/521253) |
| 329 | [Beta-Muurolene](https://cb.imsc.res.in/imppat/phytochemical-detailedpage/IMPHY016901) | CC1=CC[C@H]2[C@@H](C1)[C@@H](CC=C2C)C(C)C | [25203009](https://pubchem.ncbi.nlm.nih.gov/compound/25203009) |
| 330 | [Coniferyl alcohol](https://cb.imsc.res.in/imppat/phytochemical-detailedpage/IMPHY001545) | OC/C=C/c1ccc(c(c1)OC)O | [1549095](https://pubchem.ncbi.nlm.nih.gov/compound/1549095) |
| 331 | [Thymol methyl ether](https://cb.imsc.res.in/imppat/phytochemical-detailedpage/IMPHY001658) | COc1cc(C)ccc1C(C)C | [14104](https://pubchem.ncbi.nlm.nih.gov/compound/14104) |
| 332 | [Artemetin](https://cb.imsc.res.in/imppat/phytochemical-detailedpage/IMPHY004407) | COc1ccc(cc1OC)c1oc2cc(OC)c(c(c2c(=O)c1OC)O)OC | [5320351](https://pubchem.ncbi.nlm.nih.gov/compound/5320351) |
| 333 | [(+)-Fenchone](https://cb.imsc.res.in/imppat/phytochemical-detailedpage/IMPHY013835) | O=C1[C@@]2(C)CC[C@@H](C1(C)C)C2 | [1201521](https://pubchem.ncbi.nlm.nih.gov/compound/1201521) |
| 334 | [beta-Himachalene](https://cb.imsc.res.in/imppat/phytochemical-detailedpage/IMPHY014833) | CC1=C[C@H]2C(=C(C)CCCC2(C)C)CC1 | [11586487](https://pubchem.ncbi.nlm.nih.gov/compound/11586487) |
| 335 | [beta Farnesene](https://cb.imsc.res.in/imppat/phytochemical-detailedpage/IMPHY017124) | CCC(=C)CC/C=C(/CCC=C(C)C)C | [15228937](https://pubchem.ncbi.nlm.nih.gov/compound/15228937) |
| 336 | [Camphor](https://cb.imsc.res.in/imppat/phytochemical-detailedpage/IMPHY012036) | O=C1CC2C(C1(C)CC2)(C)C | [2537](https://pubchem.ncbi.nlm.nih.gov/compound/2537) |
| 337 | [Hispidulin](https://cb.imsc.res.in/imppat/phytochemical-detailedpage/IMPHY005442) | COc1c(O)cc2c(c1O)c(=O)cc(o2)c1ccc(cc1)O | [5281628](https://pubchem.ncbi.nlm.nih.gov/compound/5281628) |
| 338 | [[(2S,4R,8S,9R,12R)-1,12-dihydroxy-2,11-dimethyl-7-methylidene-6-oxo-5,14-dioxatricyclo[9.2.1.04,8]tetradecan-9-yl] 2-methylpropanoate](https://cb.imsc.res.in/imppat/phytochemical-detailedpage/IMPHY010458) | O=C(C(C)C)O[C@@H]1CC2(C)OC(C[C@H]2O)([C@H](C[C@@H]2[C@@H]1C(=C)C(=O)O2)C)O | [102004628](https://pubchem.ncbi.nlm.nih.gov/compound/102004628) |
| 339 | [tagitinin C](https://cb.imsc.res.in/imppat/phytochemical-detailedpage/IMPHY011139) | O=C(C(C)C)O[C@@H]1C[C@@](C)(O)/C=C/C(=O)/C(=C[C@@H]2[C@@H]1C(=C)C(=O)O2)/C | [11256548](https://pubchem.ncbi.nlm.nih.gov/compound/11256548) |
| 340 | [Pinocarvone](https://cb.imsc.res.in/imppat/phytochemical-detailedpage/IMPHY002072) | C=C1C(=O)CC2CC1C2(C)C | [121719](https://pubchem.ncbi.nlm.nih.gov/compound/121719) |
| 341 | [Myrtenal](https://cb.imsc.res.in/imppat/phytochemical-detailedpage/IMPHY006325) | O=CC1=CCC2CC1C2(C)C | [61130](https://pubchem.ncbi.nlm.nih.gov/compound/61130) |
| 342 | [Cyclosativene](https://cb.imsc.res.in/imppat/phytochemical-detailedpage/IMPHY009840) | CC(C1CCC2(C3C1C1C(C21C)C3)C)C | [519960](https://pubchem.ncbi.nlm.nih.gov/compound/519960) |
| 343 | [p-Mentha-1,5-dien-8-ol](https://cb.imsc.res.in/imppat/phytochemical-detailedpage/IMPHY009880) | CC1=CCC(C=C1)C(O)(C)C | [519323](https://pubchem.ncbi.nlm.nih.gov/compound/519323) |
| 344 | [beta-Farnesene](https://cb.imsc.res.in/imppat/phytochemical-detailedpage/IMPHY011658) | C=CC(=C)CC/C=C(/CCC=C(C)C)C | [5281517](https://pubchem.ncbi.nlm.nih.gov/compound/5281517) |
| 345 | [(4E,7E)-1,5,9,9-Tetramethyl-12-oxabicyclo[9.1.0]dodeca-4,7-diene](https://cb.imsc.res.in/imppat/phytochemical-detailedpage/IMPHY011872) | C/C/1=C/CCC2(C)OC2CC(/C=CC1)(C)C | [22559443](https://pubchem.ncbi.nlm.nih.gov/compound/22559443) |
| 346 | [Citronellal](https://cb.imsc.res.in/imppat/phytochemical-detailedpage/IMPHY012086) | O=CCC(CCC=C(C)C)C | [7794](https://pubchem.ncbi.nlm.nih.gov/compound/7794) |
| 347 | [(1r,3s,5r)-6,6-Dimethyl-2-methylidenebicyclo[3.1.1]heptan-3-ol](https://cb.imsc.res.in/imppat/phytochemical-detailedpage/IMPHY012265) | C=C1[C@@H](O)C[C@H]2C[C@@H]1C2(C)C | [88302](https://pubchem.ncbi.nlm.nih.gov/compound/88302) |
| 348 | [2-Furanmethanol, 5-ethenyltetrahydro-alpha,alpha,5-trimethyl-, cis-](https://cb.imsc.res.in/imppat/phytochemical-detailedpage/IMPHY012920) | C=C[C@@]1(C)CC[C@H](O1)C(O)(C)C | [11116492](https://pubchem.ncbi.nlm.nih.gov/compound/11116492) |
| 349 | [gamma-Elemene](https://cb.imsc.res.in/imppat/phytochemical-detailedpage/IMPHY012921) | C=C[C@]1(C)CCC(=C(C)C)C[C@H]1C(=C)C | [6432312](https://pubchem.ncbi.nlm.nih.gov/compound/6432312) |
| 350 | [cis-Nerolidol](https://cb.imsc.res.in/imppat/phytochemical-detailedpage/IMPHY014871) | C=CC(CC/C=C(CCC=C(C)C)/C)(O)C | [5320128](https://pubchem.ncbi.nlm.nih.gov/compound/5320128) |
| 351 | [trans-Linalool oxide](https://cb.imsc.res.in/imppat/phytochemical-detailedpage/IMPHY014989) | C=C[C@]1(C)CC[C@H](O1)C(O)(C)C | [6432254](https://pubchem.ncbi.nlm.nih.gov/compound/6432254) |
| 352 | [trans-Verbenol](https://cb.imsc.res.in/imppat/phytochemical-detailedpage/IMPHY015098) | CC1=C[C@H](O)[C@@H]2C[C@H]1C2(C)C | [89664](https://pubchem.ncbi.nlm.nih.gov/compound/89664) |
| 353 | [Cyclotagitinin C](https://cb.imsc.res.in/imppat/phytochemical-detailedpage/IMPHY010769) | O=C(C(C)C)O[C@@H]1C[C@@](C)(O)[C@H]2C(=C(C(=O)C2)C)[C@@H]2[C@@H]1C(=C)C(=O)O2 | no id |
| 354 | [Tagitinin E](https://cb.imsc.res.in/imppat/phytochemical-detailedpage/IMPHY011140) | O=C(C(C)C)O[C@@H]1C[C@@]2(C)O[C@@H]2C[C@@H](/C(=C[C@@H]2[C@@H]1C(=C)C(=O)O2)/C)O | [101967037](https://pubchem.ncbi.nlm.nih.gov/compound/101967037) |
| 355 | [Tagitinin F](https://cb.imsc.res.in/imppat/phytochemical-detailedpage/IMPHY011141) | O=C(C(C)C)O[C@@H]1C[C@]2(C)C=C[C@@](O2)(/C(=C[C@@H]2[C@@H]1C(=C)C(=O)O2)/C)O | [5281501](https://pubchem.ncbi.nlm.nih.gov/compound/5281501) |
| 356 | [Tirotundin](https://cb.imsc.res.in/imppat/phytochemical-detailedpage/IMPHY012980) | O=C(C(C)C)O[C@@H]1C[C@]2(C)CC[C@@](O2)([C@H](C[C@@H]2[C@@H]1C(=C)C(=O)O2)C)O | [9975297](https://pubchem.ncbi.nlm.nih.gov/compound/9975297) |
| 357 | [Okanin 3'-glucoside](https://cb.imsc.res.in/imppat/phytochemical-detailedpage/IMPHY002799) | OCC1OC(Oc2c(O)ccc(c2O)C(=O)/C=C/c2ccc(c(c2)O)O)C(C(C1O)O)O | [14213549](https://pubchem.ncbi.nlm.nih.gov/compound/14213549) |
| 358 | [1-Hexadecanol](https://cb.imsc.res.in/imppat/phytochemical-detailedpage/IMPHY002983) | CCCCCCCCCCCCCCCCO | [2682](https://pubchem.ncbi.nlm.nih.gov/compound/2682) |
| 359 | [Pimara-8(14),15-diene](https://cb.imsc.res.in/imppat/phytochemical-detailedpage/IMPHY003459) | C=C[C@]1(C)CC[C@H]2C(=C1)CC[C@@H]1[C@]2(C)CCCC1(C)C | [440909](https://pubchem.ncbi.nlm.nih.gov/compound/440909) |
| 360 | [beta-Copaene](https://cb.imsc.res.in/imppat/phytochemical-detailedpage/IMPHY003719) | CC([C@@H]1CC[C@]2([C@@H]3[C@H]1C2C(=C)CC3)C)C | [57339298](https://pubchem.ncbi.nlm.nih.gov/compound/57339298) |
| 361 | [Phenylheptatriyne](https://cb.imsc.res.in/imppat/phytochemical-detailedpage/IMPHY005989) | CC#CC#CC#Cc1ccccc1 | [77981](https://pubchem.ncbi.nlm.nih.gov/compound/77981) |
| 362 | [Perillene](https://cb.imsc.res.in/imppat/phytochemical-detailedpage/IMPHY007131) | CC(=CCCc1cocc1)C | [68316](https://pubchem.ncbi.nlm.nih.gov/compound/68316) |
| 363 | [Hexadecyl acetate](https://cb.imsc.res.in/imppat/phytochemical-detailedpage/IMPHY009411) | CCCCCCCCCCCCCCCCOC(=O)C | [12393](https://pubchem.ncbi.nlm.nih.gov/compound/12393) |
| 364 | [cis-Chrysanthenyl acetate](https://cb.imsc.res.in/imppat/phytochemical-detailedpage/IMPHY011371) | CC(=O)OC1[C@H]2CC=C([C@@H]1C2(C)C)C | [6431301](https://pubchem.ncbi.nlm.nih.gov/compound/6431301) |
| 365 | [(-)-Epicedrol](https://cb.imsc.res.in/imppat/phytochemical-detailedpage/IMPHY012209) | C[C@@H]1CC[C@@H]2[C@@]31CC[C@]([C@H](C3)C2(C)C)(C)O | [6713078](https://pubchem.ncbi.nlm.nih.gov/compound/6713078) |
| 366 | [delta-Elemene](https://cb.imsc.res.in/imppat/phytochemical-detailedpage/IMPHY013093) | C=C[C@@]1(C)CCC(=C[C@@H]1C(=C)C)C(C)C | [12309449](https://pubchem.ncbi.nlm.nih.gov/compound/12309449) |
| 367 | [4-Allyl-2-methoxyphenyl isobutyrate](https://cb.imsc.res.in/imppat/phytochemical-detailedpage/IMPHY016659) | C=CCc1ccc(c(c1)OC)OC(=O)C(C)C | [3019983](https://pubchem.ncbi.nlm.nih.gov/compound/3019983) |
| 368 | [Octadecadienyl acetate](https://cb.imsc.res.in/imppat/phytochemical-detailedpage/IMPHY016785) | CCCCCCCCCCCCCC/C=C/C=C/OC(=O)C | [22292269](https://pubchem.ncbi.nlm.nih.gov/compound/22292269) |
| 369 | [1-Phenyl-5-heptene-1,3-diyne](https://cb.imsc.res.in/imppat/phytochemical-detailedpage/IMPHY017336) | C/C=C/C#CC#Cc1ccccc1 | [5281154](https://pubchem.ncbi.nlm.nih.gov/compound/5281154) |
| 370 | [Octadecadienol](https://cb.imsc.res.in/imppat/phytochemical-detailedpage/IMPHY017699) | CCCCCCCCCCCCCCC=CC=CO | [71317672](https://pubchem.ncbi.nlm.nih.gov/compound/71317672) |
| 371 | [7-Phenyl-2,4,6-heptatriyne-1-ol acetate](https://cb.imsc.res.in/imppat/phytochemical-detailedpage/IMPHY001888) | CC(=O)OCC#CC#CC#Cc1ccccc1 | [11644243](https://pubchem.ncbi.nlm.nih.gov/compound/11644243) |
| 372 | [Decanoic acid](https://cb.imsc.res.in/imppat/phytochemical-detailedpage/IMPHY003104) | CCCCCCCCCC(=O)O | [2969](https://pubchem.ncbi.nlm.nih.gov/compound/2969) |
| 373 | [7-Phenyl-2,4,6-heptatriyn-1-ol](https://cb.imsc.res.in/imppat/phytochemical-detailedpage/IMPHY003106) | OCC#CC#CC#Cc1ccccc1 | [3085176](https://pubchem.ncbi.nlm.nih.gov/compound/3085176) |
| 374 | [(5E)-1-Phenyl-5-heptene-1,3-diyn-7-ol acetate](https://cb.imsc.res.in/imppat/phytochemical-detailedpage/IMPHY005072) | CC(=O)OC/C=C/C#CC#Cc1ccccc1 | [23274467](https://pubchem.ncbi.nlm.nih.gov/compound/23274467) |
| 375 | [2-Acetylthiophene](https://cb.imsc.res.in/imppat/phytochemical-detailedpage/IMPHY006626) | CC(=O)c1cccs1 | [6920](https://pubchem.ncbi.nlm.nih.gov/compound/6920) |
| 376 | [Elaidic acid](https://cb.imsc.res.in/imppat/phytochemical-detailedpage/IMPHY011801) | CCCCCCCC/C=C/CCCCCCCC(=O)O | [637517](https://pubchem.ncbi.nlm.nih.gov/compound/637517) |
| 377 | [Epifriedelanol](https://cb.imsc.res.in/imppat/phytochemical-detailedpage/IMPHY011859) | O[C@H]1CC[C@@H]2[C@]([C@H]1C)(C)CC[C@H]1[C@@]2(C)CC[C@@]2([C@]1(C)CC[C@@]1([C@H]2CC(C)(C)CC1)C)C | [119242](https://pubchem.ncbi.nlm.nih.gov/compound/119242) |
| 378 | [Isoquercitrin](https://cb.imsc.res.in/imppat/phytochemical-detailedpage/IMPHY012721) | OC[C@H]1O[C@@H](Oc2c(oc3c(c2=O)c(O)cc(c3)O)c2ccc(c(c2)O)O)[C@@H]([C@H]([C@@H]1O)O)O | [5280804](https://pubchem.ncbi.nlm.nih.gov/compound/5280804) |
| 379 | [Stigmasterol](https://cb.imsc.res.in/imppat/phytochemical-detailedpage/IMPHY014842) | CC[C@@H](C(C)C)/C=C/[C@H]([C@H]1CC[C@@H]2[C@]1(C)CC[C@H]1[C@H]2CC=C2[C@]1(C)CC[C@@H](C2)O)C | [5280794](cid:5280794) |
| 380 | [Octanal](https://cb.imsc.res.in/imppat/phytochemical-detailedpage/IMPHY000795) | CCCCCCCC=O | [454](https://pubchem.ncbi.nlm.nih.gov/compound/454) |
| 381 | [Vomifoliol](https://cb.imsc.res.in/imppat/phytochemical-detailedpage/IMPHY004646) | C[C@H](/C=C/[C@@]1(O)C(=CC(=O)CC1(C)C)C)O | [5280462](https://pubchem.ncbi.nlm.nih.gov/compound/5280462) |
| 382 | [Methylparaben](https://cb.imsc.res.in/imppat/phytochemical-detailedpage/IMPHY006154) | COC(=O)c1ccc(cc1)O | [7456](https://pubchem.ncbi.nlm.nih.gov/compound/7456) |
| 383 | [Decanal](https://cb.imsc.res.in/imppat/phytochemical-detailedpage/IMPHY006970) | CCCCCCCCCC=O | [8175](https://pubchem.ncbi.nlm.nih.gov/compound/8175) |
| 384 | [Dodecanal](https://cb.imsc.res.in/imppat/phytochemical-detailedpage/IMPHY007204) | CCCCCCCCCCCC=O | [8194](https://pubchem.ncbi.nlm.nih.gov/compound/8194) |
| 385 | [2-Undecanone](https://cb.imsc.res.in/imppat/phytochemical-detailedpage/IMPHY011521) | CCCCCCCCCC(=O)C | [8163](https://pubchem.ncbi.nlm.nih.gov/compound/8163) |
| 386 | [Afzelin](https://cb.imsc.res.in/imppat/phytochemical-detailedpage/IMPHY011919) | Oc1ccc(cc1)c1oc2cc(O)cc(c2c(=O)c1O[C@@H]1O[C@@H](C)[C@@H]([C@H]([C@H]1O)O)O)O | [5316673](https://pubchem.ncbi.nlm.nih.gov/compound/5316673) |
| 387 | [1,3,5-Tridecanoylbenzene](https://cb.imsc.res.in/imppat/phytochemical-detailedpage/IMPHY014494) | CCCCCCCCCC(=O)c1cc(cc(c1)C(=O)CCCCCCCCC)C(=O)CCCCCCCCC | [86173717](https://pubchem.ncbi.nlm.nih.gov/compound/86173717) |
| 388 | [5-[3-(1,3-Benzodioxol-5-yl)-1,3,3a,4,6,6a-hexahydrofuro[3,4-c]furan-6-yl]-1,3-benzodioxole](https://cb.imsc.res.in/imppat/phytochemical-detailedpage/IMPHY014895) | C1Oc2c(O1)cc(cc2)C1OCC2C1COC2c1ccc2c(c1)OCO2 | [5204](https://pubchem.ncbi.nlm.nih.gov/compound/5204) |
| 389 | [Hyperoside](https://cb.imsc.res.in/imppat/phytochemical-detailedpage/IMPHY014935) | OC[C@H]1O[C@@H](Oc2c(oc3c(c2=O)c(O)cc(c3)O)c2ccc(c(c2)O)O)[C@@H]([C@H]([C@H]1O)O)O | [5281643](https://pubchem.ncbi.nlm.nih.gov/compound/5281643) |
| 390 | [Quercitrin](https://cb.imsc.res.in/imppat/phytochemical-detailedpage/IMPHY015054) | Oc1cc(O)c2c(c1)oc(c(c2=O)O[C@@H]1O[C@@H](C)[C@@H]([C@H]([C@H]1O)O)O)c1ccc(c(c1)O)O | [5280459](https://pubchem.ncbi.nlm.nih.gov/compound/5280459) |
| 391 | [Cepharadione B](https://cb.imsc.res.in/imppat/phytochemical-detailedpage/IMPHY003328) | COc1cc2C(=O)C(=O)N(c3c2c(c1OC)c1ccccc1c3)C | [189151](https://pubchem.ncbi.nlm.nih.gov/compound/189151) |
| 392 | [Norcepharadione B](https://cb.imsc.res.in/imppat/phytochemical-detailedpage/IMPHY003329) | COc1cc2C(=O)C(=O)Nc3c2c(c1OC)c1ccccc1c3 | [189168](https://pubchem.ncbi.nlm.nih.gov/compound/189168) |
| 393 | [Piperolactam A](https://cb.imsc.res.in/imppat/phytochemical-detailedpage/IMPHY012679) | COc1cc2C(=O)Nc3c2c(c1O)c1ccccc1c3 | [3081016](https://pubchem.ncbi.nlm.nih.gov/compound/3081016) |
| 394 | [7-Chloro-6-demethylcepharadione B](https://cb.imsc.res.in/imppat/phytochemical-detailedpage/IMPHY013711) | COc1cc2C(=O)C(=O)Nc3c2c(c1OC)c1ccccc1c3Cl | [131752718](https://pubchem.ncbi.nlm.nih.gov/compound/131752718) |
| 395 | [1,4-Dihydropyridine](https://cb.imsc.res.in/imppat/phytochemical-detailedpage/IMPHY000598) | C1C=CNC=C1 | [104822](https://pubchem.ncbi.nlm.nih.gov/compound/104822) |
| 396 | [Decanoyl acetaldehyde](https://cb.imsc.res.in/imppat/phytochemical-detailedpage/IMPHY002075) | CCCCCCCCCC(=O)CC=O | [122640](https://pubchem.ncbi.nlm.nih.gov/compound/122640) |
| 397 | [Aristolactam BII](https://cb.imsc.res.in/imppat/phytochemical-detailedpage/IMPHY005360) | COc1c(OC)cc2c3c1c1ccccc1cc3NC2=O | [162739](https://pubchem.ncbi.nlm.nih.gov/compound/162739) |
| 398 | [Rosamultin](https://cb.imsc.res.in/imppat/phytochemical-detailedpage/IMPHY005505) | OC[C@H]1O[C@@H](OC(=O)[C@@]23CC[C@H]([C@@]([C@H]3C3=CC[C@H]4[C@@]([C@@]3(CC2)C)(C)CC[C@@H]2[C@]4(C)C[C@H]([C@@H](C2(C)C)O)O)(C)O)C)[C@@H]([C@H]([C@@H]1O)O)O | [21122581](https://pubchem.ncbi.nlm.nih.gov/compound/21122581) |
| 399 | [Methyl gallate](https://cb.imsc.res.in/imppat/phytochemical-detailedpage/IMPHY006139) | COC(=O)c1cc(O)c(c(c1)O)O | [7428](https://pubchem.ncbi.nlm.nih.gov/compound/7428) |
| 400 | [1-Decanol](https://cb.imsc.res.in/imppat/phytochemical-detailedpage/IMPHY006947) | CCCCCCCCCCO | [8174](https://pubchem.ncbi.nlm.nih.gov/compound/8174) |
| 401 | [1-Dodecanol](https://cb.imsc.res.in/imppat/phytochemical-detailedpage/IMPHY007100) | CCCCCCCCCCCCO | [8193](https://pubchem.ncbi.nlm.nih.gov/compound/8193) |
| 402 | [Nonan-1-ol](https://cb.imsc.res.in/imppat/phytochemical-detailedpage/IMPHY007276) | CCCCCCCCCO | [8914](https://pubchem.ncbi.nlm.nih.gov/compound/8914) |
| 403 | Procyanidin B3 | Oc1cc(O)c2c(c1)O[C@@H]([C@H]([C@@H]2c1c(O)cc(c2c1O[C@@H]([C@H](C2)O)c1ccc(c(c1)O)O)O)O)c1ccc(c(c1)O)O | 146798 |
| 404 | D-Limonene | CC1=CC[C@@H](CC1)C(=C)C | 440917 |
| 405 | [Aristolactam AII](https://cb.imsc.res.in/imppat/phytochemical-detailedpage/IMPHY012353) | COc1c(O)cc2c3c1c1ccccc1cc3NC2=O | [148657](https://pubchem.ncbi.nlm.nih.gov/compound/148657) |
| 406 | [Nerol](https://cb.imsc.res.in/imppat/phytochemical-detailedpage/IMPHY012654) | OC/C=C(CCC=C(C)C)/C | [643820](https://pubchem.ncbi.nlm.nih.gov/compound/643820) |
| 407 | [Quercetin-3-glucoside](https://cb.imsc.res.in/imppat/phytochemical-detailedpage/IMPHY014396) | OC[C@H]1O[C@@H](Oc2c(oc3c(c2=O)c(O)cc(c3)O)c2ccc(c(c2)O)[O-])[C@@H]([C@H]([C@@H]1O)O)O | [25203368](https://pubchem.ncbi.nlm.nih.gov/compound/25203368) |
| 408 | [3-Methyl-2-buten-1-OL](https://cb.imsc.res.in/imppat/phytochemical-detailedpage/IMPHY000037) | OCC=C(C)C | [11173](https://pubchem.ncbi.nlm.nih.gov/compound/11173) |
| 409 | [Hexadecane](https://cb.imsc.res.in/imppat/phytochemical-detailedpage/IMPHY000308) | CCCCCCCCCCCCCCCC | [11006](https://pubchem.ncbi.nlm.nih.gov/compound/11006) |
| 410 | [Propanol](https://cb.imsc.res.in/imppat/phytochemical-detailedpage/IMPHY001215) | CCCO | [1031](https://pubchem.ncbi.nlm.nih.gov/compound/1031) |
| 411 | [Ethyl vinyl ketone](https://cb.imsc.res.in/imppat/phytochemical-detailedpage/IMPHY001577) | CCC(=O)C=C | [15394](https://pubchem.ncbi.nlm.nih.gov/compound/15394) |
| 412 | [3-Methylbutanal](https://cb.imsc.res.in/imppat/phytochemical-detailedpage/IMPHY001854) | O=CCC(C)C | [11552](https://pubchem.ncbi.nlm.nih.gov/compound/11552) |
| 413 | [Heptacosane](https://cb.imsc.res.in/imppat/phytochemical-detailedpage/IMPHY001896) | CCCCCCCCCCCCCCCCCCCCCCCCCCC | [11636](https://pubchem.ncbi.nlm.nih.gov/compound/11636) |
| 414 | [Octadecane](https://cb.imsc.res.in/imppat/phytochemical-detailedpage/IMPHY001915) | CCCCCCCCCCCCCCCCCC | [11635](https://pubchem.ncbi.nlm.nih.gov/compound/11635) |
| 415 | [2-(4-Methylphenyl)propan-2-ol](https://cb.imsc.res.in/imppat/phytochemical-detailedpage/IMPHY002825) | Cc1ccc(cc1)C(O)(C)C | [14529](https://pubchem.ncbi.nlm.nih.gov/compound/14529) |
| 416 | [Benzyl Alcohol](https://cb.imsc.res.in/imppat/phytochemical-detailedpage/IMPHY002915) | OCc1ccccc1 | [244](https://pubchem.ncbi.nlm.nih.gov/compound/244) |
| 417 | [1-Butanol](https://cb.imsc.res.in/imppat/phytochemical-detailedpage/IMPHY002949) | CCCCO | [263](https://pubchem.ncbi.nlm.nih.gov/compound/263) |
| 418 | [2,3,5-Trimethylpyrazine](https://cb.imsc.res.in/imppat/phytochemical-detailedpage/IMPHY002958) | Cc1cnc(c(n1)C)C | [26808](https://pubchem.ncbi.nlm.nih.gov/compound/26808) |
| 419 | [Octanoic acid](https://cb.imsc.res.in/imppat/phytochemical-detailedpage/IMPHY003301) | CCCCCCCC(=O)O | [379](https://pubchem.ncbi.nlm.nih.gov/compound/379) |
| 420 | [Acetylacetone](https://cb.imsc.res.in/imppat/phytochemical-detailedpage/IMPHY003476) | CC(=O)CC(=O)C | [31261](https://pubchem.ncbi.nlm.nih.gov/compound/31261) |
| 421 | [Isoamyl alcohol](https://cb.imsc.res.in/imppat/phytochemical-detailedpage/IMPHY003513) | OCCC(C)C | [31260](https://pubchem.ncbi.nlm.nih.gov/compound/31260) |
| 422 | [Methyl tetradecanoate](https://cb.imsc.res.in/imppat/phytochemical-detailedpage/IMPHY003518) | CCCCCCCCCCCCCC(=O)OC | [31284](https://pubchem.ncbi.nlm.nih.gov/compound/31284) |
| 423 | [Nonanal](https://cb.imsc.res.in/imppat/phytochemical-detailedpage/IMPHY003525) | CCCCCCCCC=O | [31289](https://pubchem.ncbi.nlm.nih.gov/compound/31289) |
| 424 | [Eugenol](https://cb.imsc.res.in/imppat/phytochemical-detailedpage/IMPHY003536) | C=CCc1ccc(c(c1)OC)O | [3314](https://pubchem.ncbi.nlm.nih.gov/compound/3314) |
| 425 | [2-Nonenal](https://cb.imsc.res.in/imppat/phytochemical-detailedpage/IMPHY003760) | CCCCCC/C=C/C=O | [5283335](https://pubchem.ncbi.nlm.nih.gov/compound/5283335) |
| 426 | [2-Decenal](https://cb.imsc.res.in/imppat/phytochemical-detailedpage/IMPHY003915) | CCCCCCC/C=C/C=O | [5283345](https://pubchem.ncbi.nlm.nih.gov/compound/5283345) |
| 427 | [(1S,2R,5S)-2-isopropyl-5-methylcyclohexyl acetate](https://cb.imsc.res.in/imppat/phytochemical-detailedpage/IMPHY004194) | C[C@H]1CC[C@@H]([C@H](C1)OC(=O)C)C(C)C | [62335](https://pubchem.ncbi.nlm.nih.gov/compound/62335) |
| 428 | [Methyl linoleate](https://cb.imsc.res.in/imppat/phytochemical-detailedpage/IMPHY004225) | CCCCC/C=CC/C=CCCCCCCCC(=O)OC | [5284421](https://pubchem.ncbi.nlm.nih.gov/compound/5284421) |
| 429 | [Methyl linolenate](https://cb.imsc.res.in/imppat/phytochemical-detailedpage/IMPHY004399) | CC/C=CC/C=CC/C=CCCCCCCCC(=O)OC | [5319706](https://pubchem.ncbi.nlm.nih.gov/compound/5319706) |
| 430 | [1,3-p-Menthadien-7-al](https://cb.imsc.res.in/imppat/phytochemical-detailedpage/IMPHY004528) | O=CC1=CC=C(CC1)C(C)C | [526762](https://pubchem.ncbi.nlm.nih.gov/compound/526762) |
| 431 | [3-(Methylthio)propionaldehyde](https://cb.imsc.res.in/imppat/phytochemical-detailedpage/IMPHY004751) | CSCCC=O | [18635](https://pubchem.ncbi.nlm.nih.gov/compound/18635) |
| 432 | [Syringin](https://cb.imsc.res.in/imppat/phytochemical-detailedpage/IMPHY004977) | OC/C=C/c1cc(OC)c(c(c1)OC)O[C@@H]1O[C@H](CO)[C@H]([C@@H]([C@H]1O)O)O | [5316860](https://pubchem.ncbi.nlm.nih.gov/compound/5316860) |
| 433 | [1-Octen-3-OL](https://cb.imsc.res.in/imppat/phytochemical-detailedpage/IMPHY005345) | CCCCCC(C=C)O | [18827](https://pubchem.ncbi.nlm.nih.gov/compound/18827) |
| 434 | [2-Undecenal](https://cb.imsc.res.in/imppat/phytochemical-detailedpage/IMPHY005526) | CCCCCCCC/C=C/C=O | [5283356](https://pubchem.ncbi.nlm.nih.gov/compound/5283356) |
| 435 | [2-Octenal](https://cb.imsc.res.in/imppat/phytochemical-detailedpage/IMPHY005549) | CCCCC/C=C/C=O | [5283324](https://pubchem.ncbi.nlm.nih.gov/compound/5283324) |
| 436 | [2-Octanol](https://cb.imsc.res.in/imppat/phytochemical-detailedpage/IMPHY005653) | CCCCCCC(O)C | [20083](https://pubchem.ncbi.nlm.nih.gov/compound/20083) |
| 437 | [trans-2-Pentenal](https://cb.imsc.res.in/imppat/phytochemical-detailedpage/IMPHY005935) | CC/C=C/C=O | [5364752](https://pubchem.ncbi.nlm.nih.gov/compound/5364752) |
| 438 | [Methyl oleate](https://cb.imsc.res.in/imppat/phytochemical-detailedpage/IMPHY005950) | CCCCCCCC/C=CCCCCCCCC(=O)OC | [5364509](https://pubchem.ncbi.nlm.nih.gov/compound/5364509) |
| 439 | [3-Octen-2-one](https://cb.imsc.res.in/imppat/phytochemical-detailedpage/IMPHY005994) | CCCC/C=C/C(=O)C | [5363229](https://pubchem.ncbi.nlm.nih.gov/compound/5363229) |
| 440 | [4-Methylbenzaldehyde](https://cb.imsc.res.in/imppat/phytochemical-detailedpage/IMPHY006026) | O=Cc1ccc(cc1)C | [7725](https://pubchem.ncbi.nlm.nih.gov/compound/7725) |
| 441 | [Geranyllinalool](https://cb.imsc.res.in/imppat/phytochemical-detailedpage/IMPHY006176) | C=CC(CC/C=C(/CC/C=C(/CCC=C(C)C)C)C)(O)C | [5365872](https://pubchem.ncbi.nlm.nih.gov/compound/5365872) |
| 442 | [2-Phenylethanol](https://cb.imsc.res.in/imppat/phytochemical-detailedpage/IMPHY006279) | OCCc1ccccc1 | [6054](https://pubchem.ncbi.nlm.nih.gov/compound/6054) |
| 443 | [Hexanal](https://cb.imsc.res.in/imppat/phytochemical-detailedpage/IMPHY006347) | CCCCCC=O | [6184](https://pubchem.ncbi.nlm.nih.gov/compound/6184) |
| 444 | [Damascenone](https://cb.imsc.res.in/imppat/phytochemical-detailedpage/IMPHY006404) | C/C=C/C(=O)C1=C(C)C=CCC1(C)C | [5366074](https://pubchem.ncbi.nlm.nih.gov/compound/5366074) |
| 445 | [Ethyl linolenate](https://cb.imsc.res.in/imppat/phytochemical-detailedpage/IMPHY006410) | CC/C=CC/C=CC/C=CCCCCCCCC(=O)OCC | [5367460](https://pubchem.ncbi.nlm.nih.gov/compound/5367460) |
| 446 | [beta-Ionone](https://cb.imsc.res.in/imppat/phytochemical-detailedpage/IMPHY006485) | CC(=O)/C=C/C1=C(C)CCCC1(C)C | [638014](https://pubchem.ncbi.nlm.nih.gov/compound/638014) |
| 447 | [2-Methyl-2-buten-1-OL](https://cb.imsc.res.in/imppat/phytochemical-detailedpage/IMPHY006565) | C/C(=CC)/CO | [6433417](https://pubchem.ncbi.nlm.nih.gov/compound/6433417) |
| 448 | [1,2-Dimethoxybenzene](https://cb.imsc.res.in/imppat/phytochemical-detailedpage/IMPHY006840) | COc1ccccc1OC | [7043](https://pubchem.ncbi.nlm.nih.gov/compound/7043) |
| 449 | [Isobutyric acid](https://cb.imsc.res.in/imppat/phytochemical-detailedpage/IMPHY006907) | CC(C(=O)O)C | [6590](https://pubchem.ncbi.nlm.nih.gov/compound/6590) |
| 450 | [Eicosane](https://cb.imsc.res.in/imppat/phytochemical-detailedpage/IMPHY006951) | CCCCCCCCCCCCCCCCCCCC | [8222](https://pubchem.ncbi.nlm.nih.gov/compound/8222) |
| 451 | [alpha,alpha-Dimethyl-4-methylenecyclohexanemethanol](https://cb.imsc.res.in/imppat/phytochemical-detailedpage/IMPHY006965) | CC(C1CCC(=C)CC1)(O)C | [81722](https://pubchem.ncbi.nlm.nih.gov/compound/81722) |
| 452 | [Indole](https://cb.imsc.res.in/imppat/phytochemical-detailedpage/IMPHY006981) | c1ccc2c(c1)[nH]cc2 | [798](https://pubchem.ncbi.nlm.nih.gov/compound/798) |
| 453 | [Pentane](https://cb.imsc.res.in/imppat/phytochemical-detailedpage/IMPHY006982) | CCCCC | [8003](https://pubchem.ncbi.nlm.nih.gov/compound/8003) |
| 454 | [Furfuryl alcohol](https://cb.imsc.res.in/imppat/phytochemical-detailedpage/IMPHY007006) | OCc1ccco1 | [7361](https://pubchem.ncbi.nlm.nih.gov/compound/7361) |
| 455 | [Acetophenone](https://cb.imsc.res.in/imppat/phytochemical-detailedpage/IMPHY007039) | CC(=O)c1ccccc1 | [7410](https://pubchem.ncbi.nlm.nih.gov/compound/7410) |
| 456 | [Benzyl salicylate](https://cb.imsc.res.in/imppat/phytochemical-detailedpage/IMPHY007048) | O=C(c1ccccc1O)OCc1ccccc1 | [8363](https://pubchem.ncbi.nlm.nih.gov/compound/8363) |
| 457 | [Undecanoic acid](https://cb.imsc.res.in/imppat/phytochemical-detailedpage/IMPHY007068) | CCCCCCCCCCC(=O)O | [8180](https://pubchem.ncbi.nlm.nih.gov/compound/8180) |
| 458 | [2-Methyl-3-buten-2-OL](https://cb.imsc.res.in/imppat/phytochemical-detailedpage/IMPHY007162) | C=CC(O)(C)C | [8257](https://pubchem.ncbi.nlm.nih.gov/compound/8257) |
| 459 | [Heptanal](https://cb.imsc.res.in/imppat/phytochemical-detailedpage/IMPHY007186) | CCCCCCC=O | [8130](https://pubchem.ncbi.nlm.nih.gov/compound/8130) |
| 460 | [Ethyl stearate](https://cb.imsc.res.in/imppat/phytochemical-detailedpage/IMPHY007193) | CCCCCCCCCCCCCCCCCC(=O)OCC | [8122](https://pubchem.ncbi.nlm.nih.gov/compound/8122) |
| 461 | [Heptanoic acid](https://cb.imsc.res.in/imppat/phytochemical-detailedpage/IMPHY007195) | CCCCCCC(=O)O | [8094](https://pubchem.ncbi.nlm.nih.gov/compound/8094) |
| 462 | [Methyl stearate](https://cb.imsc.res.in/imppat/phytochemical-detailedpage/IMPHY007213) | CCCCCCCCCCCCCCCCCC(=O)OC | [8201](https://pubchem.ncbi.nlm.nih.gov/compound/8201) |
| 463 | [Methyl decanoate](https://cb.imsc.res.in/imppat/phytochemical-detailedpage/IMPHY007221) | CCCCCCCCCC(=O)OC | [8050](https://pubchem.ncbi.nlm.nih.gov/compound/8050) |
| 464 | [Pentanal](https://cb.imsc.res.in/imppat/phytochemical-detailedpage/IMPHY007222) | CCCCC=O | [8063](https://pubchem.ncbi.nlm.nih.gov/compound/8063) |
| 465 | [1-Hexacosanol](https://cb.imsc.res.in/imppat/phytochemical-detailedpage/IMPHY007273) | CCCCCCCCCCCCCCCCCCCCCCCCCCO | [68171](https://pubchem.ncbi.nlm.nih.gov/compound/68171) |
| 466 | [1,4-Dimethoxybenzene](https://cb.imsc.res.in/imppat/phytochemical-detailedpage/IMPHY007431) | COc1ccc(cc1)OC | [9016](https://pubchem.ncbi.nlm.nih.gov/compound/9016) |
| 467 | [1-Octanol](https://cb.imsc.res.in/imppat/phytochemical-detailedpage/IMPHY007620) | CCCCCCCCO | [957](https://pubchem.ncbi.nlm.nih.gov/compound/957) |
| 468 | [1-Pentanol](https://cb.imsc.res.in/imppat/phytochemical-detailedpage/IMPHY008146) | CCCCCO | [6276](https://pubchem.ncbi.nlm.nih.gov/compound/6276) |
| 469 | [Inositol](https://cb.imsc.res.in/imppat/phytochemical-detailedpage/IMPHY008252) | OC1C(O)C(O)C(C(C1O)O)O | [892](https://pubchem.ncbi.nlm.nih.gov/compound/892) |
| 470 | [Furfuryl acetate](https://cb.imsc.res.in/imppat/phytochemical-detailedpage/IMPHY008508) | CC(=O)OCc1ccco1 | [12170](https://pubchem.ncbi.nlm.nih.gov/compound/12170) |
| 471 | [gamma-Octalactone](https://cb.imsc.res.in/imppat/phytochemical-detailedpage/IMPHY008880) | CCCCC1CCC(=O)O1 | [7704](https://pubchem.ncbi.nlm.nih.gov/compound/7704) |
| 472 | [Tetracosane](https://cb.imsc.res.in/imppat/phytochemical-detailedpage/IMPHY009355) | CCCCCCCCCCCCCCCCCCCCCCCC | [12592](https://pubchem.ncbi.nlm.nih.gov/compound/12592) |
| 473 | [Hexacosane](https://cb.imsc.res.in/imppat/phytochemical-detailedpage/IMPHY009359) | CCCCCCCCCCCCCCCCCCCCCCCCCC | [12407](https://pubchem.ncbi.nlm.nih.gov/compound/12407) |
| 474 | [Heptadecane](https://cb.imsc.res.in/imppat/phytochemical-detailedpage/IMPHY009368) | CCCCCCCCCCCCCCCCC | [12398](https://pubchem.ncbi.nlm.nih.gov/compound/12398) |
| 475 | [Nonadecane](https://cb.imsc.res.in/imppat/phytochemical-detailedpage/IMPHY009369) | CCCCCCCCCCCCCCCCCCC | [12401](https://pubchem.ncbi.nlm.nih.gov/compound/12401) |
| 476 | [Docosane](https://cb.imsc.res.in/imppat/phytochemical-detailedpage/IMPHY009375) | CCCCCCCCCCCCCCCCCCCCCC | [12405](https://pubchem.ncbi.nlm.nih.gov/compound/12405) |
| 477 | [Pentacosane](https://cb.imsc.res.in/imppat/phytochemical-detailedpage/IMPHY009377) | CCCCCCCCCCCCCCCCCCCCCCCCC | [12406](https://pubchem.ncbi.nlm.nih.gov/compound/12406) |
| 478 | [Heneicosane](https://cb.imsc.res.in/imppat/phytochemical-detailedpage/IMPHY009382) | CCCCCCCCCCCCCCCCCCCCC | [12403](https://pubchem.ncbi.nlm.nih.gov/compound/12403) |
| 479 | [Pentadecane](https://cb.imsc.res.in/imppat/phytochemical-detailedpage/IMPHY009389) | CCCCCCCCCCCCCCC | [12391](https://pubchem.ncbi.nlm.nih.gov/compound/12391) |
| 480 | [Tridecane](https://cb.imsc.res.in/imppat/phytochemical-detailedpage/IMPHY009419) | CCCCCCCCCCCCC | [12388](https://pubchem.ncbi.nlm.nih.gov/compound/12388) |
| 481 | [2-Decanone](https://cb.imsc.res.in/imppat/phytochemical-detailedpage/IMPHY009460) | CCCCCCCCC(=O)C | [12741](https://pubchem.ncbi.nlm.nih.gov/compound/12741) |
| 482 | [gamma-Decalactone](https://cb.imsc.res.in/imppat/phytochemical-detailedpage/IMPHY009467) | CCCCCCC1CCC(=O)O1 | [12813](https://pubchem.ncbi.nlm.nih.gov/compound/12813) |
| 483 | [Tricosane](https://cb.imsc.res.in/imppat/phytochemical-detailedpage/IMPHY009490) | CCCCCCCCCCCCCCCCCCCCCCC | [12534](https://pubchem.ncbi.nlm.nih.gov/compound/12534) |
| 484 | [Tridecanoic acid](https://cb.imsc.res.in/imppat/phytochemical-detailedpage/IMPHY009513) | CCCCCCCCCCCCC(=O)O | [12530](https://pubchem.ncbi.nlm.nih.gov/compound/12530) |
| 485 | [Pentyl acetate](https://cb.imsc.res.in/imppat/phytochemical-detailedpage/IMPHY009596) | CCCCCOC(=O)C | [12348](https://pubchem.ncbi.nlm.nih.gov/compound/12348) |
| 486 | [Ethyl palmitate](https://cb.imsc.res.in/imppat/phytochemical-detailedpage/IMPHY009624) | CCCCCCCCCCCCCCCC(=O)OCC | [12366](https://pubchem.ncbi.nlm.nih.gov/compound/12366) |
| 487 | [1-Heptanol](https://cb.imsc.res.in/imppat/phytochemical-detailedpage/IMPHY009751) | CCCCCCCO | [8129](https://pubchem.ncbi.nlm.nih.gov/compound/8129) |
| 488 | [beta-Cyclocitral](https://cb.imsc.res.in/imppat/phytochemical-detailedpage/IMPHY009752) | O=CC1=C(C)CCCC1(C)C | [9895](https://pubchem.ncbi.nlm.nih.gov/compound/9895) |
| 489 | [Naphthalene](https://cb.imsc.res.in/imppat/phytochemical-detailedpage/IMPHY009853) | c1ccc2c(c1)cccc2 | [931](https://pubchem.ncbi.nlm.nih.gov/compound/931) |
| 490 | [Benzaldehyde](https://cb.imsc.res.in/imppat/phytochemical-detailedpage/IMPHY009946) | O=Cc1ccccc1 | [240](https://pubchem.ncbi.nlm.nih.gov/compound/240) |
| 491 | [Ethyl valerate](https://cb.imsc.res.in/imppat/phytochemical-detailedpage/IMPHY009973) | CCCCC(=O)OCC | [10882](https://pubchem.ncbi.nlm.nih.gov/compound/10882) |
| 492 | [Benzyl benzoate](https://cb.imsc.res.in/imppat/phytochemical-detailedpage/IMPHY010097) | O=C(c1ccccc1)OCc1ccccc1 | [2345](https://pubchem.ncbi.nlm.nih.gov/compound/2345) |
| 493 | [cis-3-Hexenoic acid](https://cb.imsc.res.in/imppat/phytochemical-detailedpage/IMPHY010296) | CC/C=CCC(=O)O | [5355152](https://pubchem.ncbi.nlm.nih.gov/compound/5355152) |
| 494 | [Ethyl linoleate](https://cb.imsc.res.in/imppat/phytochemical-detailedpage/IMPHY010841) | CCCCC/C=CC/C=CCCCCCCCC(=O)OCC | [5282184](https://pubchem.ncbi.nlm.nih.gov/compound/5282184) |
| 495 | [Toluene](https://cb.imsc.res.in/imppat/phytochemical-detailedpage/IMPHY010995) | Cc1ccccc1 | [1140](https://pubchem.ncbi.nlm.nih.gov/compound/1140) |
| 496 | [Methacrolein](https://cb.imsc.res.in/imppat/phytochemical-detailedpage/IMPHY011003) | CC(=C)C=O | [6562](https://pubchem.ncbi.nlm.nih.gov/compound/6562) |
| 497 | [Guaiacol](https://cb.imsc.res.in/imppat/phytochemical-detailedpage/IMPHY011409) | COc1ccccc1O | [460](https://pubchem.ncbi.nlm.nih.gov/compound/460) |
| 498 | [2-Hexenal](https://cb.imsc.res.in/imppat/phytochemical-detailedpage/IMPHY011562) | CCC/C=C/C=O | [5281168](https://pubchem.ncbi.nlm.nih.gov/compound/5281168) |
| 499 | [cis-3-Hexen-1-ol](https://cb.imsc.res.in/imppat/phytochemical-detailedpage/IMPHY011588) | OCC/C=CCC | [5281167](https://pubchem.ncbi.nlm.nih.gov/compound/5281167) |
| 500 | [Geranyl acetate](https://cb.imsc.res.in/imppat/phytochemical-detailedpage/IMPHY011647) | C/C(=CCOC(=O)C)/CCC=C(C)C | [1549026](https://pubchem.ncbi.nlm.nih.gov/compound/1549026) |
| 501 | [cis-beta-Farnesene](https://cb.imsc.res.in/imppat/phytochemical-detailedpage/IMPHY011657) | C=CC(=C)CC/C=C(CCC=C(C)C)/C | [5317319](https://pubchem.ncbi.nlm.nih.gov/compound/5317319) |
| 502 | [Sorbitol](https://cb.imsc.res.in/imppat/phytochemical-detailedpage/IMPHY011727) | OC[C@H]([C@H]([C@@H]([C@H](CO)O)O)O)O | [5780](https://pubchem.ncbi.nlm.nih.gov/compound/5780) |
| 503 | [Anethole](https://cb.imsc.res.in/imppat/phytochemical-detailedpage/IMPHY011763) | C/C=C/c1ccc(cc1)OC | [637563](https://pubchem.ncbi.nlm.nih.gov/compound/637563) |
| 504 | [1,2,3,4,5-Cyclohexanepentol](https://cb.imsc.res.in/imppat/phytochemical-detailedpage/IMPHY011795) | OC1CC(O)C(C(C1O)O)O | [101715](https://pubchem.ncbi.nlm.nih.gov/compound/101715) |
| 505 | [Quercitol](https://cb.imsc.res.in/imppat/phytochemical-detailedpage/IMPHY011805) | O[C@@H]1C[C@@H](O)[C@@H](C([C@H]1O)O)O | [441437](https://pubchem.ncbi.nlm.nih.gov/compound/441437) |
| 506 | [Humulene epoxide](https://cb.imsc.res.in/imppat/phytochemical-detailedpage/IMPHY011873) | C/C/1=CCCC2(C)OC2CC(/C=C/C1)(C)C | [5352470](https://pubchem.ncbi.nlm.nih.gov/compound/5352470) |
| 507 | [Pulegone](https://cb.imsc.res.in/imppat/phytochemical-detailedpage/IMPHY011884) | C[C@@H]1CCC(=C(C)C)C(=O)C1 | [442495](https://pubchem.ncbi.nlm.nih.gov/compound/442495) |
| 508 | [Thujone](https://cb.imsc.res.in/imppat/phytochemical-detailedpage/IMPHY011901) | O=C1C[C@]2([C@@H]([C@H]1C)C2)C(C)C | [261491](https://pubchem.ncbi.nlm.nih.gov/compound/261491) |
| 509 | [beta-Thujone](https://cb.imsc.res.in/imppat/phytochemical-detailedpage/IMPHY011902) | O=C1C[C@]2([C@@H]([C@@H]1C)C2)C(C)C | [91456](https://pubchem.ncbi.nlm.nih.gov/compound/91456) |
| 510 | [Propionic acid](https://cb.imsc.res.in/imppat/phytochemical-detailedpage/IMPHY012028) | CCC(=O)O | [1032](https://pubchem.ncbi.nlm.nih.gov/compound/1032) |
| 511 | [Gamma-nonalactone](https://cb.imsc.res.in/imppat/phytochemical-detailedpage/IMPHY012082) | CCCCCC1CCC(=O)O1 | [7710](https://pubchem.ncbi.nlm.nih.gov/compound/7710) |
| 512 | [Valeric acid](https://cb.imsc.res.in/imppat/phytochemical-detailedpage/IMPHY012089) | CCCCC(=O)O | [7991](https://pubchem.ncbi.nlm.nih.gov/compound/7991) |
| 513 | [p-Menthan-3-one](https://cb.imsc.res.in/imppat/phytochemical-detailedpage/IMPHY012178) | CC1CCC(C(=O)C1)C(C)C | [6986](https://pubchem.ncbi.nlm.nih.gov/compound/6986) |
| 514 | [alpha-Bergamotene](https://cb.imsc.res.in/imppat/phytochemical-detailedpage/IMPHY012261) | CC(=CCCC1(C)C2CC=C(C1C2)C)C | [86608](https://pubchem.ncbi.nlm.nih.gov/compound/86608) |
| 515 | [alpha-Curcumene](https://cb.imsc.res.in/imppat/phytochemical-detailedpage/IMPHY012279) | CC(=CCCC(c1ccc(cc1)C)C)C | [92139](https://pubchem.ncbi.nlm.nih.gov/compound/92139) |
| 516 | [1D-1-O-Methyl-myo-inositol](https://cb.imsc.res.in/imppat/phytochemical-detailedpage/IMPHY012355) | COC1[C@H](O)[C@H](O)C([C@@H]([C@H]1O)O)O | [440078](https://pubchem.ncbi.nlm.nih.gov/compound/440078) |
| 517 | [Narcissin](https://cb.imsc.res.in/imppat/phytochemical-detailedpage/IMPHY012868) | COc1cc(ccc1O)c1oc2cc(O)cc(c2c(=O)c1O[C@@H]1O[C@H](CO[C@@H]2O[C@@H](C)[C@@H]([C@H]([C@H]2O)O)O)[C@H]([C@@H]([C@H]1O)O)O)O | [5481663](https://pubchem.ncbi.nlm.nih.gov/compound/5481663) |
| 518 | [Viscumitol](https://cb.imsc.res.in/imppat/phytochemical-detailedpage/IMPHY013507) | CO[C@@H]1[C@H](OC)[C@@H](O)[C@@H]([C@@H]([C@H]1O)O)O | [57459394](https://pubchem.ncbi.nlm.nih.gov/compound/57459394) |
| 519 | [gamma-Cadinol](https://cb.imsc.res.in/imppat/phytochemical-detailedpage/IMPHY013577) | CC1=CCC2C(C1)[C@H](CC[C@]2(C)O)C(C)C | [91753503](https://pubchem.ncbi.nlm.nih.gov/compound/91753503) |
| 520 | [Menthone](https://cb.imsc.res.in/imppat/phytochemical-detailedpage/IMPHY015004) | C[C@@H]1CC[C@H](C(=O)C1)C(C)C | [26447](https://pubchem.ncbi.nlm.nih.gov/compound/26447) |
| 521 | [D-Pinitol](https://cb.imsc.res.in/imppat/phytochemical-detailedpage/IMPHY015039) | COC1[C@H](O)[C@@H](O)C([C@@H]([C@@H]1O)O)O | [164619](https://pubchem.ncbi.nlm.nih.gov/compound/164619) |
| 522 | [3-Nonen-2-one](https://cb.imsc.res.in/imppat/phytochemical-detailedpage/IMPHY015637) | CCCCC/C=C/C(=O)C | [5317045](https://pubchem.ncbi.nlm.nih.gov/compound/5317045) |
| 523 | [Linalool oxide acetate (pyranoid)](https://cb.imsc.res.in/imppat/phytochemical-detailedpage/IMPHY016041) | C=C[C@@]1(C)CC[C@@H](C(O1)(C)C)OC(=O)C | [6427501](https://pubchem.ncbi.nlm.nih.gov/compound/6427501) |
| 524 | [Viridiflorol](https://cb.imsc.res.in/imppat/phytochemical-detailedpage/IMPHY016053) | C[C@@H]1CC[C@H]2[C@@H]1[C@H]1[C@H](C1(C)C)CC[C@]2(C)O | [11996452](https://pubchem.ncbi.nlm.nih.gov/compound/11996452) |
| 525 | [5-Ethenyl-2,3-dimethylfuran](https://cb.imsc.res.in/imppat/phytochemical-detailedpage/IMPHY017143) | C=Cc1oc(c(c1)C)C | [15801237](https://pubchem.ncbi.nlm.nih.gov/compound/15801237) |
| 526 | [2-Heptenoic acid](https://cb.imsc.res.in/imppat/phytochemical-detailedpage/IMPHY017337) | CCCC/C=C/C(=O)O | [5282709](https://pubchem.ncbi.nlm.nih.gov/compound/5282709) |
| 527 | [3-Methyl-1-nitrobutane](https://cb.imsc.res.in/imppat/phytochemical-detailedpage/IMPHY017685) | CC(CC[N+](=O)[O-])C | [69396](https://pubchem.ncbi.nlm.nih.gov/compound/69396) |
| 528 | [Gamma-undecalactone](https://cb.imsc.res.in/imppat/phytochemical-detailedpage/IMPHY017735) | CCCCCCCC1CCC(=O)O1 | [7714](https://pubchem.ncbi.nlm.nih.gov/compound/7714) |
| 529 | [4-Butyl-cyclohexen-3-one](https://cb.imsc.res.in/imppat/phytochemical-detailedpage/IMPHY017770) | CCCCC1=CCC(=O)CC1 | [81795502](https://pubchem.ncbi.nlm.nih.gov/compound/81795502) |
| 530 | [1,3-Dimethylnaphthalene](https://cb.imsc.res.in/imppat/phytochemical-detailedpage/IMPHY000004) | Cc1cc2ccccc2c(c1)C | [11327](https://pubchem.ncbi.nlm.nih.gov/compound/11327) |
| 531 | [Viscumiside A](https://cb.imsc.res.in/imppat/phytochemical-detailedpage/IMPHY002543) | OCC1OC(Oc2cc3OC(CC(=O)c3c(c2)O)c2ccc(c(c2)OC)O)C(C(C1O)O)O | [14035424](https://pubchem.ncbi.nlm.nih.gov/compound/14035424) |
| 532 | [Flavoyadorinin B](https://cb.imsc.res.in/imppat/phytochemical-detailedpage/IMPHY002869) | OC[C@H]1O[C@@H](Oc2ccc(cc2OC)c2cc(=O)c3c(o2)cc(cc3O)OC)[C@@H]([C@H]([C@@H]1O)O)O | [14376376](https://pubchem.ncbi.nlm.nih.gov/compound/14376376) |
| 533 | [Homoflavoyadorinin-B](https://cb.imsc.res.in/imppat/phytochemical-detailedpage/IMPHY002892) | OC[C@H]1O[C@@H](Oc2ccc(cc2OC)c2cc(=O)c3c(o2)cc(cc3O)OC)[C@@H]([C@H]([C@@H]1O)O)O[C@@H]1OC[C@]([C@H]1O)(O)CO | [14376380](https://pubchem.ncbi.nlm.nih.gov/compound/14376380) |
| 534 | [Choline](https://cb.imsc.res.in/imppat/phytochemical-detailedpage/IMPHY004055) | OCC[N+](C)(C)C | [305](https://pubchem.ncbi.nlm.nih.gov/compound/305) |
| 535 | [Liriodendrin](https://cb.imsc.res.in/imppat/phytochemical-detailedpage/IMPHY004102) | OC[C@H]1O[C@@H](Oc2c(OC)cc(cc2OC)[C@H]2OC[C@H]3[C@@H]2CO[C@@H]3c2cc(OC)c(c(c2)OC)O[C@@H]2O[C@H](CO)[C@H]([C@@H]([C@H]2O)O)O)[C@@H]([C@H]([C@@H]1O)O)O | [21603207](https://pubchem.ncbi.nlm.nih.gov/compound/21603207) |
| 536 | [Betulin](https://cb.imsc.res.in/imppat/phytochemical-detailedpage/IMPHY004271) | OC[C@@]12CC[C@H]([C@@H]2[C@@H]2[C@](CC1)(C)[C@]1(C)CC[C@@H]3[C@]([C@H]1CC2)(C)CC[C@@H](C3(C)C)O)C(=C)C | [72326](https://pubchem.ncbi.nlm.nih.gov/compound/72326) |
| 537 | [Rhamnazin](https://cb.imsc.res.in/imppat/phytochemical-detailedpage/IMPHY004329) | COc1cc(O)c2c(c1)oc(c(c2=O)O)c1ccc(c(c1)OC)O | [5320945](https://pubchem.ncbi.nlm.nih.gov/compound/5320945) |
| 538 | [Kumatakenin](https://cb.imsc.res.in/imppat/phytochemical-detailedpage/IMPHY004360) | COc1c(oc2c(c1=O)c(O)cc(c2)OC)c1ccc(cc1)O | [5318869](https://pubchem.ncbi.nlm.nih.gov/compound/5318869) |
| 539 | [Coniferin](https://cb.imsc.res.in/imppat/phytochemical-detailedpage/IMPHY004603) | OC/C=C/c1ccc(c(c1)OC)O[C@@H]1O[C@H](CO)[C@H]([C@@H]([C@H]1O)O)O | [5280372](https://pubchem.ncbi.nlm.nih.gov/compound/5280372) |
| 540 | [Kynurenine](https://cb.imsc.res.in/imppat/phytochemical-detailedpage/IMPHY007085) | OC(=O)C(CC(=O)c1ccccc1N)N | [846](https://pubchem.ncbi.nlm.nih.gov/compound/846) |
| 541 | [Isorhamnetin](https://cb.imsc.res.in/imppat/phytochemical-detailedpage/IMPHY008724) | COc1cc(ccc1O)c1oc2cc(O)cc(c2c(=O)c1O)O | [5281654](https://pubchem.ncbi.nlm.nih.gov/compound/5281654) |
| 542 | [Avicularin](https://cb.imsc.res.in/imppat/phytochemical-detailedpage/IMPHY011725) | OC[C@@H]1O[C@H]([C@@H]([C@H]1O)O)Oc1c(oc2c(c1=O)c(O)cc(c2)O)c1ccc(c(c1)O)O | [5490064](https://pubchem.ncbi.nlm.nih.gov/compound/5490064) |
| 543 | [Acetylcholine](https://cb.imsc.res.in/imppat/phytochemical-detailedpage/IMPHY012020) | CC(=O)OCC[N+](C)(C)C | [187](https://pubchem.ncbi.nlm.nih.gov/compound/187) |
| 544 | [Sakuranetin](https://cb.imsc.res.in/imppat/phytochemical-detailedpage/IMPHY012232) | COc1cc2O[C@@H](CC(=O)c2c(c1)O)c1ccc(cc1)O | [73571](https://pubchem.ncbi.nlm.nih.gov/compound/73571) |
| 545 | [Propionylcholine](https://cb.imsc.res.in/imppat/phytochemical-detailedpage/IMPHY015908) | CCC(=O)OCC[N+](C)(C)C | [75612](https://pubchem.ncbi.nlm.nih.gov/compound/75612) |
| 546 | [Scopolin](https://cb.imsc.res.in/imppat/phytochemical-detailedpage/IMPHY003411) | OC[C@H]1O[C@@H](Oc2cc3oc(=O)ccc3cc2OC)[C@@H]([C@H]([C@@H]1O)O)O | [439514](https://pubchem.ncbi.nlm.nih.gov/compound/439514) |
| 547 | [Oleandrin](https://cb.imsc.res.in/imppat/phytochemical-detailedpage/IMPHY003634) | CO[C@H]1C[C@H](O[C@H]2CC[C@]3([C@@H](C2)CC[C@@H]2[C@@H]3CC[C@]3([C@]2(O)C[C@@H]([C@@H]3C2=CC(=O)OC2)OC(=O)C)C)C)O[C@H]([C@@H]1O)C | [11541511](https://pubchem.ncbi.nlm.nih.gov/compound/11541511) |
| 548 | [Barban](https://cb.imsc.res.in/imppat/phytochemical-detailedpage/IMPHY006170) | ClCC#CCOC(=O)Nc1cccc(c1)Cl | [7551](https://pubchem.ncbi.nlm.nih.gov/compound/7551) |
| 549 | [Pregnenolone](https://cb.imsc.res.in/imppat/phytochemical-detailedpage/IMPHY007300) | O[C@H]1CC[C@]2(C(=CC[C@@H]3[C@@H]2CC[C@]2([C@H]3CC[C@@H]2C(=O)C)C)C1)C | [8955](https://pubchem.ncbi.nlm.nih.gov/compound/8955) |
| 550 | [Odoroside A](https://cb.imsc.res.in/imppat/phytochemical-detailedpage/IMPHY011715) | CO[C@@H]1C[C@H](O[C@H]2CC[C@]3([C@@H](C2)CC[C@@H]2[C@@H]3CC[C@]3([C@]2(O)CC[C@@H]3C2=CC(=O)OC2)C)C)O[C@@H]([C@@H]1O)C | [44425145](https://pubchem.ncbi.nlm.nih.gov/compound/44425145) |
| 551 | [Oleagenin](https://cb.imsc.res.in/imppat/phytochemical-detailedpage/IMPHY000269) | O[C@H]1CC[C@]2([C@@H](C1)CC[C@@]13[C@@H]2CC[C@](C3=O)(C)[C@H](CC1)C1=CC(=O)OC1)C | [101967000](https://pubchem.ncbi.nlm.nih.gov/compound/101967000) |
| 552 | [Adynerin](https://cb.imsc.res.in/imppat/phytochemical-detailedpage/IMPHY000953) | CO[C@@H]1C[C@H](O[C@H]2CC[C@]3([C@@H](C2)CC[C@@]24[C@@H]3CC[C@]3([C@]4(O2)CC[C@@H]3C2=CC(=O)OC2)C)C)O[C@@H]([C@@H]1O)C | [441840](https://pubchem.ncbi.nlm.nih.gov/compound/441840) |
| 553 | [16-Dehydroadynerigenin-beta-d-diginoside](https://cb.imsc.res.in/imppat/phytochemical-detailedpage/IMPHY002330) | CO[C@@H]1[C@@H](O)[C@H](O[C@H]2CC[C@]3([C@@H](C2)CC[C@@]24[C@@H]3CC[C@]3([C@]4(O2)CC=C3C2=CC(=O)OC2)C)C)O[C@@H]([C@@H]1O)C | No id |
| 554 | [Neriaside](https://cb.imsc.res.in/imppat/phytochemical-detailedpage/IMPHY002707) | CO[C@@H]1C[C@H](O[C@H]2CC[C@]3([C@@H](C2)CCC(=O)[C@@H]3CC[C@@]2(C)[C@@H](O)CC[C@@H]2C2=CC(=O)OC2)C)O[C@@H]([C@@H]1O)C | [101324843](https://pubchem.ncbi.nlm.nih.gov/compound/101324843) |
| 555 | [Digitoxigenin](https://cb.imsc.res.in/imppat/phytochemical-detailedpage/IMPHY003770) | O[C@H]1CC[C@]2([C@@H](C1)CC[C@@H]1[C@@H]2CC[C@]2([C@]1(O)CC[C@@H]2C1=CC(=O)OC1)C)C | [4369270](https://pubchem.ncbi.nlm.nih.gov/compound/4369270) |
| 556 | [Odoroside K](https://cb.imsc.res.in/imppat/phytochemical-detailedpage/IMPHY004044) | CO[C@@H]1[C@@H](O)[C@H](O[C@H]2CC[C@]3([C@H](C2)CC[C@@H]2[C@@H]3CC[C@]3([C@]2(O)CC[C@@H]3C2=CC(=O)OC2)C)C)O[C@@H]([C@@H]1O[C@@H]1O[C@H](CO)[C@H]([C@@H]([C@H]1O)O)O)C | [101632315](https://pubchem.ncbi.nlm.nih.gov/compound/101632315) |
| 557 | [L-(+)-Arabinose](https://cb.imsc.res.in/imppat/phytochemical-detailedpage/IMPHY004187) | OC[C@@H]([C@@H]([C@H](C=O)O)O)O | [5460291](https://pubchem.ncbi.nlm.nih.gov/compound/5460291) |
| 558 | [Dambonitol](https://cb.imsc.res.in/imppat/phytochemical-detailedpage/IMPHY005174) | CO[C@@H]1C(O)[C@H](OC)[C@H](C([C@H]1O)O)O | [21627888](https://pubchem.ncbi.nlm.nih.gov/compound/21627888) |
| 559 | [Oleandrigenin](https://cb.imsc.res.in/imppat/phytochemical-detailedpage/IMPHY007502) | O[C@H]1CC[C@]2([C@@H](C1)CC[C@@H]1[C@@H]2CC[C@]2([C@]1(O)C[C@@H]([C@@H]2C1=CC(=O)OC1)OC(=O)C)C)C | [9802865](https://pubchem.ncbi.nlm.nih.gov/compound/9802865) |
| 560 | [Kaneric acid](https://cb.imsc.res.in/imppat/phytochemical-detailedpage/IMPHY008362) | C[C@@H]1CC[C@]2([C@H]([C@H]1C)C1=CC[C@H]3[C@@]([C@@]1(CC2)C)(C)CCC1[C@]3(C)[C@H](O)C[C@@H](C1(C)C)O)C(=O)O | [183863](https://pubchem.ncbi.nlm.nih.gov/compound/183863) |
| 561 | [Neriantin](https://cb.imsc.res.in/imppat/phytochemical-detailedpage/IMPHY008577) | OC[C@H]1OC(O[C@H]2CC[C@]3([C@@H](C2)CC[C@@H]2[C@@H]3CC[C@]3(C2C(O)C=C3C2=CC(=O)OC2)C)C)[C@@H]([C@H]([C@@H]1O)O)O | [12313293](https://pubchem.ncbi.nlm.nih.gov/compound/12313293) |
| 562 | [Oleanderol](https://cb.imsc.res.in/imppat/phytochemical-detailedpage/IMPHY009450) | OC[C@@]12CC[C@@]3([C@@H](C1=CC[C@H]1[C@@]2(C)CC[C@@H]2[C@]1(C)CC[C@@H](C2(C)C)O)[C@@H](CC3)C(=C)C)CO | [189260](https://pubchem.ncbi.nlm.nih.gov/compound/189260) |
| 563 | [Urs-12(13)-ene](https://cb.imsc.res.in/imppat/phytochemical-detailedpage/IMPHY011000) | C[C@@H]1CC[C@]2([C@@H]([C@H]1C)C1=CC[C@H]3[C@@]([C@@]1(CC2)C)(C)CC[C@@H]1[C@]3(C)CCCC1(C)C)C | [12302949](https://pubchem.ncbi.nlm.nih.gov/compound/12302949) |
| 564 | [Desacetyloleandrin](https://cb.imsc.res.in/imppat/phytochemical-detailedpage/IMPHY011368) | CO[C@H]1C[C@H](O[C@H]2CC[C@]3([C@@H](C2)CC[C@@H]2[C@@H]3CC[C@]3([C@]2(O)C[C@@H]([C@@H]3C2=CC(=O)OC2)O)C)C)O[C@H]([C@@H]1O)C | [76962086](https://pubchem.ncbi.nlm.nih.gov/compound/76962086) |
| 565 | [Urechitoxin](https://cb.imsc.res.in/imppat/phytochemical-detailedpage/IMPHY011417) | CO[C@H]1C[C@H](O[C@H]2CC[C@]3([C@@H](C2)CC[C@@H]2[C@@H]3CC[C@]3([C@]2(O)C[C@@H]([C@@H]3C2=CC(=O)OC2)OC(=O)C)C)C)O[C@H]([C@@H]1O[C@@H]1O[C@H](CO)[C@H]([C@@H]([C@H]1O)O)O)C | [171542](https://pubchem.ncbi.nlm.nih.gov/compound/171542) |
| 566 | [Isoneriucoumaric acid](https://cb.imsc.res.in/imppat/phytochemical-detailedpage/IMPHY011535) | O=C(O[C@@H]1C[C@@]2(C)[C@H](C([C@H]1O)(C)C)CC[C@@]1([C@@H]2CC=C2[C@@]1(C)CC[C@@]1([C@H]2[C@@H](C)[C@H](C)CC1)C(=O)O)C)/C=C/c1ccc(cc1)O | [10100394](https://pubchem.ncbi.nlm.nih.gov/compound/10100394) |
| 567 | [Bicuculline](https://cb.imsc.res.in/imppat/phytochemical-detailedpage/IMPHY011634) | CN1CCc2c([C@H]1[C@@H]1OC(=O)c3c1ccc1c3OCO1)cc1c(c2)OCO1 | [10237](https://pubchem.ncbi.nlm.nih.gov/compound/10237) |
| 568 | [Oxotremorine](https://cb.imsc.res.in/imppat/phytochemical-detailedpage/IMPHY012044) | O=C1CCCN1CC#CCN1CCCC1 | [4630](https://pubchem.ncbi.nlm.nih.gov/compound/4630) |
| 569 | [D-Galactose](https://cb.imsc.res.in/imppat/phytochemical-detailedpage/IMPHY012050) | OC[C@H]1OC(O)[C@@H]([C@H]([C@H]1O)O)O | [6036](https://pubchem.ncbi.nlm.nih.gov/compound/6036) |
| 570 | [Methyl 8,16-dihydroxyhexadecanoate](https://cb.imsc.res.in/imppat/phytochemical-detailedpage/IMPHY013489) | OCCCCCCCCC(CCCCCCC(=O)OC)O | [54396466](https://pubchem.ncbi.nlm.nih.gov/compound/54396466) |
| 571 | [Adynerigenin beta-neritrioside](https://cb.imsc.res.in/imppat/phytochemical-detailedpage/IMPHY013587) | CO[C@@H]1C[C@H](O[C@H]2CC[C@]3([C@@H](C2)CC[C@@]24[C@@H]3CC[C@]3([C@]4(O2)CC[C@@H]3C2=CC(=O)OC2)C)C)O[C@@H]([C@@H]1O[C@@H]1O[C@H](CO[C@@H]2O[C@H](CO)[C@H]([C@@H]([C@H]2O)O)O)[C@H]([C@@H]([C@H]1O)O)O)C | [91886678](https://pubchem.ncbi.nlm.nih.gov/compound/91886678) |
| 572 | [C15H18O7.C15H16O6](https://cb.imsc.res.in/imppat/phytochemical-detailedpage/IMPHY014048) | CC(=C)[C@@H]1[C@@H]2OC(=O)[C@H]1[C@]1([C@]3([C@@H]2OC(=O)[C@]23[C@@H](C1)O2)C)O.O=C1O[C@H]2[C@H]([C@@H]1[C@]1(O)C[C@@H]3[C@]4([C@@]1([C@@H]2OC4=O)C)O3)C(O)(C)C | [5311359](https://pubchem.ncbi.nlm.nih.gov/compound/5311359) |
| 573 | [Adynerigenine](https://cb.imsc.res.in/imppat/phytochemical-detailedpage/IMPHY014335) | O[C@H]1CC[C@]2([C@@H](C1)CC[C@@]13[C@@H]2CC[C@]2([C@]3(O1)CC[C@@H]2C1=CC(=O)OC1)C)C | [15558417](https://pubchem.ncbi.nlm.nih.gov/compound/15558417) |
| 574 | [Adynerigenin beta-odorotrioside](https://cb.imsc.res.in/imppat/phytochemical-detailedpage/IMPHY014578) | CO[C@@H]1[C@@H](O)[C@H](O[C@H]2CC[C@]3([C@@H](C2)CC[C@@]24[C@@H]3CC[C@]3([C@]4(O2)CC[C@@H]3C2=CC(=O)OC2)C)C)O[C@@H]([C@@H]1O[C@@H]1O[C@H](CO[C@@H]2O[C@H](CO)[C@H]([C@@H]([C@H]2O)O)O)[C@H]([C@@H]([C@H]1O)O)O)C | [101630857](https://pubchem.ncbi.nlm.nih.gov/compound/101630857) |
| 575 | [Neritaloside](https://cb.imsc.res.in/imppat/phytochemical-detailedpage/IMPHY014735) | CO[C@@H]1[C@@H](O)[C@H](O[C@H]2CC[C@]3([C@@H](C2)CC[C@@H]2[C@@H]3CC[C@]3([C@]2(O)C[C@@H]([C@@H]3C2=CC(=O)OC2)OC(=O)C)C)C)O[C@@H]([C@@H]1O)C | [44566654](https://pubchem.ncbi.nlm.nih.gov/compound/44566654) |
| 576 | [D-Galacturonic Acid](https://cb.imsc.res.in/imppat/phytochemical-detailedpage/IMPHY014919) | OC1O[C@H](C(=O)O)[C@@H]([C@@H]([C@H]1O)O)O | [439215](https://pubchem.ncbi.nlm.nih.gov/compound/439215) |
| 577 | [Solanoside](https://cb.imsc.res.in/imppat/phytochemical-detailedpage/IMPHY015028) | CO[C@H]1[C@@H](O)[C@H](O[C@H]2CC[C@]3([C@@H](C2)CC[C@@H]2[C@@H]3CC[C@]3([C@]2(O)CC[C@@H]3C2=CC(=O)OC2)C)C)O[C@H]([C@@H]1O)C | [165219](https://pubchem.ncbi.nlm.nih.gov/compound/165219) |
| 578 | [L-Rhamnose](https://cb.imsc.res.in/imppat/phytochemical-detailedpage/IMPHY015056) | O[C@H]1[C@H](C)OC([C@@H]([C@@H]1O)O)O | [25310](https://pubchem.ncbi.nlm.nih.gov/compound/25310) |
| 579 | [Neridienone A](https://cb.imsc.res.in/imppat/phytochemical-detailedpage/IMPHY000566) | O=C1CC[C@]2(C(=C1)C=C[C@@H]1[C@@H]2C[C@@H](O)[C@]2([C@H]1CC=C2C(=O)C)C)C | [100630](https://pubchem.ncbi.nlm.nih.gov/compound/100630) |
| 580 | [Neridienone B](https://cb.imsc.res.in/imppat/phytochemical-detailedpage/IMPHY000835) | OC[C@H]([C@H]1CC[C@@H]2[C@]1(C)C(=O)C[C@H]1[C@H]2C=CC2=CC(=O)CC[C@]12C)O | [44418781](https://pubchem.ncbi.nlm.nih.gov/compound/44418781) |
| 581 | [6-Dehydroprogesterone](https://cb.imsc.res.in/imppat/phytochemical-detailedpage/IMPHY001171) | O=C1CC[C@]2(C(=C1)C=C[C@@H]1[C@@H]2CC[C@]2([C@H]1CC[C@@H]2C(=O)C)C)C | [101994](https://pubchem.ncbi.nlm.nih.gov/compound/101994) |
| 582 | [Plumericin](https://cb.imsc.res.in/imppat/phytochemical-detailedpage/IMPHY003884) | COC(=O)C1=CO[C@H]2[C@H]3[C@@H]1C=C[C@@]13OC(=O)/C(=C/C)/[C@@H]1O2 | [5281545](https://pubchem.ncbi.nlm.nih.gov/compound/5281545) |
| 583 | [4-Hydroxyacetophenone](https://cb.imsc.res.in/imppat/phytochemical-detailedpage/IMPHY006148) | CC(=O)c1ccc(cc1)O | [7469](https://pubchem.ncbi.nlm.nih.gov/compound/7469) |
| 584 | [2',4'-Dihydroxyacetophenone](https://cb.imsc.res.in/imppat/phytochemical-detailedpage/IMPHY006552) | Oc1ccc(c(c1)O)C(=O)C | [6990](https://pubchem.ncbi.nlm.nih.gov/compound/6990) |
| 585 | [Odorosid B](https://cb.imsc.res.in/imppat/phytochemical-detailedpage/IMPHY011644) | CO[C@@H]1C[C@H](O[C@H]2CC[C@]3(C(C2)CC[C@@H]2[C@@H]3CC[C@]3([C@]2(O)CC[C@@H]3C2=CC(=O)OC2)C)C)O[C@@H]([C@@H]1O)C | [91809650](https://pubchem.ncbi.nlm.nih.gov/compound/91809650) |
| 586 | [Gitoxigenin](https://cb.imsc.res.in/imppat/phytochemical-detailedpage/IMPHY003245) | O[C@H]1CC[C@]2([C@@H](C1)CC[C@@H]1[C@@H]2CC[C@]2([C@]1(O)C[C@@H]([C@@H]2C1=CC(=O)OC1)O)C)C | [348482](https://pubchem.ncbi.nlm.nih.gov/compound/348482) |
| 587 | [3-[(3S,5R,8R,9S,10S,13R,14S,17S)-3-[(2R,4S,5S,6R)-5-[(2S,4S,5S,6R)-5-[(2S,4S,5S,6R)-4,5-dihydroxy-6-methyloxan-2-yl]oxy-4-hydroxy-6-methyloxan-2-yl]oxy-4-hydroxy-6-methyloxan-2-yl]oxy-14-hydroxy-10,13-dimethyl-1,2,3,4,5,6,7,8,9,11,12,15,16,17-tetradecahyd](https://cb.imsc.res.in/imppat/phytochemical-detailedpage/IMPHY003792) | O=C1OCC(=C1)[C@@H]1CC[C@]2([C@]1(C)CC[C@H]1[C@H]2CC[C@H]2[C@]1(C)CC[C@@H](C2)O[C@H]1C[C@H](O)[C@@H]([C@H](O1)C)O[C@H]1C[C@H](O)[C@@H]([C@H](O1)C)O[C@H]1C[C@H](O)[C@@H]([C@H](O1)C)O)O | [6281](https://pubchem.ncbi.nlm.nih.gov/compound/6281) |
| 588 | [Strospeside](https://cb.imsc.res.in/imppat/phytochemical-detailedpage/IMPHY004166) | CO[C@@H]1[C@@H](O)[C@H](O[C@H]2CC[C@]3([C@@H](C2)CC[C@@H]2[C@@H]3CC[C@]3([C@]2(O)C[C@@H]([C@@H]3C2=CC(=O)OC2)O)C)C)O[C@@H]([C@@H]1O)C | [21636336](https://pubchem.ncbi.nlm.nih.gov/compound/21636336) |
| 589 | [[(3S,5R,8R,9S,10S,13R,14S,16S,17R)-14-hydroxy-3-[(2R,4R,5S,6R)-5-hydroxy-4-methoxy-6-methyloxan-2-yl]oxy-10,13-dimethyl-17-(5-oxo-2H-furan-3-yl)-1,2,3,4,5,6,7,8,9,11,12,15,16,17-tetradecahydrocyclopenta[a]phenanthren-16-yl] 3-methylbutanoate](https://cb.imsc.res.in/imppat/phytochemical-detailedpage/IMPHY010069) | CO[C@@H]1C[C@H](O[C@H]2CC[C@]3([C@@H](C2)CC[C@@H]2[C@@H]3CC[C@]3([C@]2(O)C[C@@H]([C@@H]3C2=CC(=O)OC2)OC(=O)CC(C)C)C)C)O[C@@H]([C@@H]1O)C | [102093778](https://pubchem.ncbi.nlm.nih.gov/compound/102093778) |
| 590 | [Isophytol](https://cb.imsc.res.in/imppat/phytochemical-detailedpage/IMPHY000112) | C=CC(CCCC(CCCC(CCCC(C)C)C)C)(O)C | [10453](https://pubchem.ncbi.nlm.nih.gov/compound/10453) |
| 591 | [Neophytadiene](https://cb.imsc.res.in/imppat/phytochemical-detailedpage/IMPHY000121) | C=CC(=C)CCCC(CCCC(CCCC(C)C)C)C | [10446](https://pubchem.ncbi.nlm.nih.gov/compound/10446) |
| 592 | [Oleanderolide](https://cb.imsc.res.in/imppat/phytochemical-detailedpage/IMPHY000307) | O[C@H]1C[C@@H]2[C@@]3(C)CC[C@@H](C([C@@H]3CC[C@]2([C@]2([C@@]31OC(=O)[C@]1([C@H]3CC(C)(C)CC1)CC2)C)C)(C)C)O | [11113483](https://pubchem.ncbi.nlm.nih.gov/compound/11113483) |
| 593 | [Dotriacontane](https://cb.imsc.res.in/imppat/phytochemical-detailedpage/IMPHY000309) | CCCCCCCCCCCCCCCCCCCCCCCCCCCCCCCC | [11008](https://pubchem.ncbi.nlm.nih.gov/compound/11008) |
| 594 | [Pinene](https://cb.imsc.res.in/imppat/phytochemical-detailedpage/IMPHY000491) | CC1CCC2CC1C2(C)C | No id |
| 595 | [6,10,14-Trimethylpentadecan-2-one](https://cb.imsc.res.in/imppat/phytochemical-detailedpage/IMPHY001135) | CC(CCCC(C)C)CCCC(CCCC(=O)C)C | [10408](https://pubchem.ncbi.nlm.nih.gov/compound/10408) |
| 596 | [3beta,27-Dihydroxyurs-12-ene-28-oic acid](https://cb.imsc.res.in/imppat/phytochemical-detailedpage/IMPHY001284) | OC[C@@]12CC[C@@]3([C@H](C1=CC[C@H]1[C@@]2(C)CC[C@@H]2[C@]1(C)CC[C@@H](C2(C)C)O)[C@@H](C)[C@@H](CC3)C)C(=O)O | [44583858](https://pubchem.ncbi.nlm.nih.gov/compound/44583858) |
| 597 | [28-Norurs-12-ene-3beta-ol](https://cb.imsc.res.in/imppat/phytochemical-detailedpage/IMPHY001285) | C[C@@H]1CC[C@H]2[C@@H]([C@H]1C)C1=CC[C@H]3[C@@]([C@@]1(CC2)C)(C)CC[C@@H]1[C@]3(C)CC[C@@H](C1(C)C)O | [44583863](https://pubchem.ncbi.nlm.nih.gov/compound/44583863) |
| 598 | [2-[(1S,2R,4aR,6aR,6aS,6bR,8aR,10S,12aR,14bR)-10-hydroxy-1,2,6a,6b,9,9,12a-heptamethyl-2,3,4,5,6,6a,7,8,8a,10,11,12,13,14b-tetradecahydro-1H-picen-4a-yl]acetaldehyde](https://cb.imsc.res.in/imppat/phytochemical-detailedpage/IMPHY001286) | O=CC[C@@]12CC[C@H]([C@@H]([C@H]2C2=CC[C@H]3[C@@]([C@@]2(CC1)C)(C)CC[C@@H]1[C@]3(C)CC[C@@H](C1(C)C)O)C)C | [44583866](https://pubchem.ncbi.nlm.nih.gov/compound/44583866) |
| 599 | [3beta,13-Dihydroxyurs-11-en-28-oic acid](https://cb.imsc.res.in/imppat/phytochemical-detailedpage/IMPHY001292) | C[C@@H]1CC[C@]2([C@@H]([C@H]1C)[C@@]1(O)C=C[C@H]3[C@@]([C@@]1(CC2)C)(C)CC[C@@H]1[C@]3(C)CC[C@@H](C1(C)C)O)C(=O)O | [44583862](https://pubchem.ncbi.nlm.nih.gov/compound/44583862) |
| 600 | [(1R,2R,4aR,6aR,6aR,6bR,8aR,10S,12aR,14aR,14bR)-2,10-dihydroxy-1,2,6a,6b,9,9,12a-heptamethyl-1,3,4,5,6,6a,7,8,8a,10,11,12,13,14,14a,14b-hexadecahydropicene-4a-carboxylic acid](https://cb.imsc.res.in/imppat/phytochemical-detailedpage/IMPHY001391) | O[C@H]1CC[C@]2([C@H](C1(C)C)CC[C@@]1([C@@H]2CC[C@H]2[C@@]1(C)CC[C@@]1([C@H]2[C@@H](C)[C@](C)(O)CC1)C(=O)O)C)C | [44583857](https://pubchem.ncbi.nlm.nih.gov/compound/44583857) |
| 601 | [Methyl heptadecanoate](https://cb.imsc.res.in/imppat/phytochemical-detailedpage/IMPHY001494) | CCCCCCCCCCCCCCCCC(=O)OC | [15609](https://pubchem.ncbi.nlm.nih.gov/compound/15609) |
| 602 | [(20S,28S)-28-Methoxy-20,28-epoxytaraxasterane-3beta-ol](https://cb.imsc.res.in/imppat/phytochemical-detailedpage/IMPHY001735) | CO[C@H]1O[C@@]2(C)CC[C@]31CC[C@@]1([C@@H]([C@H]3[C@@H]2C)CC[C@H]2[C@@]1(C)CC[C@@H]1[C@]2(C)CC[C@@H](C1(C)C)O)C | [16083124](https://pubchem.ncbi.nlm.nih.gov/compound/16083124) |
| 603 | [(20S)-20,28-Epoxytaraxastera-21-ene-3beta-ol](https://cb.imsc.res.in/imppat/phytochemical-detailedpage/IMPHY001737) | O[C@H]1CC[C@]2([C@H](C1(C)C)CC[C@@]1([C@@H]2CC[C@H]2[C@@]1(C)CC[C@@]13[C@@H]2[C@H](C)[C@@](C)(OC1)C=C3)C)C | [16083125](https://pubchem.ncbi.nlm.nih.gov/compound/16083125) |
| 604 | [Phenyl glucoside](https://cb.imsc.res.in/imppat/phytochemical-detailedpage/IMPHY001894) | OC[C@H]1OC(Oc2ccccc2)[C@@H]([C@H]([C@@H]1O)O)O | [11701599](https://pubchem.ncbi.nlm.nih.gov/compound/11701599) |
| 605 | [Vanillin](https://cb.imsc.res.in/imppat/phytochemical-detailedpage/IMPHY001931) | COc1cc(C=O)ccc1O | [1183](https://pubchem.ncbi.nlm.nih.gov/compound/1183) |
| 606 | [(3beta)-3,27-Dihydroxyolean-12-en-28-oic acid](https://cb.imsc.res.in/imppat/phytochemical-detailedpage/IMPHY002025) | OC[C@@]12CC[C@@]3([C@H](C1=CC[C@H]1[C@@]2(C)CC[C@@H]2[C@]1(C)CC[C@@H](C2(C)C)O)CC(CC3)(C)C)C(=O)O | [12001894](https://pubchem.ncbi.nlm.nih.gov/compound/12001894) |
| 607 | [(1R,6S,10R,11R,14S,16R,19R,20S,21S,24S)-14,24-dihydroxy-4,4,11,15,15,19,20-heptamethyl-22-oxahexacyclo[19.2.1.01,6.07,20.010,19.011,16]tetracos-7-en-23-one](https://cb.imsc.res.in/imppat/phytochemical-detailedpage/IMPHY002317) | O[C@H]1CC[C@]2([C@H](C1(C)C)CC[C@@]1([C@@H]2CC=C2[C@@]1(C)[C@@H]1OC(=O)[C@]3([C@H]2CC(C)(C)CC3)[C@@H]1O)C)C | [101281384](https://pubchem.ncbi.nlm.nih.gov/compound/101281384) |
| 608 | [3',4'-Dimethoxyacetophenone](https://cb.imsc.res.in/imppat/phytochemical-detailedpage/IMPHY002810) | COc1cc(ccc1OC)C(=O)C | [14328](https://pubchem.ncbi.nlm.nih.gov/compound/14328) |
| 609 | [Methyl arachidate](https://cb.imsc.res.in/imppat/phytochemical-detailedpage/IMPHY002876) | CCCCCCCCCCCCCCCCCCCC(=O)OC | [14259](https://pubchem.ncbi.nlm.nih.gov/compound/14259) |
| 610 | [(2S,3R,4S,5S,6R)-2-[(2R,3R,4R,5R,6R)-6-[(6R,7S,9S,10S,13S,15R,16R)-10,15-dihydroxy-7,9,13-trimethylspiro[5-oxapentacyclo[10.8.0.02,9.04,8.013,18]icosane-6,2'-oxane]-16-yl]oxy-4,5-dihydroxy-2-(hydroxymethyl)oxan-3-yl]oxy-6-(hydroxymethyl)oxane-3,4,5-triol](https://cb.imsc.res.in/imppat/phytochemical-detailedpage/IMPHY003071) | OC[C@H]1O[C@@H](O[C@@H]2CC3CCC4C([C@]3(C[C@H]2O)C)C[C@@H]([C@]2(C4CC3C2[C@@H]([C@]2(O3)CCCCO2)C)C)O)[C@@H]([C@H]([C@H]1O[C@@H]1O[C@H](CO)[C@H]([C@@H]([C@H]1O)O)O)O)O | [3084119](https://pubchem.ncbi.nlm.nih.gov/compound/3084119) |
| 611 | [2-Methoxy-4-vinylphenol](https://cb.imsc.res.in/imppat/phytochemical-detailedpage/IMPHY003495) | COc1cc(C=C)ccc1O | [332](https://pubchem.ncbi.nlm.nih.gov/compound/332) |
| 612 | [2,4-Decadienal](https://cb.imsc.res.in/imppat/phytochemical-detailedpage/IMPHY003723) | CCCCC/C=C/C=C/C=O | [5283349](https://pubchem.ncbi.nlm.nih.gov/compound/5283349) |
| 613 | [Uzarigenin](https://cb.imsc.res.in/imppat/phytochemical-detailedpage/IMPHY003772) | O[C@H]1CC[C@]2([C@H](C1)CC[C@@H]1[C@@H]2CC[C@]2([C@]1(O)CC[C@@H]2C1=CC(=O)OC1)C)C | [92760](https://pubchem.ncbi.nlm.nih.gov/compound/92760) |
| 614 | [Longifolene](https://cb.imsc.res.in/imppat/phytochemical-detailedpage/IMPHY004286) | C=C1C2CCC3C1(C)CCCC(C23)(C)C | [289151](https://pubchem.ncbi.nlm.nih.gov/compound/289151) |
| 615 | [Herbacetin](https://cb.imsc.res.in/imppat/phytochemical-detailedpage/IMPHY004393) | Oc1ccc(cc1)c1oc2c(O)c(O)cc(c2c(=O)c1O)O | [5280544](https://pubchem.ncbi.nlm.nih.gov/compound/5280544) |
| 616 | [28-Norurs-12-ene-3beta,17beta-diol](https://cb.imsc.res.in/imppat/phytochemical-detailedpage/IMPHY005732) | C[C@@H]1CC[C@]2([C@@H]([C@H]1C)C1=CC[C@H]3[C@@]([C@@]1(CC2)C)(C)CC[C@@H]1[C@]3(C)CC[C@@H](C1(C)C)O)O | [21580512](https://pubchem.ncbi.nlm.nih.gov/compound/21580512) |
| 617 | [2-Ethylhexanol](https://cb.imsc.res.in/imppat/phytochemical-detailedpage/IMPHY006032) | CCCCC(CO)CC | [7720](https://pubchem.ncbi.nlm.nih.gov/compound/7720) |
| 618 | [Cardenolide](https://cb.imsc.res.in/imppat/phytochemical-detailedpage/IMPHY006386) | O=C1OCC(=C1)[C@H]1CC[C@H]2[C@]1(C)CC[C@H]1[C@H]2CCC2[C@]1(C)CCCC2 | [53957771](https://pubchem.ncbi.nlm.nih.gov/compound/53957771) |
| 619 | [Squalene](https://cb.imsc.res.in/imppat/phytochemical-detailedpage/IMPHY006486) | C/C(=CCC/C=C(/CC/C=C(/CCC=C(C)C)C)C)/CC/C=C(/CCC=C(C)C)C | [638072](https://pubchem.ncbi.nlm.nih.gov/compound/638072) |
| 620 | [Pregnane](https://cb.imsc.res.in/imppat/phytochemical-detailedpage/IMPHY007236) | CC[C@H]1CC[C@@H]2[C@]1(C)CC[C@H]1[C@H]2CCC2[C@]1(C)CCCC2 | [6857422](https://pubchem.ncbi.nlm.nih.gov/compound/6857422) |
| 621 | [Paradol](https://cb.imsc.res.in/imppat/phytochemical-detailedpage/IMPHY007369) | CCCCCCCC(=O)CCc1ccc(c(c1)OC)O | [94378](https://pubchem.ncbi.nlm.nih.gov/compound/94378) |
| 622 | [Ursonic acid](https://cb.imsc.res.in/imppat/phytochemical-detailedpage/IMPHY007550) | C[C@@H]1CC[C@]2([C@@H]([C@H]1C)C1=CC[C@H]3[C@@]([C@@]1(CC2)C)(C)CC[C@@H]1[C@]3(C)CCC(=O)C1(C)C)C(=O)O | [9890209](https://pubchem.ncbi.nlm.nih.gov/compound/9890209) |
| 623 | [Methyl pentadecanoate](https://cb.imsc.res.in/imppat/phytochemical-detailedpage/IMPHY008360) | CCCCCCCCCCCCCCC(=O)OC | [23518](https://pubchem.ncbi.nlm.nih.gov/compound/23518) |
| 624 | [Methyl heptanoate](https://cb.imsc.res.in/imppat/phytochemical-detailedpage/IMPHY008397) | CCCCCCC(=O)OC | [7826](https://pubchem.ncbi.nlm.nih.gov/compound/7826) |
| 625 | [Nonadecanoic acid](https://cb.imsc.res.in/imppat/phytochemical-detailedpage/IMPHY008595) | CCCCCCCCCCCCCCCCCCC(=O)O | [12591](https://pubchem.ncbi.nlm.nih.gov/compound/12591) |
| 626 | [Vitamin E](https://cb.imsc.res.in/imppat/phytochemical-detailedpage/IMPHY008937) | C[C@@H](CCC[C@]1(C)CCc2c(O1)c(C)c(c(c2C)O)C)CCC[C@@H](CCCC(C)C)C | [14985](https://pubchem.ncbi.nlm.nih.gov/compound/14985) |
| 627 | [Benzyl acetate](https://cb.imsc.res.in/imppat/phytochemical-detailedpage/IMPHY008991) | CC(=O)OCc1ccccc1 | [8785](https://pubchem.ncbi.nlm.nih.gov/compound/8785) |
| 628 | [Hexatriacontane](https://cb.imsc.res.in/imppat/phytochemical-detailedpage/IMPHY009484) | CCCCCCCCCCCCCCCCCCCCCCCCCCCCCCCCCCCC | [12412](https://pubchem.ncbi.nlm.nih.gov/compound/12412) |
| 629 | [2'-Hydroxyacetophenone](https://cb.imsc.res.in/imppat/phytochemical-detailedpage/IMPHY009784) | CC(=O)c1ccccc1O | [8375](https://pubchem.ncbi.nlm.nih.gov/compound/8375) |
| 630 | [Tetracontane](https://cb.imsc.res.in/imppat/phytochemical-detailedpage/IMPHY010882) | CCCCCCCCCCCCCCCCCCCCCCCCCCCCCCCCCCCCCCCC | [20149](https://pubchem.ncbi.nlm.nih.gov/compound/20149) |
| 631 | [luteolin-7-O-rutinoside](https://cb.imsc.res.in/imppat/phytochemical-detailedpage/IMPHY010929) | OC[C@@H]1O[C@H](OC[C@H]2OC(Oc3cc(O)c4c(c3)oc(cc4=O)c3ccc(c(c3)O)O)[C@@H]([C@H]([C@@H]2O)O)O)[C@H](C([C@@H]1O)O)O | [14032966](https://pubchem.ncbi.nlm.nih.gov/compound/14032966) |
| 632 | [beta-Tocopherol](https://cb.imsc.res.in/imppat/phytochemical-detailedpage/IMPHY011554) | C[C@H](CCC[C@@H](CCCC(C)C)C)CCC[C@]1(C)CCc2c(O1)c(C)cc(c2C)O | [6857447](https://pubchem.ncbi.nlm.nih.gov/compound/6857447) |
| 633 | [Gingerol](https://cb.imsc.res.in/imppat/phytochemical-detailedpage/IMPHY011608) | CCCCC[C@@H](CC(=O)CCc1ccc(c(c1)OC)O)O | [442793](https://pubchem.ncbi.nlm.nih.gov/compound/442793) |
| 634 | [alpha1-Sitosterol](https://cb.imsc.res.in/imppat/phytochemical-detailedpage/IMPHY011654) | C/C=C(C(C)C)/CC[C@H]([C@H]1CC[C@@H]2[C@]1(C)CC[C@H]1C2=CC[C@@H]2[C@]1(C)CC[C@@H]([C@H]2C)O)C | [9548595](https://pubchem.ncbi.nlm.nih.gov/compound/9548595) |
| 635 | [Odoroside G](https://cb.imsc.res.in/imppat/phytochemical-detailedpage/IMPHY011925) | CO[C@@H]1[C@@H](O)[C@H](O[C@H]2CC[C@]3([C@@H](C2)CC[C@@H]2[C@@H]3CC[C@]3([C@]2(O)CC[C@@H]3C2=CC(=O)OC2)C)C)O[C@@H]([C@@H]1O[C@@H]1O[C@H](CO[C@@H]2O[C@H](CO)[C@H]([C@@H]([C@H]2O)O)O)[C@H]([C@@H]([C@H]1O)O)O)C | [21636339](https://pubchem.ncbi.nlm.nih.gov/compound/21636339) |
| 636 | [Guaifenesin](https://cb.imsc.res.in/imppat/phytochemical-detailedpage/IMPHY012040) | OCC(COc1ccccc1OC)O | [3516](https://pubchem.ncbi.nlm.nih.gov/compound/3516) |
| 637 | [Methyl 12-methyltetradecanoate](https://cb.imsc.res.in/imppat/phytochemical-detailedpage/IMPHY012172) | CCC(CCCCCCCCCCC(=O)OC)C | [21206](https://pubchem.ncbi.nlm.nih.gov/compound/21206) |
| 638 | [Abietan-18-oic acid](https://cb.imsc.res.in/imppat/phytochemical-detailedpage/IMPHY012295) | CC(C1CCC2C(C1)CCC1C2(C)CCCC1(C)C(=O)O)C | [95347](https://pubchem.ncbi.nlm.nih.gov/compound/95347) |
| 639 | [Photocitral A](https://cb.imsc.res.in/imppat/phytochemical-detailedpage/IMPHY012307) | O=CC1C(C)CCC1C(=C)C | [102684](https://pubchem.ncbi.nlm.nih.gov/compound/102684) |
| 640 | [Odoroside F](https://cb.imsc.res.in/imppat/phytochemical-detailedpage/IMPHY012325) | COC1C(OC2OC(CO)C(C(C2O)O)O)C(OC2CCC3(C(C2)CCC2C3CCC3(C2(O)CCC3C2=CC(=O)OC2)C)C)OC(C1O)C | [120681](https://pubchem.ncbi.nlm.nih.gov/compound/120681) |
| 641 | [Oleaside A](https://cb.imsc.res.in/imppat/phytochemical-detailedpage/IMPHY012512) | COC1CC(OC2CCC3(C(C2)CCC24C3CCC(C4=O)(C)C(CC2)C2=CC(=O)OC2)C)OC(C1O)C | [434211](https://pubchem.ncbi.nlm.nih.gov/compound/434211) |
| 642 | [2-Propyl-tetrahydropyran-3-ol](https://cb.imsc.res.in/imppat/phytochemical-detailedpage/IMPHY012606) | CCCC1OCCCC1O | [541755](https://pubchem.ncbi.nlm.nih.gov/compound/541755) |
| 643 | [Methyl 13-octadecenoate](https://cb.imsc.res.in/imppat/phytochemical-detailedpage/IMPHY012608) | CCCCC=CCCCCCCCCCCCC(=O)OC | [543418](https://pubchem.ncbi.nlm.nih.gov/compound/543418) |
| 644 | [2-Cyclobutyl-2-propanol](https://cb.imsc.res.in/imppat/phytochemical-detailedpage/IMPHY012610) | CC(C1CCC1)(O)C | [547749](https://pubchem.ncbi.nlm.nih.gov/compound/547749) |
| 645 | [9-Eicosyne](https://cb.imsc.res.in/imppat/phytochemical-detailedpage/IMPHY012615) | CCCCCCCCCCC#CCCCCCCCC | [557019](https://pubchem.ncbi.nlm.nih.gov/compound/557019) |
| 646 | [9,17-Octadecadienal, (Z)-](https://cb.imsc.res.in/imppat/phytochemical-detailedpage/IMPHY012824) | C=CCCCCCC/C=CCCCCCCCC=O | [5365667](https://pubchem.ncbi.nlm.nih.gov/compound/5365667) |
| 647 | [5-[[6-O-(6-Deoxy-alpha-L-mannopyranosyl)-beta-D-glucopyranosyl]oxy]-2-(3,4-dihydroxyphenyl)-7-hydroxy-4H-1-benzopyran-4-one](https://cb.imsc.res.in/imppat/phytochemical-detailedpage/IMPHY013414) | OC1[C@@H](OC([C@H]([C@@H]1O)O)CO[C@@H]1OC(C)[C@@H](C([C@@H]1O)O)O)Oc1cc(O)cc2c1c(=O)cc(o2)c1ccc(c(c1)O)O | [44258131](https://pubchem.ncbi.nlm.nih.gov/compound/44258131) |
| 648 | [2,6-Di-O-methyl-d-galactopyranose](https://cb.imsc.res.in/imppat/phytochemical-detailedpage/IMPHY013568) | COC[C@@H]1OC(O)[C@H]([C@H]([C@H]1O)O)OC | [91699070](https://pubchem.ncbi.nlm.nih.gov/compound/91699070) |
| 649 | [6-Methyl-1,3,5-triazine-2,4-diamine](https://cb.imsc.res.in/imppat/phytochemical-detailedpage/IMPHY013768) | Cc1nc(N)nc(n1)N | [10949](https://pubchem.ncbi.nlm.nih.gov/compound/10949) |
| 650 | [7,10,13-Hexadecatrienoic acid methyl ester](https://cb.imsc.res.in/imppat/phytochemical-detailedpage/IMPHY013987) | CCC=CCC=CCC=CCCCCCC(=O)OC | [556196](https://pubchem.ncbi.nlm.nih.gov/compound/556196) |
| 651 | [1,2-Ethanediol, 1-(2-furanyl)-](https://cb.imsc.res.in/imppat/phytochemical-detailedpage/IMPHY013991) | OCC(c1ccco1)O | [566112](https://pubchem.ncbi.nlm.nih.gov/compound/566112) |
| 652 | [trans-2-Tridecen-1-ol](https://cb.imsc.res.in/imppat/phytochemical-detailedpage/IMPHY014087) | CCCCCCCCCC/C=C/CO | [5364949](https://pubchem.ncbi.nlm.nih.gov/compound/5364949) |
| 653 | [Dimethyl 4-O-methylhexopyranosiduronate](https://cb.imsc.res.in/imppat/phytochemical-detailedpage/IMPHY014305) | CO[C@H]1O[C@H](C(=O)OC)[C@H]([C@@H]([C@H]1O)O)OC | [14392981](https://pubchem.ncbi.nlm.nih.gov/compound/14392981) |
| 654 | [9,12,15-Octadecatrien-1-ol](https://cb.imsc.res.in/imppat/phytochemical-detailedpage/IMPHY014793) | OCCCCCCCC/C=C/C/C=C/C/C=C/CC | [5367327](https://pubchem.ncbi.nlm.nih.gov/compound/5367327) |
| 655 | [8,11-Octadecadienoic acid, methyl ester](https://cb.imsc.res.in/imppat/phytochemical-detailedpage/IMPHY015009) | CCCCCC/C=C/C/C=C/CCCCCCC(=O)OC | [5319737](https://pubchem.ncbi.nlm.nih.gov/compound/5319737) |
| 656 | [4-Tridecanone](https://cb.imsc.res.in/imppat/phytochemical-detailedpage/IMPHY017906) | CCCCCCCCCC(=O)CCC | [98673](https://pubchem.ncbi.nlm.nih.gov/compound/98673) |
| 657 | Okanin | O=C(/C=C/c1ccc(O)c(O)c1)c1ccc(O)c(O)c1O | [5281294](https://pubchem.ncbi.nlm.nih.gov/compound/5281294) |
| 658 | (1r,4as,5s)-5-[(1r,6s)-6-[(2e)-4-[(1s,5r,8ar)-5-(hydroxymethyl)-5,8a-dimethyl-2-methylidene-hexahydro-1h-naphthalen-1-yl]but-2-en-2-yl]-2-methylcyclohex-2-en-1-yl]-1,4a-dimethyl-6-methylidene-hexahydro-2h-naphthalene-1-carboxylic acid | C=C1CCC2[C@](C)(CCC[C@@]2(C)C(=O)O)[C@H]1[C@@H]1C(C)=CCC[C@@H]1/C(C)=C/C[C@H]1C(=C)CCC2[C@](C)(CO)CCC[C@@]21C | [101133771](https://pubchem.ncbi.nlm.nih.gov/compound/101133771) |
| 659 | Apigenin 7-o-β-glucoside | O=c1cc(-c2ccc(O)cc2)oc2cc(O[C@@H]3O[C@H](CO)[C@@H](O)[C@H](O)[C@H]3O)cc(O)c12 | [5280704](https://pubchem.ncbi.nlm.nih.gov/compound/5280704) |
| 660 | [Isoquercitrin](https://pubchem.ncbi.nlm.nih.gov/compound/51402807) | O=c1c(O[C@@H]2O[C@@H](CO)[C@H](O)[C@@H](O)[C@H]2O)c(-c2ccc(O)c(O)c2)oc2cc(O)cc(O)c12 | [51402807](https://pubchem.ncbi.nlm.nih.gov/compound/51402807) |
| 661 | Luteolin 7-o-glucoside | O=c1cc(-c2ccc(O)c(O)c2)oc2cc(O[C@@H]3O[C@H](CO)[C@@H](O)[C@H](O)[C@H]3O)cc(O)c12 | [5280637](https://pubchem.ncbi.nlm.nih.gov/compound/5280637) |
| 662 | Nictoflorin | C[C@@H]1O[C@@H](OC[C@H]2O[C@@H](Oc3c(-c4ccc(O)cc4)oc4cc(O)cc(O)c4c3=O)[C@H](O)[C@@H](O)[C@@H]2O)[C@H](O)[C@H](O)[C@H]1O | [5318767](https://pubchem.ncbi.nlm.nih.gov/compound/5318767) |
| 663 | Astragalin | O=c1c(O[C@@H]2O[C@H](CO)[C@@H](O)[C@H](O)[C@H]2O)c(-c2ccc(O)cc2)oc2cc(O)cc(O)c12 | [5282102](https://pubchem.ncbi.nlm.nih.gov/compound/5282102) |
| 664 | 3-rutinosyl quercetin | C[C@@H]1O[C@@H](OC[C@H]2O[C@@H](Oc3c(-c4ccc(O)c(O)c4)oc4cc(O)cc(O)c4c3=O)[C@H](O)[C@@H](O)[C@@H]2O)[C@H](O)[C@H](O)[C@H]1O | [5280805](https://pubchem.ncbi.nlm.nih.gov/compound/5280805) |
| 665 | (2e)-3-(3,4-dihydroxyphenyl)-1-(2-hydroxy-3-methoxy-4-{[(2s,3r,4s,5s,6r)-3,4,5-trihydroxy-6-(hydroxymethyl)oxan-2-yl]oxy}phenyl)prop-2-en-1-one | COc1c(O[C@@H]2O[C@H](CO)[C@@H](O)[C@H](O)[C@H]2O)ccc(C(=O)/C=C/c2ccc(O)c(O)c2)c1O | [5318928](https://pubchem.ncbi.nlm.nih.gov/compound/5318928) |
| 666 | Leptosin | COc1c(OC2OC(CO)C(O)C(O)C2O)ccc2c1OC(=Cc1ccc(O)c(O)c1)C2=O | [42607750](https://pubchem.ncbi.nlm.nih.gov/compound/42607750) |
| 667 | Leptosin | COc1c(O[C@@H]2O[C@H](CO)[C@@H](O)[C@H](O)[C@H]2O)ccc2c1O/C(=C\c1ccc(O)c(O)c1)C2=O | [6446647](https://pubchem.ncbi.nlm.nih.gov/compound/6446647) |
| 668 | 3-(3,4-dihydroxyphenyl)-1-(2-hydroxy-3-methoxy-4-{[3,4,5-trihydroxy-6-(hydroxymethyl)oxan-2-yl]oxy}phenyl)prop-2-en-1-one | COc1c(OC2OC(CO)C(O)C(O)C2O)ccc(C(=O)C=Cc2ccc(O)c(O)c2)c1O | [57002617](https://pubchem.ncbi.nlm.nih.gov/compound/57002617) |
| 669 | Berbamine | COc1cc2c3cc1Oc1c(OC)c(OC)cc4c1[C@@H](Cc1ccc(O)c(c1)Oc1ccc(cc1)C[C@@H]3N(C)CC2)N(C)CC4 | [275182](https://pubchem.ncbi.nlm.nih.gov/compound/275182) |
| 670 | Berbamunine | COc1cc2c(cc1O)[C@H](Cc1ccc(Oc3cc(C[C@@H]4c5cc(O)c(OC)cc5CCN4C)ccc3O)cc1)N(C)CC2 | [440585](https://pubchem.ncbi.nlm.nih.gov/compound/440585) |
| 671 | 3,4,11-trimethoxy-7,8-dihydro-6-azatetraphen-10-one | COC1=CC2=C3C=c4ccc(OC)c(OC)c4=CN3CCC2=CC1=O | [10065647](https://pubchem.ncbi.nlm.nih.gov/compound/10065647) |
| 672 | Pseudopalmatine | COc1cc2c(cc1OC)-c1cc3cc(OC)c(OC)cc3c[n+]1CC2 | [644002](https://pubchem.ncbi.nlm.nih.gov/compound/644002) |
| 673 | (1r,14s)-20,21,25-trimethoxy-15,30-dimethyl-7,23-dioxa-15,30-diazaheptacyclo[22.6.2.2³,⁶.1⁸,¹².1¹⁴,¹⁸.0²⁷,³¹.0²²,³³]hexatriaconta-3,5,8(34),9,11,18(33),19,21,24(32),25,27(31),35-dodecaen-9-ol | COc1cc2c3cc1Oc1c(OC)c(OC)cc4c1[C@H](Cc1ccc(O)c(c1)Oc1ccc(cc1)C[C@H]3N(C)CC2)N(C)CC4 | [12300053](https://pubchem.ncbi.nlm.nih.gov/compound/12300053) |
| 674 | 20,21,25-trimethoxy-15,30-dimethyl-7,23-dioxa-15,30-diazaheptacyclo[22.6.2.2³,⁶.1⁸,¹².1¹⁴,¹⁸.0²⁷,³¹.0²²,³³]hexatriaconta-3,5,8(34),9,11,18(33),19,21,24(32),25,27(31),35-dodecaen-9-ol | COc1cc2c3cc1Oc1c(OC)c(OC)cc4c1C(Cc1ccc(O)c(c1)Oc1ccc(cc1)CC3N(C)CC2)N(C)CC4 | [10170](https://pubchem.ncbi.nlm.nih.gov/compound/10170) |
| 675 | Berberine | COc1ccc2cc3[n+](cc2c1OC)CCc1cc2c(cc1-3)OCO2 | [2353](https://pubchem.ncbi.nlm.nih.gov/compound/2353) |
| 676 | 17-methoxy-5,7-dioxa-13-azapentacyclo[11.8.0.0²,¹⁰.0⁴,⁸.0¹⁵,²⁰]henicosa-1(21),2,4(8),9,14,17,19-heptaen-16-one | COC1=CC=C2C=C3c4cc5c(cc4CCN3C=C2C1=O)OCO5 | [457914](https://pubchem.ncbi.nlm.nih.gov/compound/457914) |
| 677 | Oxyacanthine | COc1cc2c3cc1Oc1c(OC)c(OC)cc4c1[C@H](Cc1ccc(cc1)Oc1cc(ccc1O)C[C@H]3N(C)CC2)N(C)CC4 | [442333](https://pubchem.ncbi.nlm.nih.gov/compound/442333) |
| 678 | [Himachalol](https://cb.imsc.res.in/imppat/phytochemical-detailedpage/IMPHY002103) | CC1=C[C@H]2[C@@H](CC1)[C@](C)(O)CCCC2(C)C | [121536](https://pubchem.ncbi.nlm.nih.gov/compound/121536) |
| 679 | [Keracyanin](https://cb.imsc.res.in/imppat/phytochemical-detailedpage/IMPHY003138) | Oc1cc(O)c2c(c1)[o+]c(c(c2)O[C@@H]1O[C@H](CO[C@@H]2O[C@@H](C)[C@@H]([C@H]([C@H]2O)O)O)[C@H]([C@@H]([C@H]1O)O)O)c1ccc(c(c1)O)O.[Cl-] | [29231](https://pubchem.ncbi.nlm.nih.gov/compound/29231) |
| 680 | [Isoamyl acetate](https://cb.imsc.res.in/imppat/phytochemical-detailedpage/IMPHY003519) | CC(CCOC(=O)C)C | [31276](https://pubchem.ncbi.nlm.nih.gov/compound/31276) |
| 681 | [(Z)-Non-2-enal](https://cb.imsc.res.in/imppat/phytochemical-detailedpage/IMPHY003759) | CCCCCC/C=CC=O | [5354833](https://pubchem.ncbi.nlm.nih.gov/compound/5354833) |
| 682 | [Cinnamyl acetate](https://cb.imsc.res.in/imppat/phytochemical-detailedpage/IMPHY004184) | CC(=O)OC/C=C/c1ccccc1 | [5282110](https://pubchem.ncbi.nlm.nih.gov/compound/5282110) |
| 683 | [Furaneol](https://cb.imsc.res.in/imppat/phytochemical-detailedpage/IMPHY005803) | CC1OC(=C(C1=O)O)C | [19309](https://pubchem.ncbi.nlm.nih.gov/compound/19309) |
| 684 | [Phenethyl acetate](https://cb.imsc.res.in/imppat/phytochemical-detailedpage/IMPHY006003) | CC(=O)OCCc1ccccc1 | [7654](https://pubchem.ncbi.nlm.nih.gov/compound/7654) |
| 685 | [Phenylacetic acid](https://cb.imsc.res.in/imppat/phytochemical-detailedpage/IMPHY007598) | OC(=O)Cc1ccccc1 | [999](https://pubchem.ncbi.nlm.nih.gov/compound/999) |
| 686 | [3-Phenylpropionic acid](https://cb.imsc.res.in/imppat/phytochemical-detailedpage/IMPHY008722) | OC(=O)CCc1ccccc1 | [107](https://pubchem.ncbi.nlm.nih.gov/compound/107) |
| 687 | [delta-Decalactone](https://cb.imsc.res.in/imppat/phytochemical-detailedpage/IMPHY009939) | CCCCCC1CCCC(=O)O1 | [12810](https://pubchem.ncbi.nlm.nih.gov/compound/12810) |
| 688 | [beta-Cadinene](https://cb.imsc.res.in/imppat/phytochemical-detailedpage/IMPHY010603) | CC1=CC[C@@H]2[C@@H](C1)[C@@H](CC=C2C)C(C)C | [10657](https://pubchem.ncbi.nlm.nih.gov/compound/10657) |
| 689 | [(-)-alpha-Himachalene](https://cb.imsc.res.in/imppat/phytochemical-detailedpage/IMPHY010609) | CC1=C[C@H]2[C@@H](CC1)C(=C)CCCC2(C)C | [11830551](https://pubchem.ncbi.nlm.nih.gov/compound/11830551) |
| 690 | [alpha-Gurjunene](https://cb.imsc.res.in/imppat/phytochemical-detailedpage/IMPHY011667) | C[C@@H]1CC[C@@H]2[C@H](C3=C(CC[C@H]13)C)C2(C)C | [15560276](https://pubchem.ncbi.nlm.nih.gov/compound/15560276) |
| 691 | [Neral](https://cb.imsc.res.in/imppat/phytochemical-detailedpage/IMPHY011790) | O=C/C=C(CCC=C(C)C)/C | [643779](https://pubchem.ncbi.nlm.nih.gov/compound/643779) |
| 692 | [Cinnamaldehyde](https://cb.imsc.res.in/imppat/phytochemical-detailedpage/IMPHY011882) | O=C/C=C/c1ccccc1 | [637511](https://pubchem.ncbi.nlm.nih.gov/compound/637511) |
| 693 | [(2S,4R)-4-methyl-2-(2-methylprop-1-en-1-yl)tetrahydro-2H-pyran](https://cb.imsc.res.in/imppat/phytochemical-detailedpage/IMPHY012179) | C[C@@H]1CCO[C@@H](C1)C=C(C)C | [1712087](https://pubchem.ncbi.nlm.nih.gov/compound/1712087) |
| 694 | [Cyanidin 3-glucoside](https://cb.imsc.res.in/imppat/phytochemical-detailedpage/IMPHY014890) | OCC1OC(Oc2cc3c(O)cc(cc3[o+]c2c2ccc(c(c2)O)O)O)C(C(C1O)O)O.[Cl-] | [12303220](https://pubchem.ncbi.nlm.nih.gov/compound/12303220) |
| 695 | [2-Heptenal](https://cb.imsc.res.in/imppat/phytochemical-detailedpage/IMPHY003649) | CCCC/C=C/C=O | [5283316](https://pubchem.ncbi.nlm.nih.gov/compound/5283316) |
| 696 | [Zingiberene](https://cb.imsc.res.in/imppat/phytochemical-detailedpage/IMPHY011745) | CC(=CCC[C@@H]([C@H]1CC=C(C=C1)C)C)C | [92776](https://pubchem.ncbi.nlm.nih.gov/compound/92776) |
| 697 | [Valencene](https://cb.imsc.res.in/imppat/phytochemical-detailedpage/IMPHY011896) | CC(=C)[C@@H]1CCC2=CCC[C@H]([C@@]2(C1)C)C | [9855795](https://pubchem.ncbi.nlm.nih.gov/compound/9855795) |
| 698 | [Citric acid](https://cb.imsc.res.in/imppat/phytochemical-detailedpage/IMPHY003500) | OC(=O)C(CC(=O)O)(CC(=O)O)O | [311](https://pubchem.ncbi.nlm.nih.gov/compound/311) |
| 699 | [Levulinic acid](https://cb.imsc.res.in/imppat/phytochemical-detailedpage/IMPHY001858) | CC(=O)CCC(=O)O | [11579](https://pubchem.ncbi.nlm.nih.gov/compound/11579) |
| 700 | [Pelargonidin](https://cb.imsc.res.in/imppat/phytochemical-detailedpage/IMPHY003437) | Oc1ccc(cc1)c1[o+]c2cc(O)cc(c2cc1O)O | [440832](https://pubchem.ncbi.nlm.nih.gov/compound/440832) |
| 701 | [Eriodictyol](https://cb.imsc.res.in/imppat/phytochemical-detailedpage/IMPHY004038) | Oc1cc2O[C@@H](CC(=O)c2c(c1)O)c1ccc(c(c1)O)O | [440735](https://pubchem.ncbi.nlm.nih.gov/compound/440735) |
| 702 | [Ellagic acid](https://cb.imsc.res.in/imppat/phytochemical-detailedpage/IMPHY005537) | Oc1cc2c(=O)oc3c4c2c(c1O)oc(=O)c4cc(c3O)O | [5281855](https://pubchem.ncbi.nlm.nih.gov/compound/5281855) |
| 703 | [Lactic acid](https://cb.imsc.res.in/imppat/phytochemical-detailedpage/IMPHY006327) | OC(=O)C(O)C | [612](https://pubchem.ncbi.nlm.nih.gov/compound/612) |
| 704 | [Malonic acid](https://cb.imsc.res.in/imppat/phytochemical-detailedpage/IMPHY006997) | OC(=O)CC(=O)O | [867](https://pubchem.ncbi.nlm.nih.gov/compound/867) |
| 705 | [3-(Galactosyloxy)-3',4',5,7-tetrahydroxyflavylium chloride](https://cb.imsc.res.in/imppat/phytochemical-detailedpage/IMPHY012458) | OC[C@H]1OC(Oc2cc3c(O)cc(cc3[o+]c2c2ccc(c(c2)O)O)O)[C@@H]([C@H]([C@H]1O)O)O.[Cl-] | [10299753](https://pubchem.ncbi.nlm.nih.gov/compound/10299753) |
| 706 | [Myrtenol](https://cb.imsc.res.in/imppat/phytochemical-detailedpage/IMPHY000099) | OCC1=CCC2CC1C2(C)C | [10582](https://pubchem.ncbi.nlm.nih.gov/compound/10582) |
| 707 | [O-Cymene](https://cb.imsc.res.in/imppat/phytochemical-detailedpage/IMPHY000545) | CC(c1ccccc1C)C | [10703](https://pubchem.ncbi.nlm.nih.gov/compound/10703) |
| 708 | [1,2,3-Trimethylbenzene](https://cb.imsc.res.in/imppat/phytochemical-detailedpage/IMPHY000581) | Cc1c(C)cccc1C | [10686](https://pubchem.ncbi.nlm.nih.gov/compound/10686) |
| 709 | [Chrysanthenone](https://cb.imsc.res.in/imppat/phytochemical-detailedpage/IMPHY000915) | CC1=CCC2C(=O)C1C2(C)C | [442463](https://pubchem.ncbi.nlm.nih.gov/compound/442463) |
| 710 | [Decane](https://cb.imsc.res.in/imppat/phytochemical-detailedpage/IMPHY001516) | CCCCCCCCCC | [15600](https://pubchem.ncbi.nlm.nih.gov/compound/15600) |
| 711 | [Piperitenone](https://cb.imsc.res.in/imppat/phytochemical-detailedpage/IMPHY003296) | CC1=CC(=O)C(=C(C)C)CC1 | [381152](https://pubchem.ncbi.nlm.nih.gov/compound/381152) |
| 712 | [4-Isopropylbenzaldehyde](https://cb.imsc.res.in/imppat/phytochemical-detailedpage/IMPHY003545) | O=Cc1ccc(cc1)C(C)C | [326](https://pubchem.ncbi.nlm.nih.gov/compound/326) |
| 713 | [Valeranone](https://cb.imsc.res.in/imppat/phytochemical-detailedpage/IMPHY003631) | CC([C@H]1CC[C@@]2([C@@](C1)(C)C(=O)CCC2)C)C | [171455](https://pubchem.ncbi.nlm.nih.gov/compound/171455) |
| 714 | [1,2,4-Trimethylbenzene](https://cb.imsc.res.in/imppat/phytochemical-detailedpage/IMPHY006039) | Cc1ccc(c(c1)C)C | [7247](https://pubchem.ncbi.nlm.nih.gov/compound/7247) |
| 715 | [Nerol oxide](https://cb.imsc.res.in/imppat/phytochemical-detailedpage/IMPHY006352) | CC(=CC1OCC=C(C1)C)C | [61275](https://pubchem.ncbi.nlm.nih.gov/compound/61275) |
| 716 | [Tricyclene](https://cb.imsc.res.in/imppat/phytochemical-detailedpage/IMPHY006950) | CC12C3C1CC(C2(C)C)C3 | [79035](https://pubchem.ncbi.nlm.nih.gov/compound/79035) |
| 717 | [1-Methyl-4-(prop-1-en-2-yl)benzene](https://cb.imsc.res.in/imppat/phytochemical-detailedpage/IMPHY008150) | Cc1ccc(cc1)C(=C)C | [62385](https://pubchem.ncbi.nlm.nih.gov/compound/62385) |
| 718 | [Bicyclo[3.1.1]hept-2-en-6-ol, 2,7,7-trimethyl-, (1S,5R,6R)-](https://cb.imsc.res.in/imppat/phytochemical-detailedpage/IMPHY009698) | CC1=CC[C@H]2[C@H]([C@@H]1C2(C)C)O | [10888100](https://pubchem.ncbi.nlm.nih.gov/compound/10888100) |
| 719 | [Salvial-4(14)-en-1-one](https://cb.imsc.res.in/imppat/phytochemical-detailedpage/IMPHY009709) | C=C1CCC(=O)[C@]2([C@@H](C1)[C@@H](CC2)C(C)C)C | [42608172](https://pubchem.ncbi.nlm.nih.gov/compound/42608172) |
| 720 | [Dodecane](https://cb.imsc.res.in/imppat/phytochemical-detailedpage/IMPHY010000) | CCCCCCCCCCCC | [8182](https://pubchem.ncbi.nlm.nih.gov/compound/8182) |
| 721 | [6-Methylhept-5-en-2-ol](https://cb.imsc.res.in/imppat/phytochemical-detailedpage/IMPHY010086) | CC(CCC=C(C)C)O | [20745](https://pubchem.ncbi.nlm.nih.gov/compound/20745) |
| 722 | [(-)-beta-Chamigrene](https://cb.imsc.res.in/imppat/phytochemical-detailedpage/IMPHY010179) | CC1=CC[C@@]2(CC1)C(=C)CCCC2(C)C | [442353](https://pubchem.ncbi.nlm.nih.gov/compound/442353) |
| 723 | [trans-Chrysanthenyl acetate](https://cb.imsc.res.in/imppat/phytochemical-detailedpage/IMPHY010635) | CC(=O)O[C@@H]1[C@@H]2CC=C([C@H]1C2(C)C)C | [10899521](https://pubchem.ncbi.nlm.nih.gov/compound/10899521) |
| 724 | [Tetradecane](https://cb.imsc.res.in/imppat/phytochemical-detailedpage/IMPHY011215) | CCCCCCCCCCCCCC | [12389](https://pubchem.ncbi.nlm.nih.gov/compound/12389) |
| 725 | [alpha-Campholenal](https://cb.imsc.res.in/imppat/phytochemical-detailedpage/IMPHY011407) | O=CC[C@H]1CC=C(C1(C)C)C | [1252759](https://pubchem.ncbi.nlm.nih.gov/compound/1252759) |
| 726 | [Neryl propionate](https://cb.imsc.res.in/imppat/phytochemical-detailedpage/IMPHY011449) | CCC(=O)OC/C=C(CCC=C(C)C)/C | [5365982](https://pubchem.ncbi.nlm.nih.gov/compound/5365982) |
| 727 | [Neryl acetate](https://cb.imsc.res.in/imppat/phytochemical-detailedpage/IMPHY011648) | C/C(=C/COC(=O)C)/CCC=C(C)C | [1549025](https://pubchem.ncbi.nlm.nih.gov/compound/1549025) |
| 728 | [gamma-Eudesmol](https://cb.imsc.res.in/imppat/phytochemical-detailedpage/IMPHY011938) | CC1=C2C[C@@H](CC[C@]2(CCC1)C)C(O)(C)C | [6432005](https://pubchem.ncbi.nlm.nih.gov/compound/6432005) |
| 729 | [Mesitylene](https://cb.imsc.res.in/imppat/phytochemical-detailedpage/IMPHY012088) | Cc1cc(C)cc(c1)C | [7947](https://pubchem.ncbi.nlm.nih.gov/compound/7947) |
| 730 | [4,7,7-Trimethylbicyclo[3.2.0]hept-3-en-6-one](https://cb.imsc.res.in/imppat/phytochemical-detailedpage/IMPHY014241) | CC1=CCC2C1C(=O)C2(C)C | [12309890](https://pubchem.ncbi.nlm.nih.gov/compound/12309890) |
| 731 | [beta-Selinene](https://cb.imsc.res.in/imppat/phytochemical-detailedpage/IMPHY014708) | C=C1CCC[C@]2([C@H]1C[C@@H](CC2)C(=C)C)C | [442393](https://pubchem.ncbi.nlm.nih.gov/compound/442393) |
| 732 | [Isobornyl 2-methylbutyrate](https://cb.imsc.res.in/imppat/phytochemical-detailedpage/IMPHY015753) | CCC(C(=O)OC1CC2C(C1(C)CC2)(C)C)C | [22082179](https://pubchem.ncbi.nlm.nih.gov/compound/22082179) |
| 733 | [trans-Sabinene hydrate](https://cb.imsc.res.in/imppat/phytochemical-detailedpage/IMPHY016027) | CC([C@@]12CC[C@](C2C1)(C)O)C | [12315151](https://pubchem.ncbi.nlm.nih.gov/compound/12315151) |
| 734 | [2-Methylocta-2,4,6-triene](https://cb.imsc.res.in/imppat/phytochemical-detailedpage/IMPHY016275) | CC=CC=CC=C(C)C | [53643885](https://pubchem.ncbi.nlm.nih.gov/compound/53643885) |
| 735 | [Bornyl valerate](https://cb.imsc.res.in/imppat/phytochemical-detailedpage/IMPHY016540) | CCCCC(=O)OC1CC2C(C1(C)CC2)(C)C | [110801](https://pubchem.ncbi.nlm.nih.gov/compound/110801) |
| 736 | [alpha-Phellandrene epoxide](https://cb.imsc.res.in/imppat/phytochemical-detailedpage/IMPHY017329) | CC(C1C=CC2(C(C1)O2)C)C | [527087](https://pubchem.ncbi.nlm.nih.gov/compound/527087) |
| 737 | [Caryophylla-4(14),8(15)-dien-5beta-ol](https://cb.imsc.res.in/imppat/phytochemical-detailedpage/IMPHY017614) | C=C1CC[C@@H](O)C(=C)CCCC(CC1)(C)C | [6429048](https://pubchem.ncbi.nlm.nih.gov/compound/6429048) |
| 738 | [2,7,7-Trimethylbicyclo[3.1.1]hept-2-en-6-yl acetate](https://cb.imsc.res.in/imppat/phytochemical-detailedpage/IMPHY014858) | CC(=O)OC1C2CC=C(C1C2(C)C)C | [162747](https://pubchem.ncbi.nlm.nih.gov/compound/162747) |
| 739 | [Parthenolide](https://cb.imsc.res.in/imppat/phytochemical-detailedpage/IMPHY006811) | C/C/1=CCC[C@@]2(C)O[C@@H]2[C@@H]2[C@@H](CC1)C(=C)C(=O)O2 | [7251185](https://pubchem.ncbi.nlm.nih.gov/compound/7251185) |
| 740 | [Artecanin](https://cb.imsc.res.in/imppat/phytochemical-detailedpage/IMPHY001062) | C=C1C(=O)O[C@H]2[C@H]1CC[C@@]([C@@]13[C@@H]2[C@@]2(C)O[C@H]2[C@H]3O1)(C)O | [442147](https://pubchem.ncbi.nlm.nih.gov/compound/442147) |
| 741 | [Germacrene a](https://cb.imsc.res.in/imppat/phytochemical-detailedpage/IMPHY003694) | C/C/1=CCC/C(=C/C[C@@H](CC1)C(=C)C)/C | [9548705](https://pubchem.ncbi.nlm.nih.gov/compound/9548705) |
| 742 | [(-)-Germacrene A](https://cb.imsc.res.in/imppat/phytochemical-detailedpage/IMPHY003695) | C/C/1=CCC/C(=C/C[C@H](CC1)C(=C)C)/C | [9548706](https://pubchem.ncbi.nlm.nih.gov/compound/9548706) |
| 743 | [Costunolide](https://cb.imsc.res.in/imppat/phytochemical-detailedpage/IMPHY003800) | C/C/1=C[C@H]2OC(=O)C(=C)[C@@H]2CC/C(=C/CC1)/C | [5281437](https://pubchem.ncbi.nlm.nih.gov/compound/5281437) |
| 744 | [Isofraxidin](https://cb.imsc.res.in/imppat/phytochemical-detailedpage/IMPHY005022) | COc1c(O)c(OC)cc2c1oc(=O)cc2 | [5318565](https://pubchem.ncbi.nlm.nih.gov/compound/5318565) |
| 745 | [Santamarine](https://cb.imsc.res.in/imppat/phytochemical-detailedpage/IMPHY005334) | CC1=CC[C@H]([C@]2([C@H]1[C@H]1OC(=O)C(=C)[C@@H]1CC2)C)O | [188297](https://pubchem.ncbi.nlm.nih.gov/compound/188297) |
| 746 | [Artemorin](https://cb.imsc.res.in/imppat/phytochemical-detailedpage/IMPHY005576) | C/C/1=C[C@H]2OC(=O)C(=C)[C@@H]2CCC(=C)[C@@H](CC1)O | [5281428](https://pubchem.ncbi.nlm.nih.gov/compound/5281428) |
| 747 | [Tanacetin](https://cb.imsc.res.in/imppat/phytochemical-detailedpage/IMPHY005777) | O=C1O[C@H]2[C@H](C1=C)CC[C@@]1([C@]2(O)C(=C)CC[C@H]1O)C | [20055042](https://pubchem.ncbi.nlm.nih.gov/compound/20055042) |
| 748 | [Alantolactone](https://cb.imsc.res.in/imppat/phytochemical-detailedpage/IMPHY006053) | C[C@H]1CCC[C@]2(C1=C[C@H]1[C@@H](C2)OC(=O)C1=C)C | [72724](https://pubchem.ncbi.nlm.nih.gov/compound/72724) |
| 749 | [Linalyl acetate](https://cb.imsc.res.in/imppat/phytochemical-detailedpage/IMPHY007067) | C=CC(OC(=O)C)(CCC=C(C)C)C | [8294](https://pubchem.ncbi.nlm.nih.gov/compound/8294) |
| 750 | [Magnolialide](https://cb.imsc.res.in/imppat/phytochemical-detailedpage/IMPHY008207) | CC1=C2[C@H]3OC(=O)C(=C)[C@@H]3CC[C@]2([C@@H](CC1)O)C | [636954](https://pubchem.ncbi.nlm.nih.gov/compound/636954) |
| 751 | [Reynosin](https://cb.imsc.res.in/imppat/phytochemical-detailedpage/IMPHY008745) | C=C1CC[C@H]([C@]2([C@H]1[C@H]1OC(=O)C(=C)[C@@H]1CC2)C)O | [482788](https://pubchem.ncbi.nlm.nih.gov/compound/482788) |
| 752 | [Cynaroside](https://cb.imsc.res.in/imppat/phytochemical-detailedpage/IMPHY011646) | OC[C@H]1O[C@@H](Oc2cc(O)c3c(c2)oc(cc3=O)c2ccc(c(c2)O)O)[C@@H]([C@H]([C@@H]1O)O)O | [5280637](https://pubchem.ncbi.nlm.nih.gov/compound/5280637) |
| 753 | [Apigenin 7-glucuronide](https://cb.imsc.res.in/imppat/phytochemical-detailedpage/IMPHY011711) | OC(=O)[C@H]1O[C@@H](Oc2cc(O)c3c(c2)oc(cc3=O)c2ccc(cc2)O)[C@@H]([C@H]([C@@H]1O)O)O | [5319484](https://pubchem.ncbi.nlm.nih.gov/compound/5319484) |
| 754 | [Sabinene hydrate](https://cb.imsc.res.in/imppat/phytochemical-detailedpage/IMPHY012205) | CC(C12CCC(C2C1)(C)O)C | [62367](https://pubchem.ncbi.nlm.nih.gov/compound/62367) |
| 755 | [Santin](https://cb.imsc.res.in/imppat/phytochemical-detailedpage/IMPHY012746) | COc1ccc(cc1)c1oc2cc(O)c(c(c2c(=O)c1OC)O)OC | [5281695](https://pubchem.ncbi.nlm.nih.gov/compound/5281695) |
| 756 | [Luteolin-7-glucuronide](https://cb.imsc.res.in/imppat/phytochemical-detailedpage/IMPHY012872) | OC(=O)[C@H]1OC(Oc2cc(O)c3c(c2)oc(cc3=O)c2ccc(c(c2)O)O)[C@@H]([C@H]([C@@H]1O)O)O | [5488307](https://pubchem.ncbi.nlm.nih.gov/compound/5488307) |
| 757 | [(+)-cis-Sabinol](https://cb.imsc.res.in/imppat/phytochemical-detailedpage/IMPHY015062) | CC([C@]12C[C@@H]2C(=C)[C@@H](C1)O)C | [94147](https://pubchem.ncbi.nlm.nih.gov/compound/94147) |
| 758 | [Butylbenzene](https://cb.imsc.res.in/imppat/phytochemical-detailedpage/IMPHY015496) | CCCCc1ccccc1 | [7705](https://pubchem.ncbi.nlm.nih.gov/compound/7705) |
| 759 | [Costic acid methyl ester](https://cb.imsc.res.in/imppat/phytochemical-detailedpage/IMPHY015575) | COC(=O)C(=C)[C@@H]1CC[C@@]2([C@@H](C1)C(=C)CCC2)C | [14707110](https://pubchem.ncbi.nlm.nih.gov/compound/14707110) |
| 760 | [Isoamyl isovalerate](https://cb.imsc.res.in/imppat/phytochemical-detailedpage/IMPHY015749) | CC(CCOC(=O)CC(C)C)C | [12613](https://pubchem.ncbi.nlm.nih.gov/compound/12613) |
| 761 | [Melatonin](https://cb.imsc.res.in/imppat/phytochemical-detailedpage/IMPHY015811) | COc1ccc2c(c1)c(CCNC(=O)C)c[nH]2 | [896](https://pubchem.ncbi.nlm.nih.gov/compound/896) |
| 762 | [9-epi-Pectachol B](https://cb.imsc.res.in/imppat/phytochemical-detailedpage/IMPHY015880) | COc1cc2ccc(=O)oc2c(c1OC[C@H]1C(=C)CC[C@@H]2[C@]1(C)CC[C@@H](C2(C)C)O)OC | [102328535](https://pubchem.ncbi.nlm.nih.gov/compound/102328535) |
| 763 | [Tanaparthin-alpha-peroxide](https://cb.imsc.res.in/imppat/phytochemical-detailedpage/IMPHY015944) | C=C1C(=O)O[C@H]2[C@H]1CC[C@@]([C@]13[C@@H]2[C@@](C)(OO1)C=C3)(C)O | [14219461](https://pubchem.ncbi.nlm.nih.gov/compound/14219461) |
| 764 | [secotanapartholide A](https://cb.imsc.res.in/imppat/phytochemical-detailedpage/IMPHY015945) | CC(=O)CC[C@H]1C(=C)C(=O)O[C@@H]1[C@@H]1C(=O)C=C[C@@]1(C)O | [10356188](https://pubchem.ncbi.nlm.nih.gov/compound/10356188) |
| 765 | tanetin | COc1ccc(cc1)c1oc2cc(OC)c(c(c2c(=O)c1OC)O)O | [10043097](https://pubchem.ncbi.nlm.nih.gov/compound/10043097) |
| 766 | [Tanaparthin-beta-peroxide](https://cb.imsc.res.in/imppat/phytochemical-detailedpage/IMPHY016032) | C=C1C(=O)OC2C1CCC(C13C2C(C)(OO1)C=C3)(C)O | [14219460](https://pubchem.ncbi.nlm.nih.gov/compound/14219460) |
| 767 | [secotanapartholide B](https://cb.imsc.res.in/imppat/phytochemical-detailedpage/IMPHY016033) | CC(=O)CC[C@H]1C(=C)C(=O)O[C@@H]1[C@@H]1C(=O)C=C[C@]1(C)O | [10265551](https://pubchem.ncbi.nlm.nih.gov/compound/10265551) |
| 768 | [Acetic acid;1,7,7-trimethylbicyclo[2.2.1]heptan-2-ol](https://cb.imsc.res.in/imppat/phytochemical-detailedpage/IMPHY017672) | OC1CC2C(C1(C)CC2)(C)C.CC(=O)O | [67173459](https://pubchem.ncbi.nlm.nih.gov/compound/67173459) |
| 769 | [(E)-4-[12-hydroxy-8,8,21,21-tetramethyl-5-(3-methylbut-2-enyl)-14,18-dioxo-3,7,20-trioxahexacyclo[15.4.1.02,15.02,19.04,13.06,11]docosa-4(13),5,9,11,15-pentaen-19-yl]-2-methylbut-2-enoic acid](https://cb.imsc.res.in/imppat/phytochemical-detailedpage/IMPHY014090) | CC(=CCc1c2OC34C(=CC5CC3C(OC4(C/C=C(/C(=O)O)C)C5=O)(C)C)C(=O)c2c(c2c1OC(C)(C)C=C2)O)C | [5366120](https://pubchem.ncbi.nlm.nih.gov/compound/5366120) |
| 770 | [Morellic acid](https://cb.imsc.res.in/imppat/phytochemical-detailedpage/IMPHY015014) | CC(=CCc1c2O[C@]34C(=C[C@@H]5C[C@H]3C(O[C@@]4(C/C=C(C(=O)O)/C)C5=O)(C)C)C(=O)c2c(c2c1OC(C)(C)C=C2)O)C | [54580250](https://pubchem.ncbi.nlm.nih.gov/compound/54580250) |
| 771 | gambogic acid | CC(=CCc1c2O[C@]34[C@@]5(C/C=C(C(=O)O)/C)OC(C4C[C@H](C5=O)C=C3C(=O)c2c(c2c1O[C@](C)(CCC=C(C)C)C=C2)O)(C)C)C | [11599836](https://pubchem.ncbi.nlm.nih.gov/compound/11599836) |
| 772 | [Neogambogic acid](https://cb.imsc.res.in/imppat/phytochemical-detailedpage/IMPHY006522) | CC(=CCc1c2OC34C(=CC5CC3C(OC4(C/C=C(/C(=O)O)C)C5=O)(C)C)C(=O)c2c(c2c1OC(C)(CCC=C(C)C)CC2O)O)C | [6438568](https://pubchem.ncbi.nlm.nih.gov/compound/6438568) |
| 773 | [Garcinolic acid](https://cb.imsc.res.in/imppat/phytochemical-detailedpage/IMPHY007241) | CC(=CCc1c2OC34C(=CCCC3C(O[C@@]4(C/C=C(/C(=O)O)C)C(=O)O)(C)C)C(=O)c2c(c2c1OC(C)(CCC=C(C)C)C=C2)O)C | [6857794](https://pubchem.ncbi.nlm.nih.gov/compound/6857794) |
| 774 | [Combretastatin D-1](https://pubchem.ncbi.nlm.nih.gov/compound/353314) | O=C1CCc2ccc(O)c(c2)Oc2ccc(cc2)[C@@H]2O[C@@H]2CO1 | [353314](https://pubchem.ncbi.nlm.nih.gov/compound/353314) |
| 775 | [Combretastatin A3](https://pubchem.ncbi.nlm.nih.gov/compound/353314) | COc1ccc(CCc2cc(O)c(OC)c(OC)c2)cc1O | [11722558](https://pubchem.ncbi.nlm.nih.gov/compound/11722558) |
| 776 | 5-[(1z)-2-(3-hydroxy-4-methoxyphenyl)ethenyl]-2,3-dimethoxyphenol | COc1ccc(/C=C\c2cc(O)c(OC)c(OC)c2)cc1O | [6444140](https://pubchem.ncbi.nlm.nih.gov/compound/6444140) |
| 777 | Combretastatin a-1 | COc1ccc(/C=C/c2cc(OC)c(OC)c(OC)c2)c(O)c1O | [6078282](https://pubchem.ncbi.nlm.nih.gov/compound/6078282) |
| 778 | 3,4,6,7-tetramethoxy-9,10-dihydrophenanthren-2-ol | COc1cc2c(cc1OC)-c1c(cc(O)c(OC)c1OC)CC2 | [14049975](https://pubchem.ncbi.nlm.nih.gov/compound/14049975) |
| 779 | [Combretastatin D-2](https://pubchem.ncbi.nlm.nih.gov/compound/353314) | O=C1CCc2ccc(O)c(c2)Oc2ccc(cc2)/C=C\CO1 | [11779322](https://pubchem.ncbi.nlm.nih.gov/compound/11779322) |
| 780 | [Combretastatin B-3](https://pubchem.ncbi.nlm.nih.gov/compound/353314) | COc1cc(CCc2ccc(O)c(O)c2)cc(OC)c1OC | [122775](https://pubchem.ncbi.nlm.nih.gov/compound/122775) |
| 781 | [Combretastatin B-4](https://pubchem.ncbi.nlm.nih.gov/compound/353314) | COc1cc(CCc2ccc(O)c(O)c2)cc(OC)c1 | [146633](https://pubchem.ncbi.nlm.nih.gov/compound/146633) |
| 782 | 5-[2-(3,5-dimethoxyphenyl)ethyl]-2-methoxyphenol | COc1cc(CCc2ccc(OC)c(O)c2)cc(OC)c1 | [153349](https://pubchem.ncbi.nlm.nih.gov/compound/153349) |
| 783 | [Combretastatin D1](https://pubchem.ncbi.nlm.nih.gov/compound/353314) | O=C1CCc2ccc(O)c(c2)Oc2ccc(cc2)C2OC2CO1 | [5173439](https://pubchem.ncbi.nlm.nih.gov/compound/5173439) |
| 784 | Combretastatin a-2 | COc1ccc(/C=C\c2cc(OC)c3c(c2)OCO3)cc1O | [11779540](https://pubchem.ncbi.nlm.nih.gov/compound/11779540) |
| 785 | 3,4,6-trimethoxy-9,10-dihydrophenanthrene-2,7-diol | COc1cc2c(cc1O)CCc1cc(O)c(OC)c(OC)c1-2 | [181637](https://pubchem.ncbi.nlm.nih.gov/compound/181637) |
| 786 | Combretastatin | COc1ccc(CC(O)c2cc(OC)c(OC)c(OC)c2)cc1O | [335929](https://pubchem.ncbi.nlm.nih.gov/compound/335929) |
| 787 | Combretastatin a-4 | COc1ccc(/C=C/c2cc(OC)c(OC)c(OC)c2)cc1O | [5386397](https://pubchem.ncbi.nlm.nih.gov/compound/5386397) |
| 788 | [Combretastatin D-1](https://pubchem.ncbi.nlm.nih.gov/compound/353314) | O=C1CCc2ccc(O)c(c2)Oc2ccc(cc2)[C@H]2O[C@@H]2CO1 | [71719106](https://pubchem.ncbi.nlm.nih.gov/compound/71719106) |
| 789 | 3,5,6,7-tetramethoxy-9,10-dihydrophenanthren-2-ol | COc1cc2c(cc1O)CCc1cc(OC)c(OC)c(OC)c1-2 | [14049974](https://pubchem.ncbi.nlm.nih.gov/compound/14049974) |
| 790 | Acacetin | COc1ccc(-c2cc(=O)c3c(O)cc(O)cc3o2)cc1 | [5280442](https://pubchem.ncbi.nlm.nih.gov/compound/5280442) |
| 791 | Combrestatin a4 | COc1ccc(/C=C\c2cc(OC)c(OC)c(OC)c2)cc1O | [5351344](https://pubchem.ncbi.nlm.nih.gov/compound/5351344) |
| 792 | Combrestatin a5 | COc1ccc(/C=C\c2cc(O)c(OC)c(OC)c2)cc1OC | [5386528](https://pubchem.ncbi.nlm.nih.gov/compound/5386528) |
| 793 | 5,6,7-trimethoxy-9,10-dihydrophenanthrene-2,3-diol | COc1cc2c(c(OC)c1OC)-c1cc(O)c(O)cc1CC2 | [14049973](https://pubchem.ncbi.nlm.nih.gov/compound/14049973) |
| 794 | Isoamoenylin | COc1cc(CCc2ccc(O)cc2)cc(OC)c1OC | [3084744](https://pubchem.ncbi.nlm.nih.gov/compound/3084744) |
| 795 | 2-methoxy-5-[(1e)-2-(7-methoxy-2h-1,3-benzodioxol-5-yl)ethenyl]phenol | COc1ccc(/C=C/c2cc(OC)c3c(c2)OCO3)cc1O | [5386527](https://pubchem.ncbi.nlm.nih.gov/compound/5386527) |
| 796 | 5-[(2s)-2-hydroxy-2-(3,4,5-trimethoxyphenyl)ethyl]-2-methoxyphenol | COc1ccc(C[C@H](O)c2cc(OC)c(OC)c(OC)c2)cc1O | [100154](https://pubchem.ncbi.nlm.nih.gov/compound/100154) |
| 797 | (-)-Combretastatin | COc1ccc(C[C@@H](O)c2cc(OC)c(OC)c(OC)c2)cc1O | [9895264](https://pubchem.ncbi.nlm.nih.gov/compound/9895264) |
| 798 | Combretastatin b-1 | COc1ccc(CCc2cc(OC)c(OC)c(OC)c2)c(O)c1O | [135716](https://pubchem.ncbi.nlm.nih.gov/compound/135716) |
| 799 | 3,5,6,7-tetramethoxyphenanthren-2-ol | COc1cc2c(ccc3cc(OC)c(OC)c(OC)c32)cc1O | [11141545](https://pubchem.ncbi.nlm.nih.gov/compound/11141545) |
| 800 | 5-[(1e)-2-(3,4-dimethoxyphenyl)ethenyl]-2,3-dimethoxyphenol | COc1ccc(/C=C/c2cc(O)c(OC)c(OC)c2)cc1OC | [5386529](https://pubchem.ncbi.nlm.nih.gov/compound/5386529) |


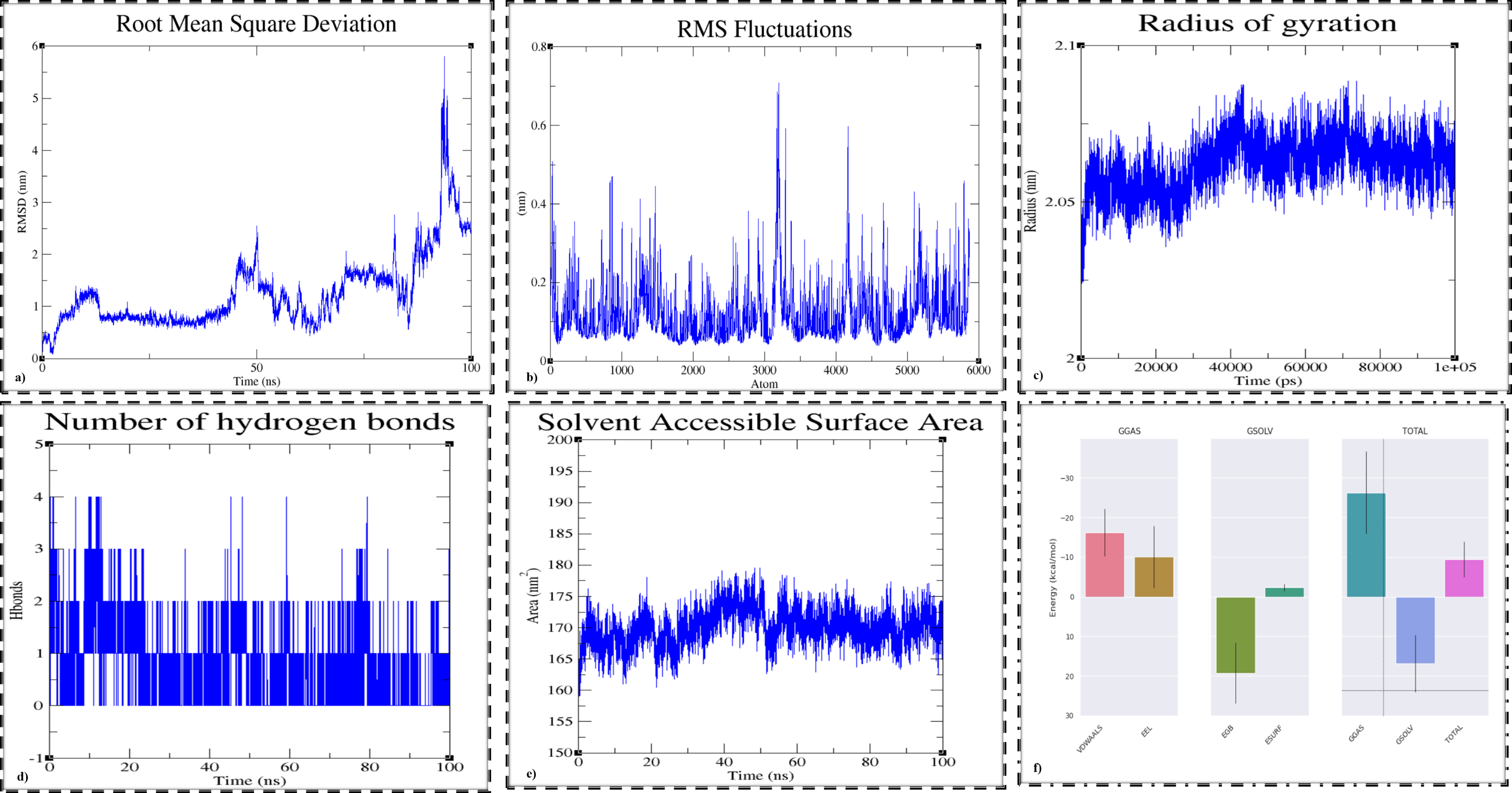


**Supplementary (Fig S1):** Post-MD simulation analysis of HDAC1-PEL. *A) RMSD plot B) RMSF plot*, *C) Rg plot of HDAC1, D) H-bond plot between, E) SASA plot, F) Total binding free energy profile.*
